# Exploring the stability of the NHC–metal bond using thiones as probes†

**DOI:** 10.1039/d1cc02740a

**Published:** 2021-09-10

**Authors:** Nathalie Ségaud, Chloë Johnson, Albert Farre, Martin Albrecht

**Affiliations:** Department of Chemistry & Biochemistry, University of Bern, Freiestrasse 3 3012 Bern Switzerland martin.albrecht@unibe.ch

## Abstract

The metal–carbon bond in N-heterocyclic carbene (NHC) metal complexes, which are ubiquitous in modern homogeneous catalysis, is often conjectured to be robust. Here, carbene dissociation was evaluated from a series of complexes with metals of relevance in catalysis containing either an Arduengo-type 2-imidazolylidene or a mesoionic 1,2,3-triazolylidene ligand through thione formation, revealing remarkable kinetic lability of the NHC–metal bond for, *e.g.* Ir^III^, Rh^III^, and Ni^II^ complexes.

## Abstract 2

The stability of Arduengo-type N-heterocyclic carbenes and mesoionic triazolylidenes to a range of catalytically active metals was probed. Remarkably labile bonding was observed with some metals, which impacts mechanistic considerations in catalysis.

## Graphical abstract

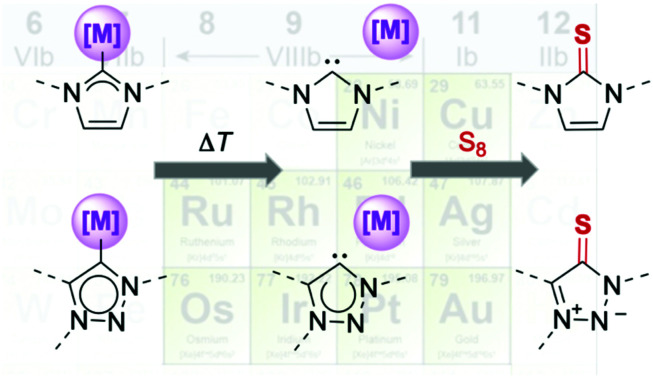

N-Heterocyclic carbenes (NHC)^1^ have become an ubiquitous and versatile class of ligands in organometallic chemistry,^2,3^ largely due to their impact as powerful spectator ligands in the development of NHC metal catalysts.^4–6^ Especially when coordinated to late d-block metals, NHC ligands have boosted the activity of homogeneous catalysts in a broad range of applications.^7–9^ Generally and often by default, the extraordinary activity imparted by NHCs has been attributed to the strong σ donor properties and to the high robustness of the NHC–metal bond, which is assumed because of the relatively high degree of covalent character of the M–C_NHC_ bond. This bonding model has been supported by several theoretical investigations,^10–12^ though relevant work has advised caution when conjecturing robust NHC coordination to the metal centre throughout a catalytic process.

In an early account, Crudden critically reviewed the stability of the metal–ligand bond of NHC complexes comprised of the most popular NHC ligands,^13^*i.e.* Arduengo-type imidazol-2-ylidenes.^14–17^ An array of decomposition pathways has been delineated including NHC ligand dissociation and substitution, migratory insertion and reductive elimination processes, which are dependent on the applied reaction conditions. More recently, Hahn and coworkers have used NHCs for supramolecular assemblies, which implies a thermodynamic control and hence reversible M–NHC bond formation.^18,19^ Some of these reactivity patterns have also been observed in complexes bearing 1,2,3-triazol-4-ylidenes,^20^ a subclass of mesoionic carbenes that emerged during the last decade.^21^ Degradation of some complexes was observed that involved carbene dissociation. Moreover, recent work pointed to an increased relevance of NHC dissociation for catalyst activation with a variety of metals.^22,23^ Here, we report on a systematic study to determine the stability of the NHC–metal bond. Specifically, we investigated a series of NHC metal complexes on their intrinsic stability, with a particular focus on potential carbene dissociation, which was monitored by trapping the released free carbene with elemental sulfur to form the corresponding thione (ESI†) as a readily detectable indirect probe.^24–26^ To this end, a range of NHC complexes were prepared with group 8–11 metals, as these metals are by far the most frequently used in catalytic applications, including Au(i), Pd(ii), Rh and Ir both in 1+ and 3+ oxidation states, Ru(ii), Ni(ii), and Ag(i), as well as less explored Pt(ii) and Os(ii) complexes (Fig. 1). The NHC ligands used were *N*,*N*′-dibutyl-imidazol-2-ylidene (**imi**) as a representative of the Arduengo-type NHCs, and 1,4-dibutyl-substituted 1,2,3-triazol-4-ylidene (**trz**) as a mesoionic carbene (Fig. 1). The identical set of wingtip groups minimises any steric bias between these two ligand systems and allows for electronic ligand effects to be compared directly. Since the majority of catalytic systems feature more stabilizing Mes or Dipp wingtip groups, also selected IMes complexes were investigated (IMes = 1,3-dimesityl-2-imidazolylidene).

**Fig. 1:**
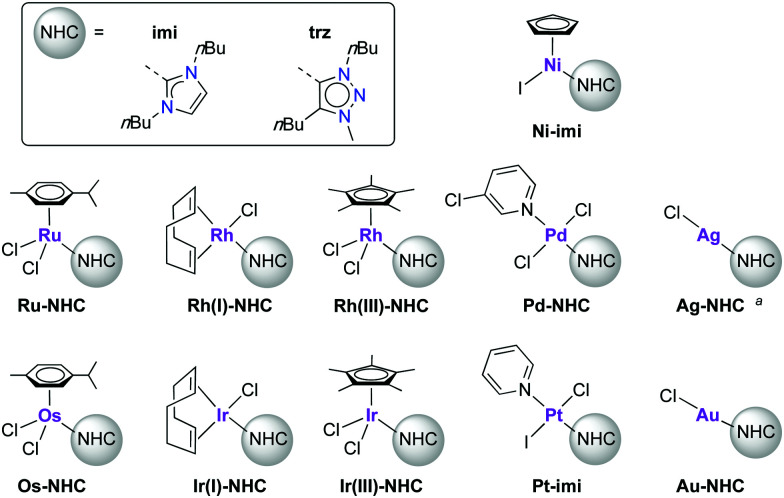
Complexes used in this study, bearing NHC ligands **imi** (1,3-(di-*n*-butyl)-imidazol-2-ylidene) or **trz** (1,4-(di-*n*-butyl)-3-methyl-1,2,3-triazolylidene). ^*a*^ Dynamic mixture of [Ag(NHC)_2_Cl] and [Ag(NHC)Cl].

Silver NHC complexes were used to validate the sulfur quenching methodology, as these complexes are known to undergo rapid carbene transfer for transmetalation and also internal carbene transfer through establishing an equilibrium between neutral (NHC)AgX and ionic [(NHC)_2_Ag](AgX_2_) species in solution (Scheme 1).^27,28^ Indeed, in the presence of 0.6 eq. S_8_, complex **Ag-imi** transformed quantitatively into the corresponding thione **imi = S** within 2 h at room temperature (1,2-dichlorobenzene solution, Scheme 1 and Fig. S19, ESI†) as indicated by a distinct shift of the N–CH_2_ resonances from 4.09 to 4.02 ppm in the ^1^H NMR spectrum (CD_2_Cl_2_). Similarly, the analogous thione **trz = S** formed quantitatively from **Ag-trz** (*δ*_NCH_2__ = 4.45 *vs.* 4.41; Fig. S20, ESI†). The reaction is faster with **Ag-imi** than with the **trz** analogue (89% *vs.* 55% conversion after 30 min; Table S1 and Fig. S1, ESI†), though after 2 h, both complexes are quantitatively transformed into the corresponding thione.

**Scheme 1:**
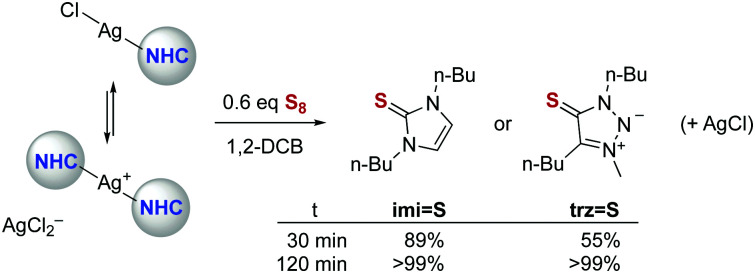
Formation of thiones **imi = S** and **trz = S** from the corresponding silver(i) NHC complexes, revealing slightly higher stability of the **Ag-trz** complex.

Based on this validation with the silver(i) complexes, we investigated the stability of a variety of **M-imi** complexes. No thione formation was observed with any of the investigated group 8–11 metal complexes at RT within 24 h, apart from **Ag-imi** as discussed above. However, slow degradation was observed with some complexes, *e.g.*, gradual dissociation of the spectator cymene from **Ru-imi** and especially COD from the only poorly stable **Ir(i)-imi**, as noted by NMR spectroscopy (Fig. S21, S22 and Table S2, ESI†). Decomposition was significant for **Rh(i)-imi** and higher for the nickel(ii) and osmium(ii) complexes (Fig. S23–S25, ESI†). At room temperature, the M–NHC bond is thus stable towards dissociation except for NHC–Ag complexes.

When solutions of the complexes were heated to 120 °C, however, more distinct stability profiles became apparent, including (i) thione and imidazolium salt formation because of NHC dissociation, (ii) complex decomposition without thione formation, and (iii) dissociation reactions followed by thione formation (Scheme 2). Only the gold(i) complex **Au-imi** did not change at all and was completely stable for 24 h (Fig. 2, Table S3 and Fig. S33, ESI†). Imi complexes with Pd^II^, Pt^II^, and Ir^I^ underwent degradation, yet without any formation of thione or imidazolium salt, suggesting transformations involving the ancillary ligands of the complexes without M–C_imi_ bond cleavage. The degradation is slow for **Pd-imi** and **Pt-imi** (7% and 31%, respectively, within 24 h), and faster for **Ir(i)-imi** (100% in 6 h; Fig. S34–S37, ESI†). Imi complexes of Os^II^, Ru^II^, and Rh^I^ revealed some thione by NMR spectroscopy, though only after apparent modification of the initial complex, indicating labilisation of the M–C_imi_ bond after modification of the ancillary ligands. For example, heating **Os-imi** revealed loss of cymene after 30 min (13%) and 55% degradation after 24 h, but only 8% thione was formed (Fig. S39, ESI†). Similar reactivity was observed for **Ru-imi** (24% thione formed), while **Rh(i)-imi** lost COD much faster (78% decomposition after 30 min with 15% thione after 24 h, Fig. S37 and S38, ESI†). Finally, complexes **Rh(iii)-imi**, and **Ni-imi** produced considerable quantities of imidazolium salt and over time also thione (48% and full degradation, respectively, after 30 min), commensurate with the degradation of the original complex and pointing to carbene dissociation under these conditions (Fig. S41 and S42, ESI†). The same process was observed for **Ir(iii)-imi**, yet considerably slower (7% dissociation after 30 min; Fig. S40, ESI†). Indeed, a separate experiment demonstrated that the imidazolium salt [**imi-H**]I is gradually transformed to the thione **imi = S** in the presence of S_8_ (30% conversion in 24 h; Fig. S43, ESI†). Conversely, degradation in the absence of S8 is considerably slower, as demonstrated with **Rh(iii)-imi** (Fig. S57, ESI†), which may point to a fast rebound of the dissociated carbene, though a sulfur-induced decomposition cannot be ruled out either.

**Scheme 2:**
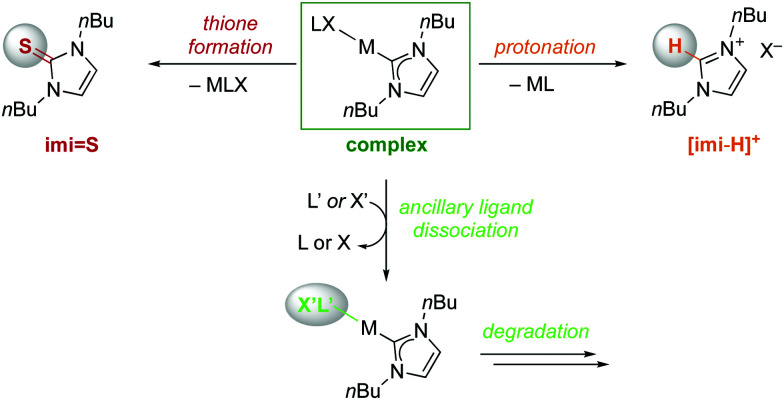
NHC dissociation pathways in **M-imi** complexes leading to either thione **imi = S** or imidazolium salt **[imi-H]+** and complex degradation *via* ancillary ligand dissociation.

**Fig. 2:**
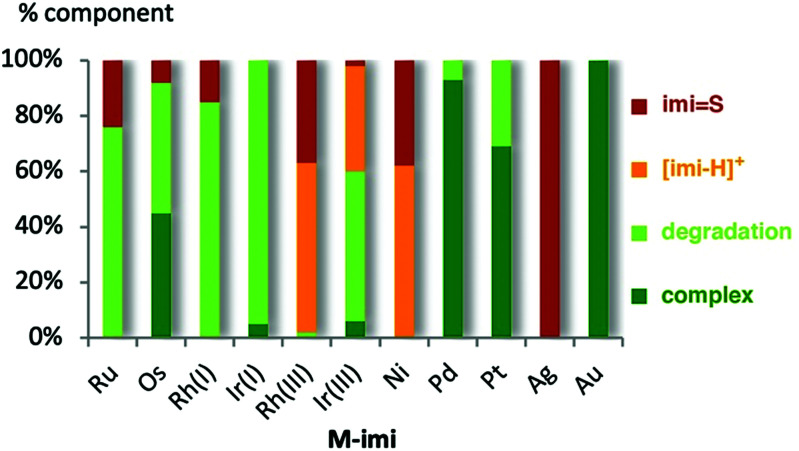
Amount of **M-imi** thione, imidazolium salt, and degradation products after 24 h reaction at 120 °C.

The different behaviour of **Ni-imi**, **Rh(iii)-imi** and also **Ir(iii)-imi** compared to **Ag-imi** through formation of predominantly **imi-H+** from the former complexes as opposed to **imi = S** from the silver carbene suggests that the NHC dissociation from **Ag-imi** is induced by sulfur binding to the metal, while in the former, NHC dissociation is assumed to be more spontaneous, with the free carbene quenched by H^+^ and to some extent by sulfur.

While the lability of **Ag-imi** and **Ni-imi** is not surprising since both systems have been used in transmetallation, *i.e.* carbene transfer reactions, the lability of **Rh(iii)-imi** and **Ir(iii)-imi** is remarkable. Presumably, the substantial carbene dissociation from the metal centre, as indicated by the formation of **imi = S** and **[imi-H]+**, may, in parts, originate from the mismatch of the soft carbene ligand with the relatively hard d^6^ metal configuration. Consistent with this consideration, also the Ru(ii) complex reveals considerable carbene dissociation.

Modification of the N-substituent of the carbene from butyl to the catalytically more frequently used mesityl group has been investigated with a selection of metals which were relatively poorly stable as **imi** variants, namely **Ag-IMes**, **Ni-IMes**, **Rh(i)-IMes**, and **Ir(i)-IMes**. The stability profile of these complexes showed surprisingly little differences to their **imi** analogues (Fig. S53–S56 and Table S4, ESI†). **Ir(i)-IMes** is slightly more stable than **Ir(i)-imi**, whereas this trend is opposite for Ni(ii).

Expansion of these stability studies to mesoionic 1,2,3-triazolylidene complexes^20–22^ reveals similar stability properties at room temperature, and thione formation was observed only with complex **Ag-trz** (Table S5, ESI†). The **Rh(iii)**, **Ir(iii)**, and **Pd-trz** complexes are completely stable over 24 h. Other complexes degraded slowly over time without formation of thione, involving either dissociation of ancillary ligands (**Ru-trz**, **Ir(i)-trz**) or overall decomposition (**Rh(i)** and **Os-trz**), though no sign of trz–metal bond cleavage was detected (Fig. S26–S32, ESI†). These data indicate a stable M–C_trz_ bond in all complexes but the silver system. Notably, the overall complex stability is influenced by the nature of the NHC and the metal and is higher for Ag complexes bound to **trz** than to **imi**, while the inverse was noted for Ru and Ir complexes. Ancillary ligand dissociation in these complexes is slower in the **imi** analogues.

As noted for complexes bearing **imi** ligands, stability differences in the **M-trz** series were only observed at elevated temperatures. At 120 °C, the same four reactivity patterns were observed (Scheme 3; Table S6 and Fig. S44–S52, ESI†). The gold(i) complex **Au-trz** was completely stable and no modification was observed over 24 h (Fig. 3). Complexes with Pd^II^ and Ir^I^ decomposed without formation of thione nor triazolium salt, as observed for their **imi** analogues but at different rates. The degradation is slow for **Pd-trz** (5% within 24 h) and fast for **Ir(i)-trz** (100% in 30 min; Fig. S45 and S46, ESI†). Trz complexes of Os^II^, Ru^II^ and Rh^I^ revealed some thione formation after modification of the initial complex. As observed for their **imi** analogues, loss of ancillary ligands was already observed after 30 min and almost full degradation after 24 h, with only 22–50% thione formed. Loss of cymene was observed in **Os-trz** after 30 min (20%) and almost full degradation after 24 h (28% thione, Fig. S49, ESI†). Similarly, **Ru-trz** slowly lost *p*-cymene (22% after 30 min) and fully decomposed after 24 h (50% of thione), while **Rh(i)-trz** lost COD much faster (full decomposition after 30 min and 22% thione after 24 h, Fig. S47 and S48, ESI†). Finally, complex **Rh(iii)-trz** produced some quantities of triazolium salt and over time also thione (full degradation, 72% **[trz-H]+** and 23% **trz = S** after 6 h, Fig. S51, ESI†).‡‡As observed with **[imi-H]+**, the triazolium salt **[trz-H]+** is gradually converted into the thione **trz = S**, with a higher yield than the **imi** system (71% *vs.* 30% conversion in 24 h; Fig. S52, ESI†). Complex **Ir(iii)-trz** decomposed slower than the **Rh(iii)** analogue, with formation of thione but no triazolium salt (62% decomposition and 57% **trz = S** after 6 h, Fig. S50, ESI†).

**Scheme 3:**
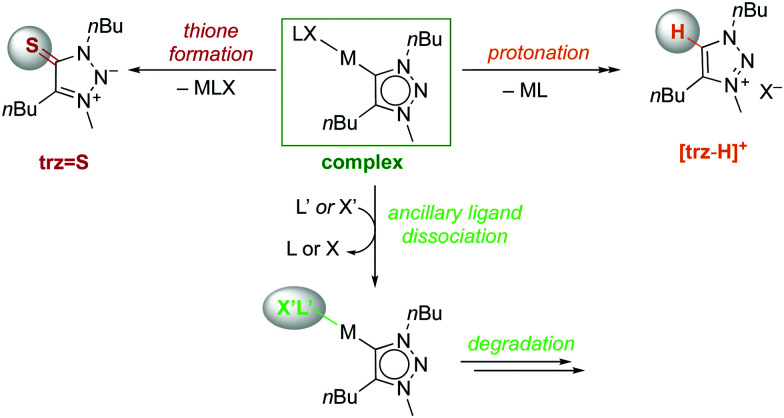
NHC dissociation pathways from **M-trz** complexes leading to thione **trz = S** or triazolium salt **[trz-H]+** and complex degradation *via* ancillary ligand dissociation.

**Fig. 3:**
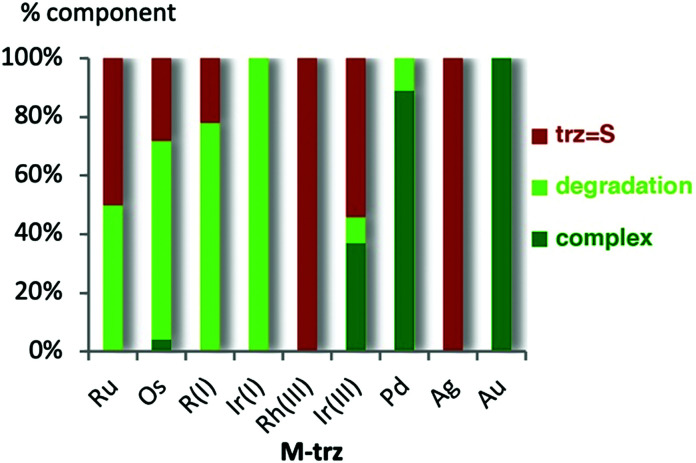
Amount of thione, starting complex, imidazolium salt, and degradation products after 24 h reaction at 120 °C, for **M-trz** complexes. Some triazolium salt **[trz-H]+** was observed transiently from **Rh(iii)-trz** after 30 min.

The nature of the NHC had distinct effects on the NHC–metal bond stability depending on the coordinated metal. The **trz** ligand is more labile in group 8 metal complexes (Ru, Os) while higher stability was observed for group 11 metal complexes (Ag; Fig. S2, ESI†). The increased stability of **trz** bonds to d^8^/d^10^ metals (Rh(i), Ir(i), Ag, Cu) as opposed to d^6^ metals (Ru, Os) is remarkable since **trz** ligands were shown to be stronger donors than **imi**,^29^ which would suggest tighter bonding with less electron-rich metal centres. Obviously, the NHC–metal bond stability may significantly deviate from the trends established here, *e.g.*, by wingtip group variations, by modified conditions such as different polarity of the solvent, by variation of the ancillary ligands and their electronic and steric properties, or by the presence of additives such as base or acid as used *e.g.* under catalytic conditions. For example, **Ir(iii)-trz**, which is catalytically active in transfer hydrogenation at 80 °C in basic iPrOH does not show any degradation at this temperature in dichlorobenzene (Fig. S58 and S59, ESI†). Nonetheless, these results indicate that the preservation of the NHC–metal bond should not be taken as granted and mechanistic proposals should be tested under relevant conditions for NHC ligand dissociation rather than assuming by default a reliable bonding of the NHC ligand to the metal centre throughout a catalytic cycle, as often assumed due to the relatively high covalent character of the M–NHC bond. These conclusions align well with earlier studies that have revealed M–NHC bond dissociation as the crucial step for catalyst activation.^23^ Limited M–NHC stability may also be a critical factor for the recently established anticancer activity of some NHC metal complexes.^30^

In summary, this work provides insights into the intrinsic stability of the NHC–metal bond of both Arduengo-type imidazolylidenes as well as mesoionic triazolylidenes bound to metal centres of catalytic relevance (Fig. S58, ESI†). Under the specific conditions and in the absence of any auxiliary or substrate, several complexes including Ir^III^, Rh^III^, and Ni^II^ showed kinetic lability of the carbene metal bond, with slightly better stability of triazolylidenes than imidazolylidenes. This intrinsic kinetic lability of the M–NHC bond has obvious ramifications in catalysis and needs to be considered in catalyst design. Moreover, caution is required when assuming a robust M–NHC bond in mechanistic proposals. This lability of the NHC–metal bond can be effectively mitigated, for example, by installing appropriate chelating groups on the NHC ligand.

We thank the Swiss National Science Foundation (200020-182633) for generous financial support and the Marie Sklodowska-Curie Action for a fellowship to N. S. (grant 796762).

## Conflicts of interest

There are no conflicts to declare.

## Supplementary Material

CC-057-D1CC02740A-s001
