# Supplementary material for: Exploring the stability of the NHC–metal bond using thiones as probes
Source: Chem Commun (Camb). 2021 Sep 10;57(81):10600–3. doi: 10.1039/d1cc02740a (PMC8506647; doi:10.1039/d1cc02740a)
Supplement: CC-057-D1CC02740A-s001 [file CC-057-D1CC02740A-s001.pdf]

Electronic Supplementary Material (ESI) for ChemComm.
This journal is © The Royal Society of Chemistry 2021

Electronic Supporting information for

Exploring the stability of the NHC–metal bond using thiones as probes

Nathalie Ségaud, Chloë Johnson, Albert Farre and Martin Albrecht*

Department of Chemistry & Biochemistry, University of Bern, Freiestrasse 3, 3012 Bern, Switzerland

Content:

1. Experimental details S2
2. NMR spectra of the complexes S6
3. Thione formation results S14
4. NMR spectra of the stability tests at room temperature S17
5. NMR spectra of the stability tests at 120 °C S24
6. Catalytic activity and stability of Ir(III)–trz S36
7. References S37

1. Experimental details

General comments. Solvents (THF, CH_2Cl_2 and CH_3CN) were dried by passage through solvent purification columns. Extra-dry 1,2-dichlorobenzene (1,2-DCB) in Sure/Seal bottles and all other reagents were ordered from Sigma-Aldrich and used without further purification. NMR spectra were measured at room temperature on Bruker spectrometers operating at 300 MHz (^{1}H NMR) or 75 MHz ($^{13}C\{H\}$ NMR). Chemical shifts (δ in ppm, coupling constants J in Hz) were referenced to residual solvent resonances. Assignments were made based on homo- and heteronuclear shift correlation spectroscopy. Elemental analyses and high-resolution ESI mass spectrometry were performed by the Mass Spectrometry Group in the University of Bern using a Flash 2000 Organic Elemental Analyzer (Thermo Scientific) and a LTQ Orbitrap XL with nano ESI (Thermo Scientific) respectively.

Complexes syntheses. Metalation reactions were carried out under an inert nitrogen atmosphere using standard Schlenk techniques unless otherwise specified. The metal precursors nickelocene,[S1] $[Ru(p\text{-cym})Cl_2]_2$,[S2] $[Os(p\text{-cym})Cl_2]_2$,[S3] $[Ir(COD)Cl]_2$,[S4] $[Rh(COD)Cl]_2$,[S5] $[Cp^*IrCl_2]_2$[S6] and $[Cp^*RhCl_2]_2$,[S6] and azolium salts **imi-H.I**[S7] and **trz-H.I**[S8] were prepared following literature procedures. The synthesis of **imi=S**,[S9] and the complexes **Ir(I)-imi**,[S10] **Ir(I)-imi**,[S11] **Rh(I)-imi**,[S12] **Rh(I)-trz**,[S8] **Ru-imi**,[S13] **Ru-trz**,[S14] **Ag-imi**,[S15] **Ag-trz**,[S16] **Au-imi**,[S17] **Au-trz**,[S18] **Pd-imi**,[S19] and **Pd-trz**[S20] as well as complexes **Ag-IMes**,[S21] **Ni-IMes**,[S22] **Rh(I)-IMes**,[S23] and **Ir(I)-IMes**[S24] have been previously reported.

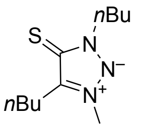

trz=S [1,4-(di-*n*-butyl)-3-methyl-5-thioxo-1,2,3-triazolylidene]. The **imi-H.I** salt (163 mg; 0.51 mmol) and KO*t*Bu (68 mg; 0.61 mmol) were suspended in THF (3 mL) and the reaction mixture stirred for 30 min at room temperature. Sulfur powder (17 mg; 0.53 mmol) was added, and the mixture stirred further for 1 h. The solution was collected by filtration through celite and the solvent removed in vacuo. The product was purified by SiO_2 column chromatography using a gradient of 1:100 to 1:20 $MeOH/CH_2Cl_2$. The thione was isolated as a pale-yellow oil upon evaporation of all volatiles under reduced pressure (92 mg; 79%). ^{1}H NMR ($CDCl_3$; 300 MHz): δ 4.35 (t, ^{3}J = 7.5 Hz, 2H, N–CH_2), 3.91 (s, 3H, N–CH_3), 2.73 (t, ^{3}J = 8.4 Hz, 2H, C_{trz}–CH_2), 1.93–1.77 (m, 2H, CH_2–CH_2N), 1.67–1.50 (m, 2H, CH_2–CH_2C_{trz}), 1.46–1.25 (m, 4H, CH_2), 0.91, 0.89 (2 x t, ^{3}J = 7.2 Hz, 3H, CH_3) ppm. $^{13}C\{H\}$ NMR ($CDCl_3$; 75 MHz): δ 155.0 (C=S), 138.5 (C_{trz}–CH_2), 48.3 (N–CH_2), 36.8 (N–CH_3), 30.1 (CH_2–CH_2N), 29.1 (CH_2–CH_2C_{trz}), 24.1 (CH_2–C_{trz}), 22.6, 19.8 (2 x CH_2), 13.8, 13.6 (2 x CH_3) ppm. HR-MS (ESI): calcd for $C_{11}H_{21}N_3NaS$ $[M+Na]^+$ m/z = 250.1348, found m/z = 250.1347. Elem. anal. found (calcd) for $C_{11}H_{21}N_3S$ (227.37 g/mol): C 57.97 (58.11); H 9.24 (9.31); N 18.42 (18.48).

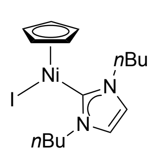

Ni-imi [(Cp)NiCl(imi)]. The **imi-H.I** salt (308 m, 1mmol) and nickelocene (189 mg; 1 mmol) were suspended in 1,4-dioxane (10 mL), and stirred at 90 °C for 3 h under nitrogen atmosphere. The solvent was removed *in vacuo* and a brown solution extracted with hot toluene (15 mL) and filtered through a glass microfiber filter. The toluene solution was concentrated (~ 5 mL) and loaded onto a SiO_2 column. Using Et_2O/pentane (1:1) as eluent, the second, red fraction was collected under nitrogen and evaporated to dryness *in vacuo* to afford the nickel carbene complex as a red-pink oil (39 mg; 9%) An analytically pure sample was obtained following recrystallisation from hexane. ^{1}H NMR ($CDCl_3$; 300 MHz): δ 6.93 (s, 2H, H_{imid}), 5.32 (s, 5H, H_{Cp}), 4.79, 4.52 (2 x ddd, ^{2}J = 13.7, ^{3}J = 9.5, 6.0 Hz, 2H, N–CH_2), 2.04–1.73 (m, 4H, CH_2–CH_2N), 1.57–1.43 (m, 4H, CH_2–CH_3), 1.04 (t, ^{3}J = 7.4 Hz, 6H, CH_3–CH_2) ppm. $^{13}C\{H\}$ NMR ($CDCl_3$; 75 MHz): δ 163.1 (C_{imid}–Ni), 122.1 (C_{imid}–H), 91.8 (C_{Cp}), 52.3 (N–CH_2), 32.9 (CH_2–CH_2N), 20.3 (CH_2–CH_3), 14.1 (CH_3–

CH_2) ppm. HR-MS (ESI): calcd for $C_{16}H_{25}N_2Ni$ [M−I]$^+$ m/z = 303.1371, found m/z = 303.1362. Elemental analysis could not be determined due to the rapid decomposition of the complex in air.

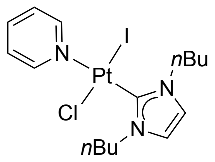

Pt-imi [(py)PtICl(imi)]. imi-H.I (100 mg, 0.32 mmol), potassium carbonate (450 mg, 3.2 mmol), sodium chloride (191 mg, 3.2 mmol) and $PtCl_2$ (88 mg, 0.33 mmol) were combined in a schlenk tube and pyridine (7 mL) was added. The resulting mixtue was stirred at 100 °C for 16h. The solution was filtered and evaporated to dryness *in vacuo*. The resulting orange oil was purified by column chromatography on SiO_2 (CH_2Cl_2 to CH_2Cl_2/acetonitrile, 4:1), yielding **Pt1** as a yellow powder (144 mg; 70%). ^{1}H NMR (300 MHz, $CDCl_3$): δ 9.03 (d, J = 5.0 Hz, 2H, CH_{py}), 7.72 (t, J = 7.6 Hz, 1H, CH_{py}), 7.32 (t, J = 7.0 Hz, 2H, CH_{py}), 6.85 (s, 2H, CH_{Im}), 4.44 (t, J = 7.7 Hz, 4H, N–CH_2), 2.09–1.94 (m, 4H, CH_2–CH_2N), 1.53–1.40 (m, 4H, CH_2–CH_3), 1.03 ppm (t, J = 7.3 Hz, 6H, CH_3–CH_2) ppm. ^{13}C NMR (75 MHz, $CDCl_3$): δ 153.9 (CH_{py}), 137.5 (CH_{py}), 134.5 (C_{Im}), 125.1 (CH_{py}), 120.5 (CH_{Im}), 50.9 (N–CH_2), 31.7 (CH_2–CH_2N), 20.1 (CH_2–CH_3), 13.9 ppm (CH_3–CH_2) ppm. HR-MS (ESI): calcd for $C_{16}H_{25}IN_3Pt$ [M−Cl]$^+$ m/z = 581.0741, found m/z = 581.0735. Elem. anal. found (calcd) for $C_{16}H_{25}ClIN_3Pt$ x 1.2 CH_2Cl_2 (733.05 g/mol): C 27.90 (28.18); H 3.33 (3.77); N 5.92 (5.73).

General transmetalation procedure. The relevant azolium salt (1.0 equiv.), Ag_2O (0.65 equiv.) and Me_4NCl (1.3 equivalents) were suspended in CH_2Cl_2 and stirred for 2 h under the exclusion of light. The solution was filtered through a glass microfiber filter into a CH_2Cl_2 solution of the relevant metal precursor (1 equivalent with respect to the metal amount) and the reaction mixture was stirred for further hours in the absence of light. At the end point of the reaction, the mixture was filtered through celite eluting with CH_2Cl_2, and all volatiles evaporated under reduced pressure to afford the crude metal complex.

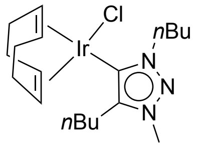

Ir(I)-trz [(COD)IrCl(trz)]. Following the general transmetalation procedure, using **trz-H.I** (65 mg; 0.20 mmol), Ag_2O (30 mg; 0.13 mmol) and Me_4NCl (31 mg; 0.28 mmol) in CH_2Cl_2 (5 mL) and $[Ir(COD)Cl]_2$ (67 mg; 0.10 mmol). The reaction mixture was stirred for 1 h. The waxy solid was washed several times with pentane until it turns solid and then dried *in vacuo* (95 mg; 92%). ^{1}H NMR ($CDCl_3$; 300 MHz): δ 4.71 (ddd, ^2J = 13.1 Hz, ^3J = 9.2, 6.1 Hz, 1H, CH_2–N) 4.56–4.37 (m, 3H, CH_2–N, CH_{COD}), 3.90 (s, 3H, N–CH_3), 2.98–2.72 (m, 4H, CH_2–C_{trz}, CH_{COD}), 2.22–1.84 (m, 6H, CH_2), 1.79–1.37 (m, 10H, CH_2), 1.01 (t, J = 7.3 Hz, 6H, CH_3) ppm. ^{13}C{H} NMR ($CDCl_3$; 75 MHz): δ 168.7 (C_{trz}–Ir), 144.8 (C_{trz}–CH_2), 81.9, 81.6 (2 x C_{COD}–H), 54.5 (N–CH_2), 51.7, 51.0 (2 x C_{COD}–H), 36.0 (N–CH_3), 34.1, 33.6, 32.2, 32.0, 30.0, 29.8, 25.2, 22.9, 20.1 (9 x CH_2), 13.9, 13.8 (2 x CH_3) ppm. HR-MS (ESI): calcd for $C_{19}H_{33}IrN_3$ [M−Cl]$^+$ m/z = 531.1992, found m/z = 531.1976. Elem. anal. found (calcd) for $C_{19}H_{33}N_3Cl_2Ir$ x 0.5 CH_2Cl_2 (566.61 g/mol): C 38.01 (38.45); H 6.06 (5.63); N 6.98 (6.90).

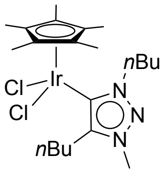

Ir(III)-trz [(Cp*)IrCl(trz)]. Following the general transmetalation procedure, using **trz-H.I** (152 mg; 0.47 mmol), Ag_2O (70 mg; 0.31 mmol) and Me_4NCl (67 mg; 0.61 mmol) in CH_2Cl_2 (3 mL) and $[Ir(Cp^*)Cl_2]_2$ (150 mg; 0.19 mmol) in CH_2Cl_2 (5 mL). The reaction mixture was stirred for 16 h. The crude product was further purified by column chromatography on SiO_2 (CH_2Cl_2 to CH_2Cl_2/acetone, 9:1), yielding **Ir2b** as a yellow powder (151 mg; 67%). An analytically pure sample was obtained by slow evaporation of Et_2O to a concentrated solution of **Ir(III)-trz** in CH_2Cl_2. ^{1}H NMR ($CDCl_3$, 300 MHz) δ 4.91–4.72, 4.32–4.13 (2 x m, 1H, CH_2–N), 3.97 (s, 3H, CH_3–N), 3.03–2.87 (m, 2H, CH_2–C_{trz}), 2.23–2.03 (m, 1H, NCH_2–CH_2), 2.00 –1.82 (m, 2H, CH_2–CH_2), 1.60 (s, 15H, Cp–CH_3), 1.52–1.37 (m, 5H, CH_2–CH_2), 0.97, 0.93 (2 x t, J = 7.1 Hz, 3H,

CH_3–CH$_2$) ppm. ^{13}C{^{1}H} NMR ($CDCl_3$, 75 MHz) δ 148.3 (C_{trz}–CH$_2$), 144.6 (*C*–Ir), 87.8 (C_{Cp}), 53.9 (N–CH$_2$), 36.5 (N–CH$_3$), 33.1, 32.3 (2 × *C*H$_2$–CH$_2$), 25.3 (*C*H$_2$–C_{trz}), 23.3, 20.4 (2 × *C*H$_2$–CH$_3$), 14.0 (*C*H$_3$–CH$_2$), 9.2 (*C*H$_3$–Cp) ppm. HR-MS (ESI): calcd for $C_{21}H_{36}ClIrN_3$ [M−Cl]$^+$ m/z = 558.2227, found m/z = 558.2214. Elem. anal. found (calcd) for $C_{21}H_{36}Cl_2IrN_3$ (593.66 g/mol): C 42.52 (42.49); H 5.92 (6.11); N 6.86 (7.08).

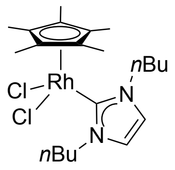

Rh(III)-imi [(Cp*)RhCl(imi)]. Following the general transmetalation procedure, using **imi-H.I** (80 mg; 0.26 mmol), Ag_2O (39 mg; 0.17 mmol) and Me_4NCl (37 mg; 0.34 mmol) in CH_2Cl_2 (15 mL) and [Cp*$RhCl_2$]$_2$ (80 mg; 0.13 mmol) in CH_2Cl_2 (5 mL). The reaction mixture was stirred for 16 h. The complex was purified by SiO_2 column chromatography using 20:1 CH_2Cl_2/acetone as eluent. Recrystallisation from CH_2Cl_2/Et_2O afforded orange-red plate shaped crystals (55 mg; 43%). ^{1}H NMR ($CDCl_3$; 300 MHz): δ 7.07 (s, 1H, H_{im}), 4.72 (td, *J* = 12.1, 5.1 Hz, 2H, N–C*H$_2$*), 3.85 (td, *J* = 12.0, 5.0 Hz, 2H, N–C*H$_2$*), 2.11–1.92 (m, 2H, C*H$_2$*–CH$_2$), 1.74–1.30 (m, 6H, C*H$_2$*–CH$_2$), 1.58 (s, 15H, CH$_3$–C_{Cp}), 0.96 (t, ^3J = 7.3 H, C*H$_3$*–CH$_2$) ppm. ^{13}C{H} NMR ($CDCl_3$; 75 MHz): δ 169.1 (d, $^1J_{Rh-C}$ = 56.8 Hz, C_{im}–Rh), 122.2 (C_{im}–H), 96.1 (d, $^1J_{Rh-C}$ = 7.0 Hz, C_{Cp}–Rh), 51.1 (N–CH$_2$), 33.9, 20.3 (2 x CH$_2$), 14.2 (*C*H$_3$–CH$_2$), 9.5 (*C*H$_3$–C_{Cp}) ppm. HR-MS (ESI): calcd for $C_{21}H_{35}N_2ClRh$ [M−Cl]$^+$ m/z = 453.1544, found m/z = 453.1528. Elem. anal. found (calcd) for $C_{21}H_{35}N_2Cl_2Rh$ x 0.1 CH_2Cl_2 (497.82 g/mol): C 51.07 (50.91); H 6.59 (7.13); N 5.51 (5.63).

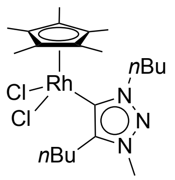

Rh(III)-trz [(Cp*)RhCl(trz)]. Following the general transmetalation procedure, using **trz-H.I** (80 mg; 0.25 mmol), Ag_2O (36 mg; 0.16 mmol) and Me_4NCl (38 mg; 0.35 mmol) in CH_2Cl_2 (5 mL) and [Cp*$RhCl_2$]$_2$ (77 mg; 0.12 mmol). The reaction mixture was stirred for 19 h. The complex was purified by SiO_2 column chromatography using 10:1 CH_2Cl_2/acetone as eluent (93 mg; 74%). ^{1}H NMR (CD_2Cl_2; 300 MHz): δ 4.92, 4.19 (2 x br m, 1H, N–C*H$_2$*), 3.99 (s, 3H, N–C*H$_3$*), 2.99, 2.83 (2 x br m, 1H, CH$_2$–C_{trz}), 2.15–1.33 (m, 8H, C*H$_2$*–CH$_2$), 1.52 (s, 15H, C*H$_3$*–C_{Cp}), 0.99 (t, ^3J = 7.4 Hz, 3H, C*H$_3$*–CH$_2$), 0.98 (t, ^3J = 7.1 Hz, 3H, C*H$_3$*–CH$_2$) ppm. ^{13}C{H} NMR (CD_2Cl_2; 75 MHz): δ 157.5 (d, $^1J_{Rh-C}$ = 52.0 Hz, C_{trz}–Rh), 147.4 (d, $^2J_{Rh-C}$, C_{trz}–CH$_2$), 95.7 (d, $^1J_{Rh-C}$ = 7.0 Hz, C_{Cp}–CH$_3$), 54.5 (N–*C*H$_2$), 36.8 (N–*C*H$_3$), 33.1, 31.7 (2 x *C*H$_2$–CH$_2$), 25.7 (*C*H$_2$–C_{trz}), 23.4, 20.5 (2 x *C*H$_2$–CH$_2$), 14.1, 14.0 (2 x *C*H$_3$–CH$_2$), 9.6 (*C*H$_3$–C_{Cp}) ppm. HR-MS (ESI): calcd for $C_{21}H_{36}N_3ClRh$ [M−Cl]$^+$ m/z = 468.1653, found m/z = 468.1639. Elem. anal. found (calcd) for $C_{21}H_{36}N_3Cl_2Rh$ (504.35 g/mol): C 50.03 (50.01); H 7.48 (7.20); N 7.95 (8.33).

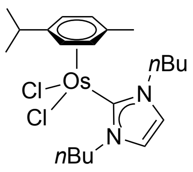

Os-imi [(*p*-cym)OsCl$_2$(imi)]. Following the general transmetalation procedure, using **imi-H.I** (57 mg; 0.18 mmol), Ag_2O (28 mg; 0.12 mmol) and Me_4NCl (26 mg; 0.24 mmol) in CH_2Cl_2 (5 mL) and [Os(*p*-cym)Cl$_2$]$_2$ (71 mg; 0.09 mmol). The reaction mixture was stirred for 14 h. The complex was purified by SiO_2 column chromatography using a gradient of CH_2Cl_2/acetone from 100:1 to 10:1, affording a yellow solid (60 mg; 58%). ^{1}H NMR ($CDCl_3$; 300 MHz): δ 6.95 (s, 2H, H_{imid}), 5.65, 5.37 (2 x d, ^3J = 5.3 Hz, 2H, H_{Ar}), 4.51, 3.97 (2 x br m, 2H, N–C*H$_2$*), 2.82 (hept, ^3J = 7.0 Hz, 1H, C*H*Me$_2$), 2.09 (s, 3H, C*H$_3$*–C_{Ar}), 1.97, 1.63 (2 x br m, 2H, C*H$_2$*–CH$_2$N), 1.52–1.31 (m, 4H, C*H$_2$*–CH$_3$), 1.24 (d, ^3J = 7.0 Hz, 6H, C*H$_3$*–CH), 0.96 (t, ^3J = 7.3 Hz, 6H, C*H$_3$*–CH$_2$) ppm. ^{13}C{H} NMR ($CDCl_3$; 75 MHz): δ 159.9 (C_{imid}–Os), 120.8 (C_{imid}–H), 99.1 (C_{Ar}–*i*Pr), 90.9 (C_{Ar}–Me), 77.4, 73.7 (2 x C_{Ar}–H), 51.5 (N–*C*H$_2$), 34.2 (*C*H$_2$–CH$_2$N), 31.0 (*C*HMe$_2$) , 23.1 (*C*H$_3$–CH), 20.4 (*C*H$_2$–CH$_3$), 18.9 (*C*H$_3$–C_{Ar}), 14.1 (*C*H$_3$–CH$_2$) ppm. HR-MS (ESI): calcd for $C_{21}H_{34}N_2ClOs$ [M−Cl]$^+$ m/z = 541.2031, found m/z = 541.1988. Elem. anal. found (calcd) for $C_{21}H_{34}N_2Cl_2Os$ x 0.5 C_3H_6O (604.69 g/mol): C 44.90 (44.69); H 6.25 (6.17); N 4.52 (4.63).

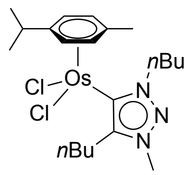

Os-trz [(*p*-cym)$OsCl_2$(trz)]. Following the general transmetalation procedure, using trz-**H.I** (59 mg; 0.18 mmol), Ag_2O (31 mg; 0.13 mmol) and Me_4NCl (30 mg; 0.27 mmol) in CH_2Cl_2 (5 mL) and [Os(*p*-cym)Cl_2]$_2$ (71 mg; 0.09 mmol). The reaction mixture was stirred for 17 h. The complex was purified by SiO_2 column chromatography using a gradient of CH_2Cl_2/acetone from 100:1 to 10:1, affording a dark yellow solid (87 mg; 82%). ^{1}H NMR ($CDCl_3$; 300 MHz): δ 5.61 (d, ^3J = 5.3 Hz, 2H, CH_{Ar}–CHMe$_2$), 5.32 (d, ^3J = 5.3 Hz, 2H, CH_{Ar}–CH$_3$), 4.55 (br m, 2H, N–CH$_2$), 3.94 (s, N–CH$_3$), 2.95 (t, ^3J = 8.4 Hz, 2H, CH$_2$–C$_{trz}$), 2.82 (hept, ^3J = 6.9 Hz, 1H, C*H*Me$_2$), 2.07 (s, 3H, CH$_3$–C$_{Ar}$), 2.04–1.89 (m, 2H, C*H$_2$*–CH$_2$N), 1.70–1.54 (m, 2H, C*H$_2$*–CH$_2$C$_{trz}$), 1.52–1.33 (m, 4H, C*H$_2$*–CH$_3$), 1.28 (d, ^3J = 6.9 Hz, 6H, C*H$_3$*–CH), 0.98 (t, ^3J = 7.2 Hz, 3H, C*H$_3$*–CH$_2$), 0.94 (t, ^3J = 6.9 Hz, 3H, C*H$_3$*–CH$_2$) ppm. ^{13}C{H} NMR ($CDCl_3$; 75 MHz): δ 148.2 (C$_{trz}$–Os), 147.4 (*C*$_{trz}$–CH$_2$), 98.0 (C$_{Ar}$–*i*Pr), 88.8 (*C*$_{Ar}$–Me), 77.4 (*C*H$_{Ar}$–C*i*Pr), 73.0 (*C*H$_{Ar}$–CMe), 54.3 (N–CH$_2$), 36.4 (N–CH$_3$), 33.4 (*C*H$_2$–CH$_2$N), 32.7 (*C*H$_2$–CH$_2$C$_{trz}$), 31.1 (*C*HMe$_2$), 26.0 (*C*H$_2$–C$_{trz}$), 23.2 (*C*H$_3$–CH), 20.4 (*C*H$_2$–CH$_3$), 18.9 (CH$_3$–C$_{Ar}$), 14.0 (*C*H$_3$–CH$_2$) ppm. HR-MS (ESI): calcd for $C_{21}H_{35}N_3ClOs$ [M−Cl]$^+$ m/z = 556.2134, found m/z = 556.2099. Elem. anal. found (calcd) for $C_{21}H_{35}N_3Cl_2Os$ x 0.5 C_3H_6O (619.70 g/mol): C 43.43 (43.61); H 6.38 (6.18); N 6.76 (6.78).

Stability assays. The complex or azolium salt (20 µmol) and mesitylene (internal standard, 3 mg) were transferred into a schlenk tube and purged with nitrogen. Extra dry 1,2-dichlorobenzene or dry dichloromethane (0.5 mL) was added and a 0.01 mL sample was taken and was diluted with 0.4 mL $CDCl_3$ or CD_2Cl_2 for NMR measurement. S_8 (12 µmol, 5 eq) was added and the reaction was started at room temperature or 120 °C. Aliquots were taken at 30 min, 2 h, 6 h and 24 h and analysed by ^{1}H NMR spectroscopy. The NCH$_2$ protons of the complexes, of the azolium salts and of the thiones were integrated towards the internal standard to calculate conversions and yields (see ESI section 3).

2. NMR spectra of the complexes

All NMR spectra measured at room temperature on Bruker spectrometers operating at 300 MHz (^1H NMR) or 75 MHz ($^{13}C\{H\}$ NMR).

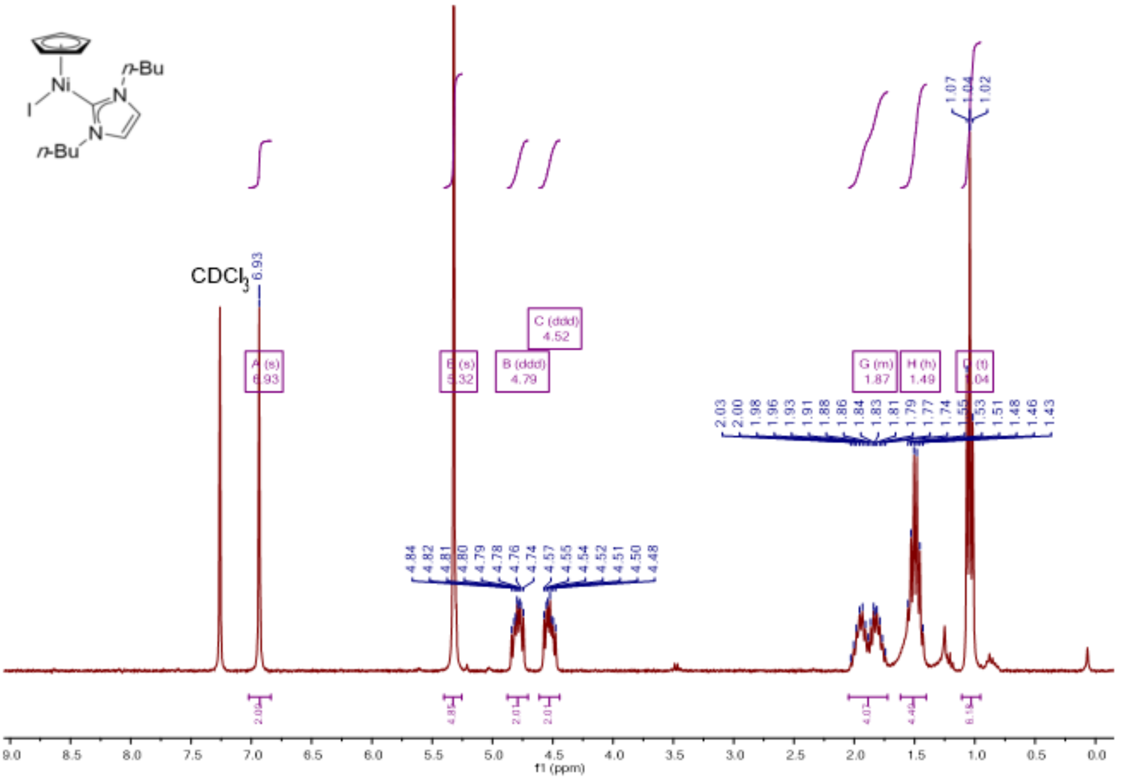

Figure S1. ^1H NMR spectrum of **Ni-imi** in $CDCl_3$.

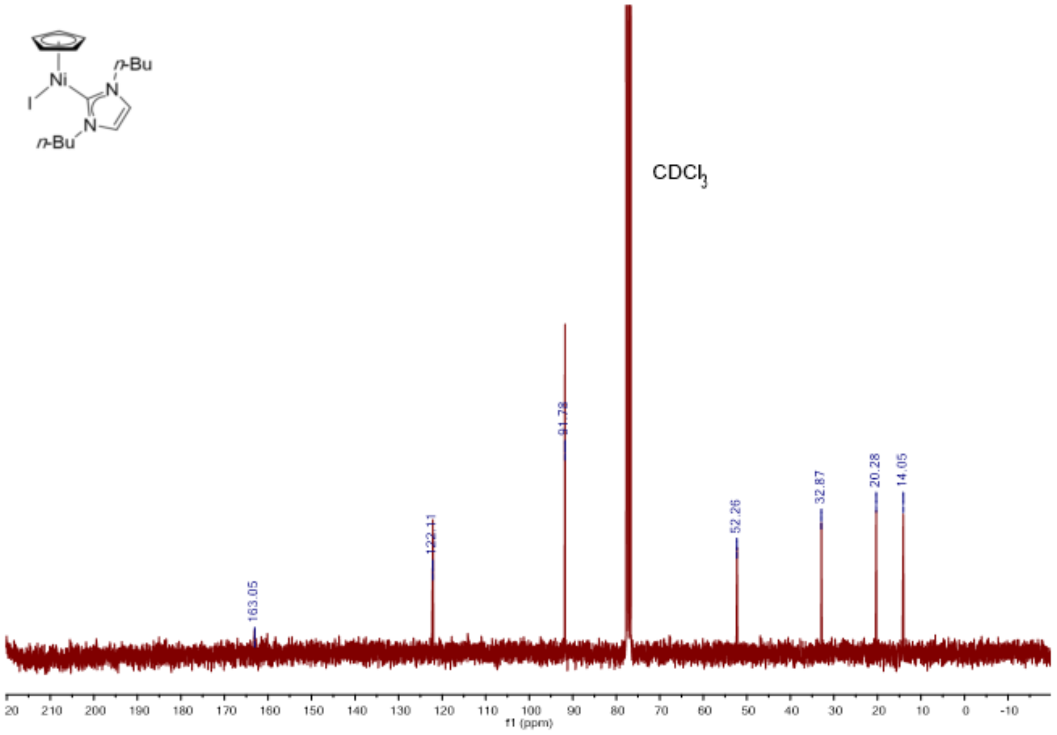

Figure S2. ^{13}C NMR spectrum of **Ni-imi** in $CDCl_3$.

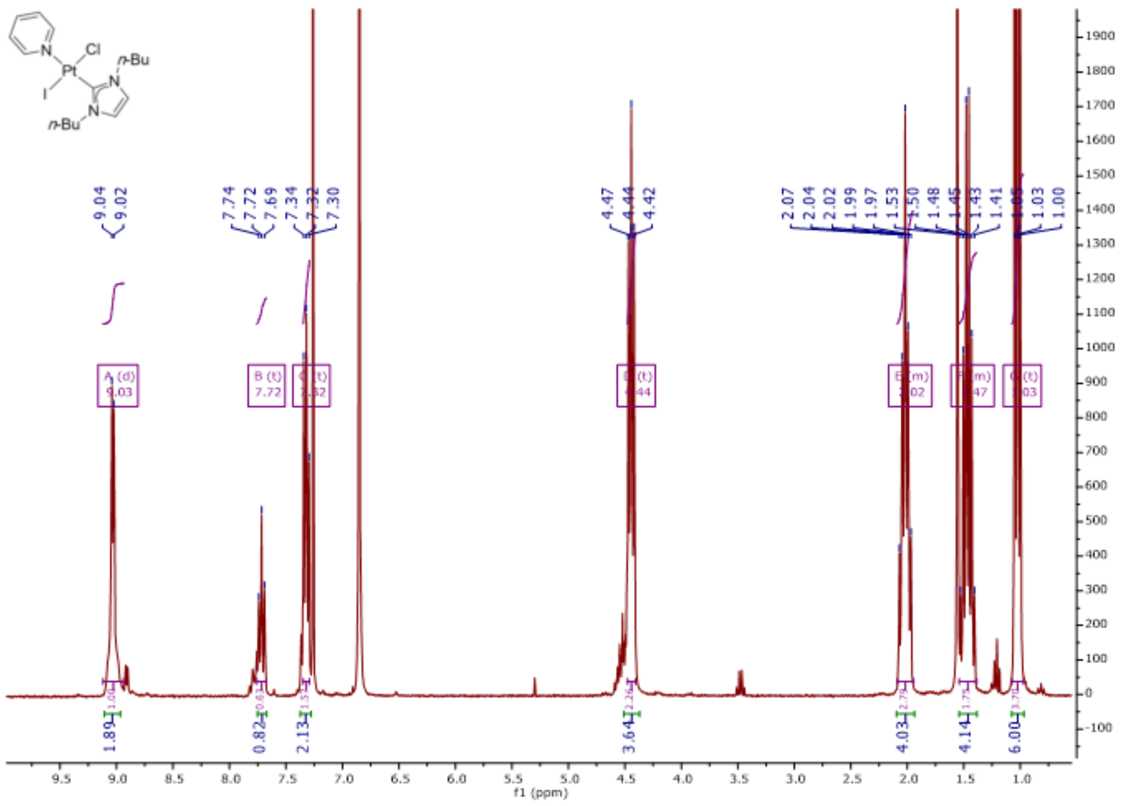

Figure S3. ^{1}H NMR spectrum of **Pt-imi** in $CDCl_3$.

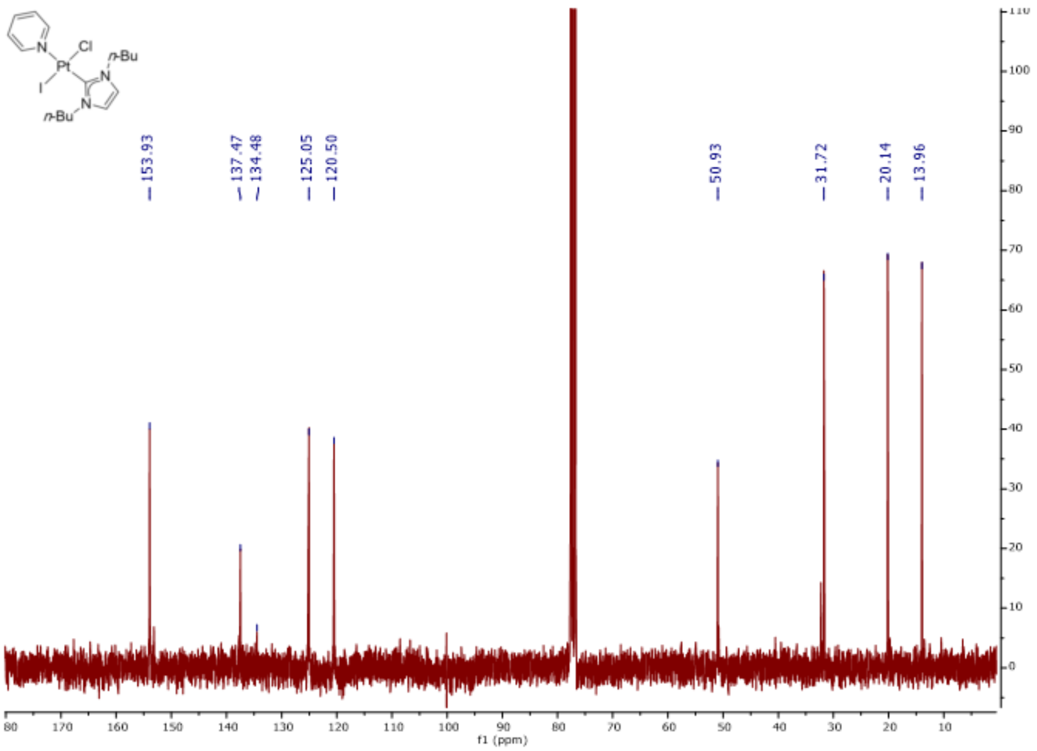

Figure S4. ^{13}C NMR spectrum of **Pt-imi** in $CDCl_3$.

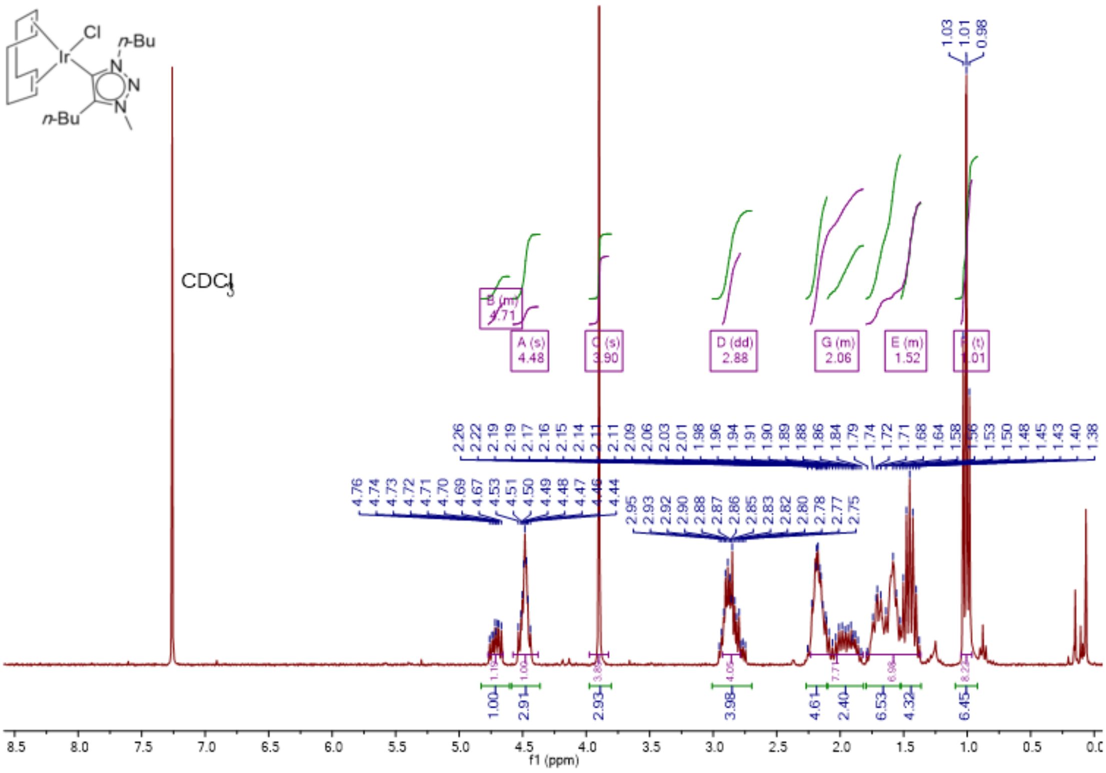

Figure S5. ^{1}H NMR spectrum of **Ir(I)-trz** in $CDCl_3$.

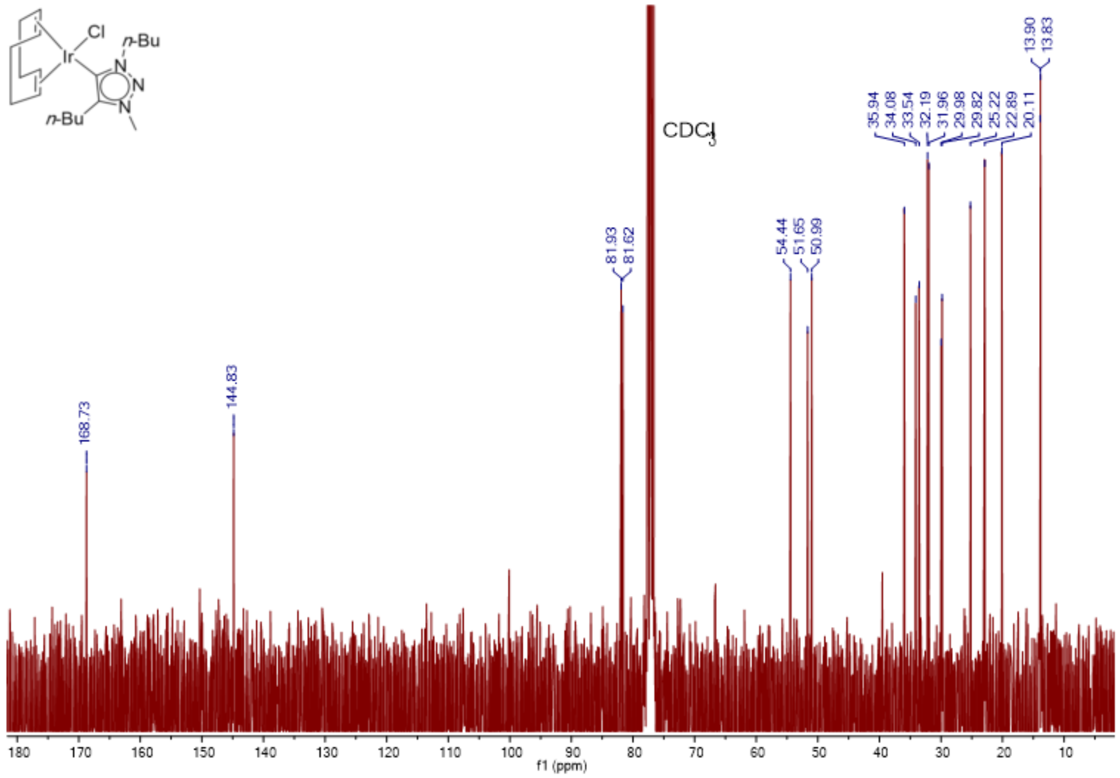

Figure S6. ^{13}C NMR spectrum of **Ir(I)-trz** in $CDCl_3$.

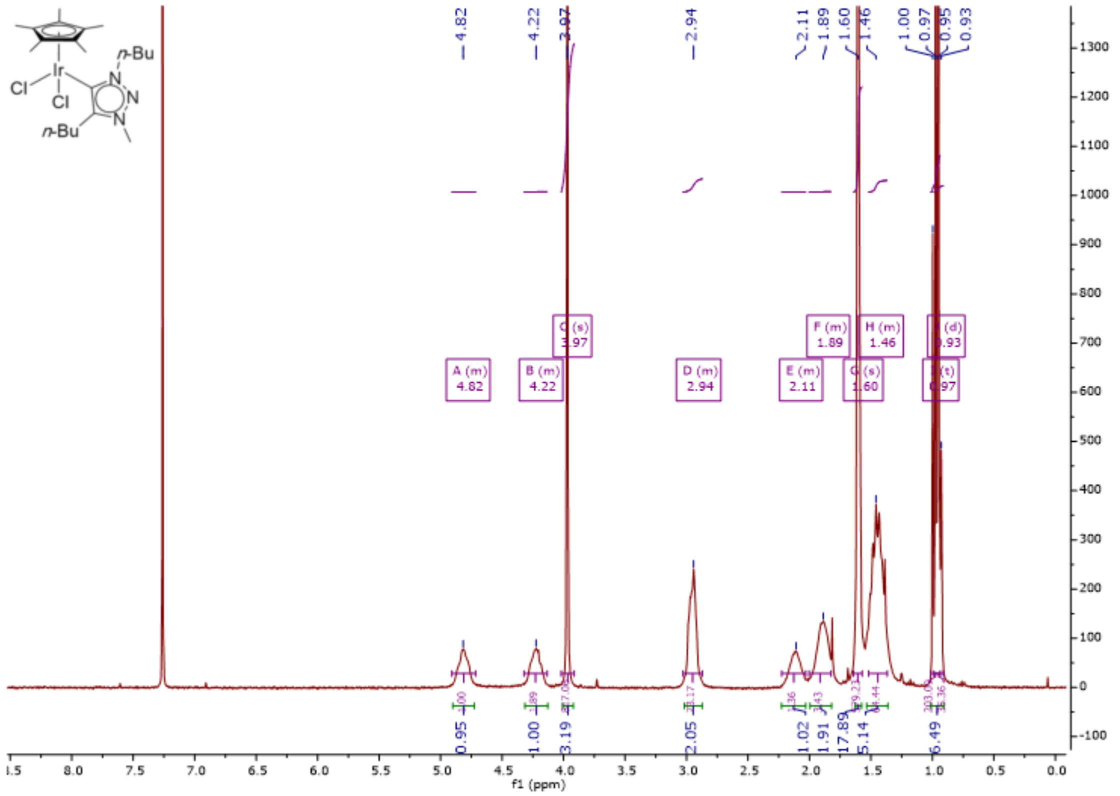

Figure S7. ^{1}H NMR spectrum of **Ir(III)-trz** in $CDCl_3$.

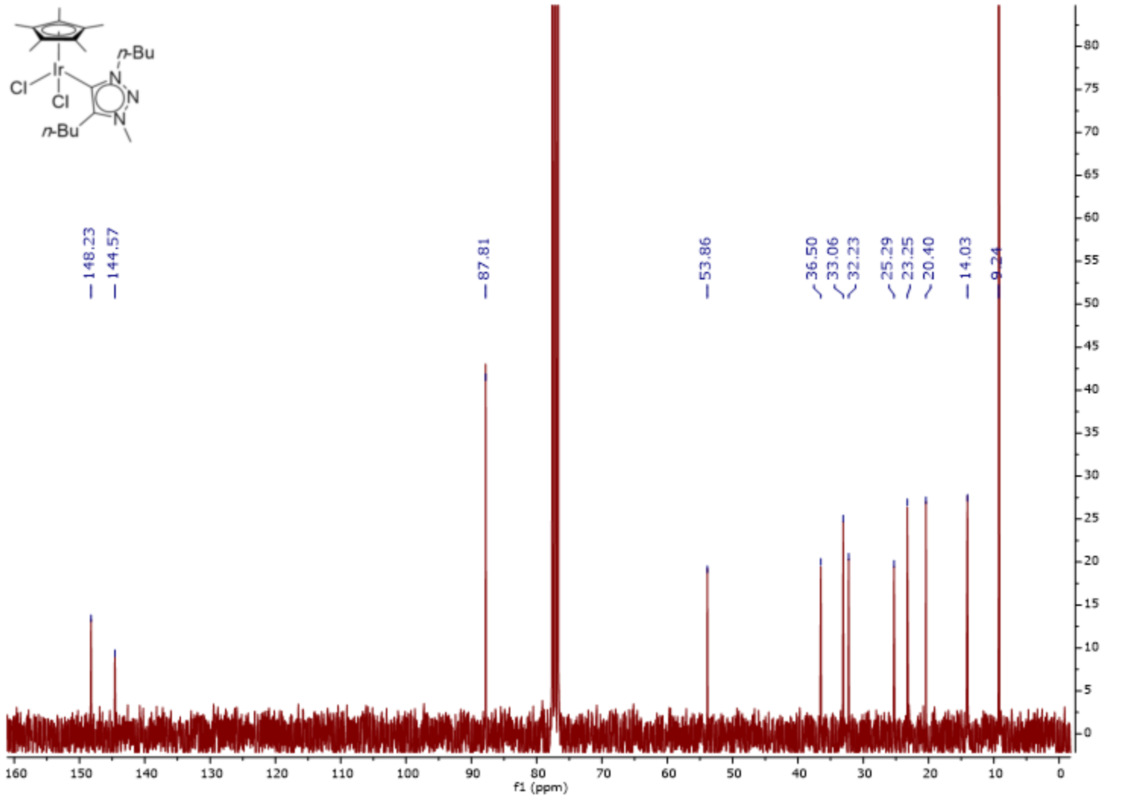

Figure S8. ^{13}C NMR spectrum of **Ir(III)-trz** in $CDCl_3$.

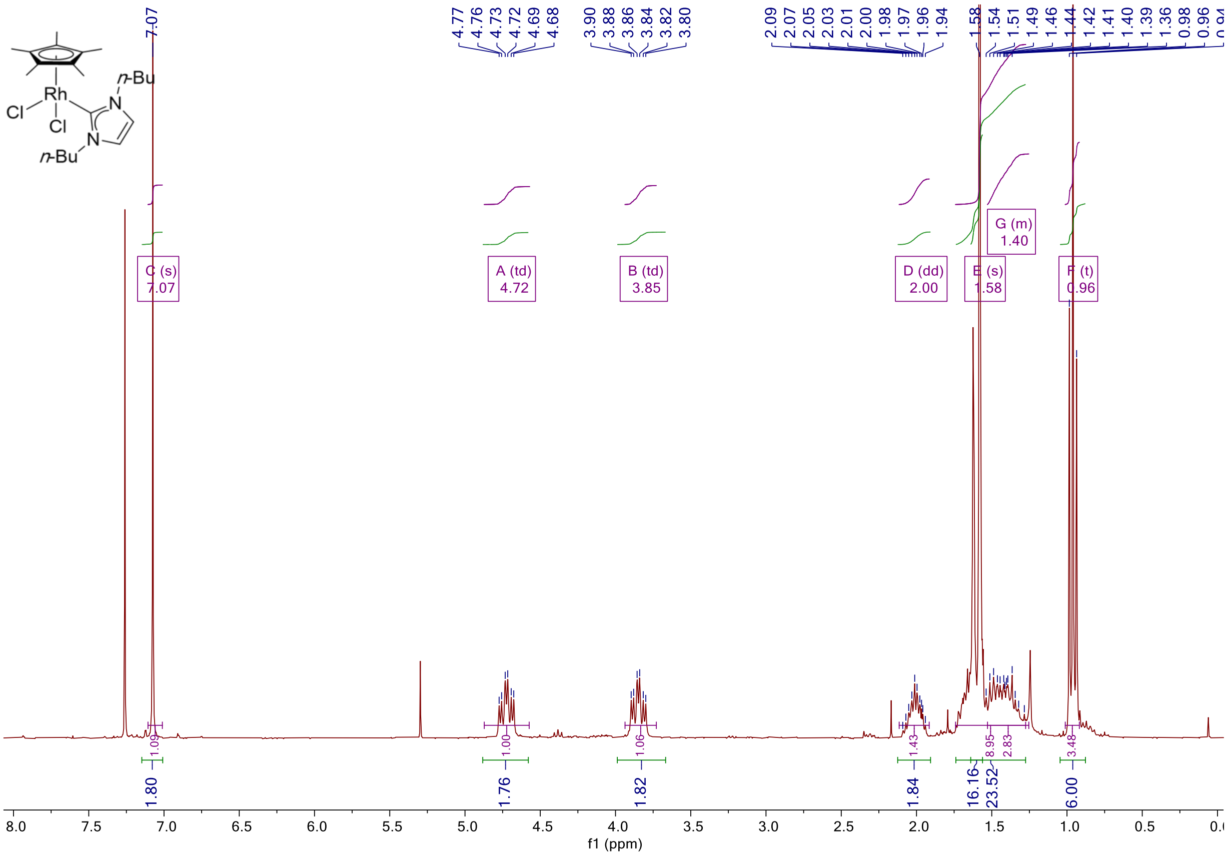

Figure S9. ^1H NMR spectrum of **Rh(III)-imi** in $CDCl_3$.

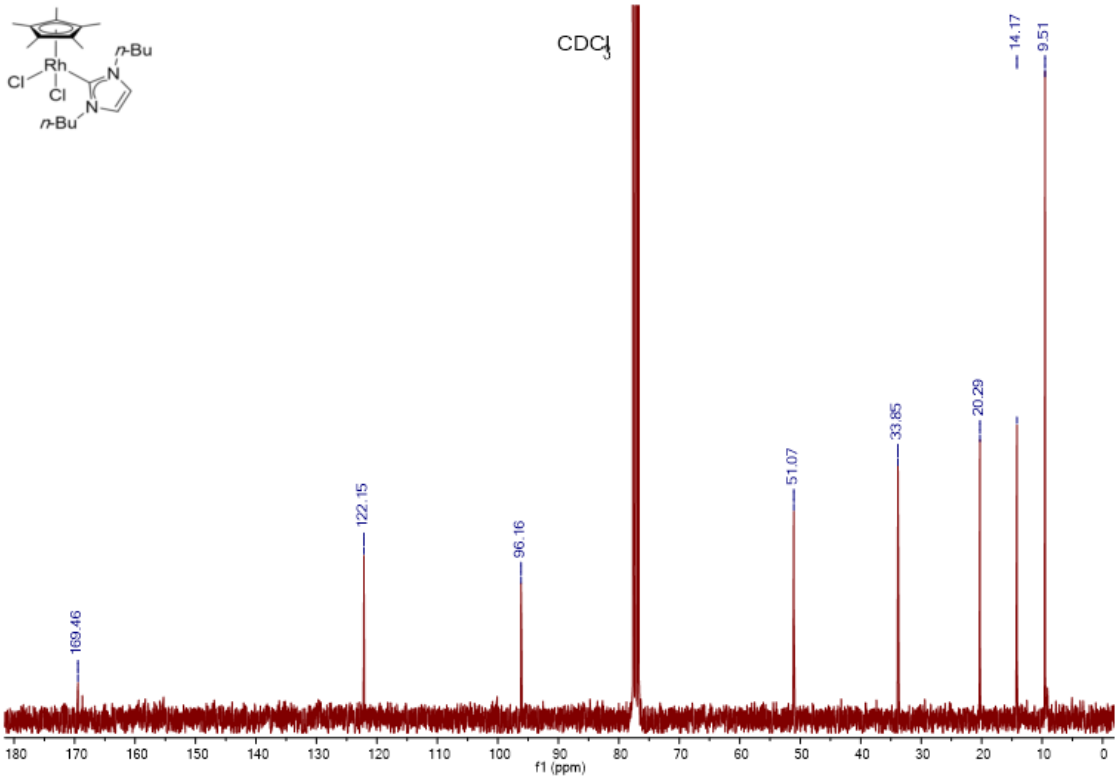

Figure S10. ^{13}C NMR spectrum of **Rh(III)-imi** in $CDCl_3$.

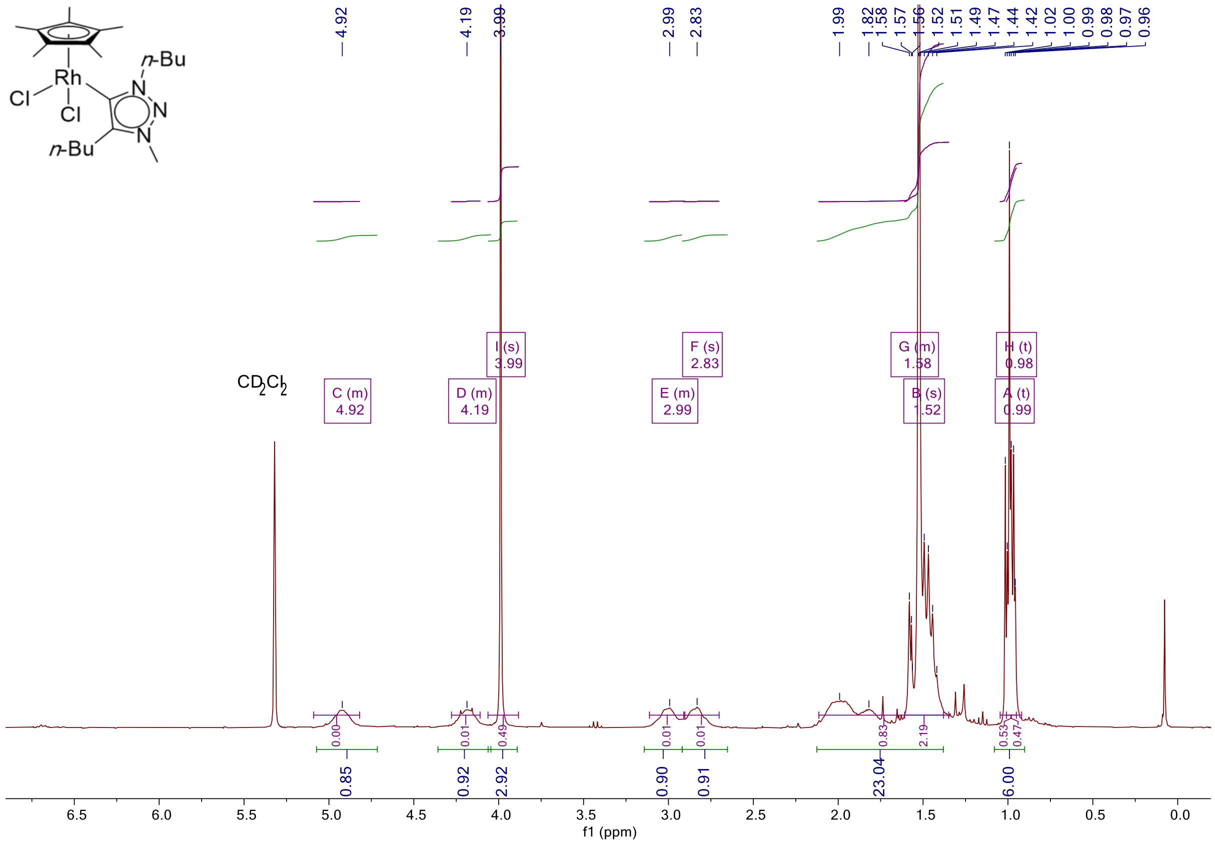

Figure S11. ^{1}H NMR spesctrum of **Rh(III)-trz** in CD_2Cl_2.

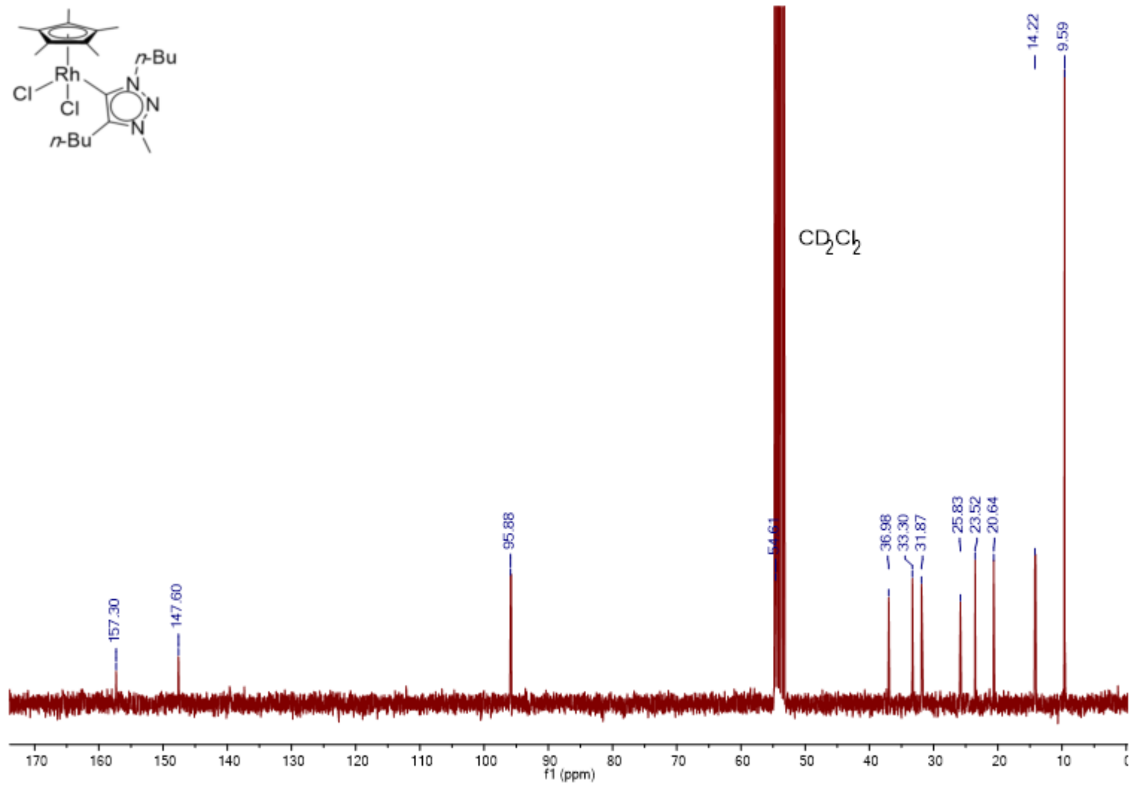

Figure S12. ^{13}C NMR spectrum of **Rh(III)-trz** in CD_2Cl_2.

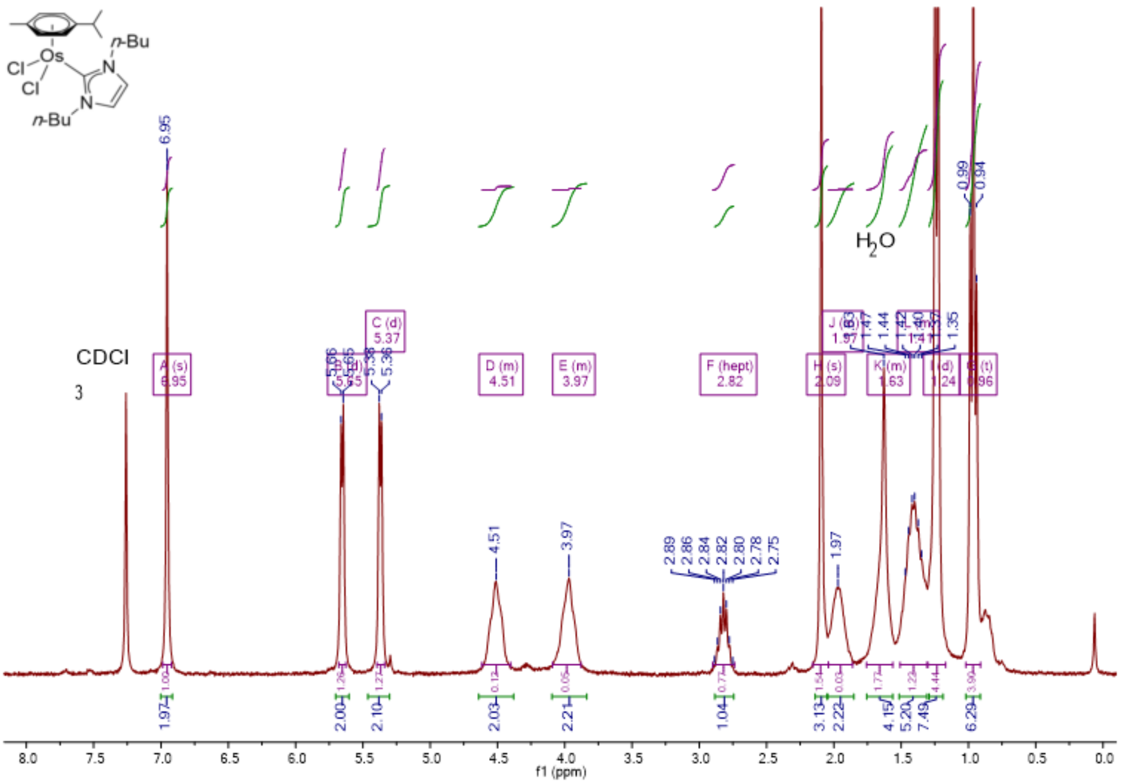

Figure S13. ^{1}H NMR spectrum of **Os-imi** in $CDCl_3$.

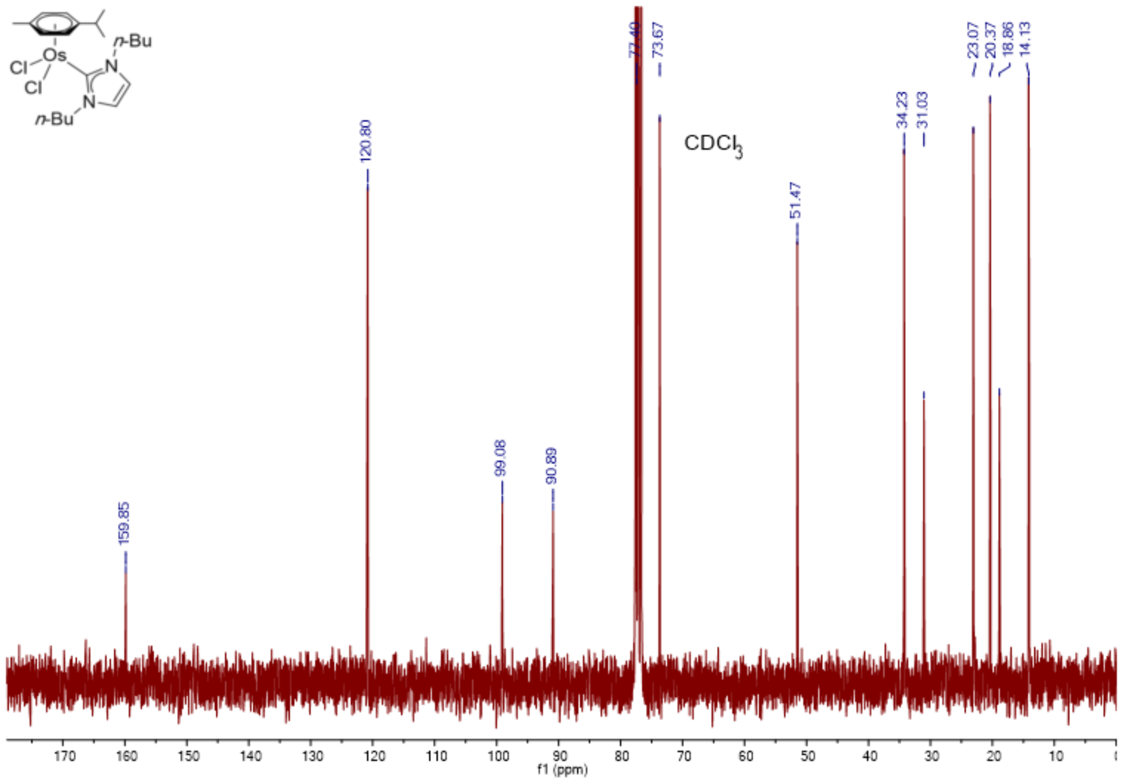

Figure S14. ^{13}C NMR spectrum of **Os-imi** in $CDCl_3$.

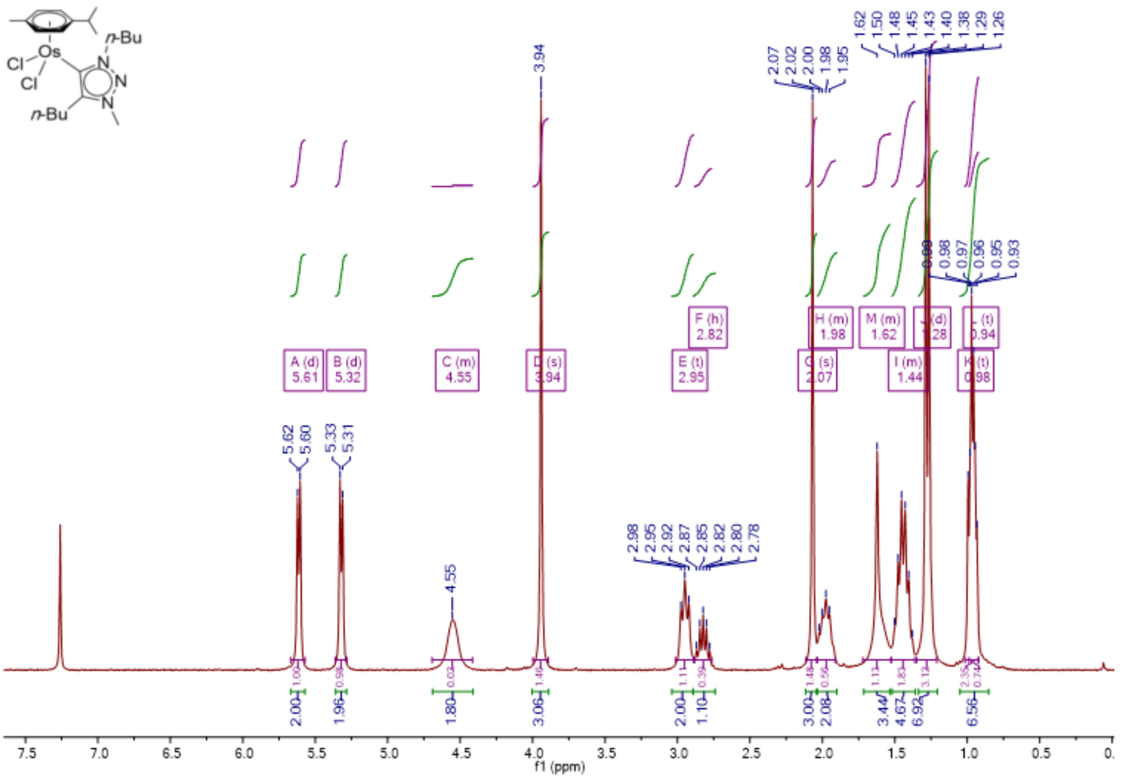

Figure S15. ^{1}H NMR spectrum of **Os-trz** in $CDCl_3$.

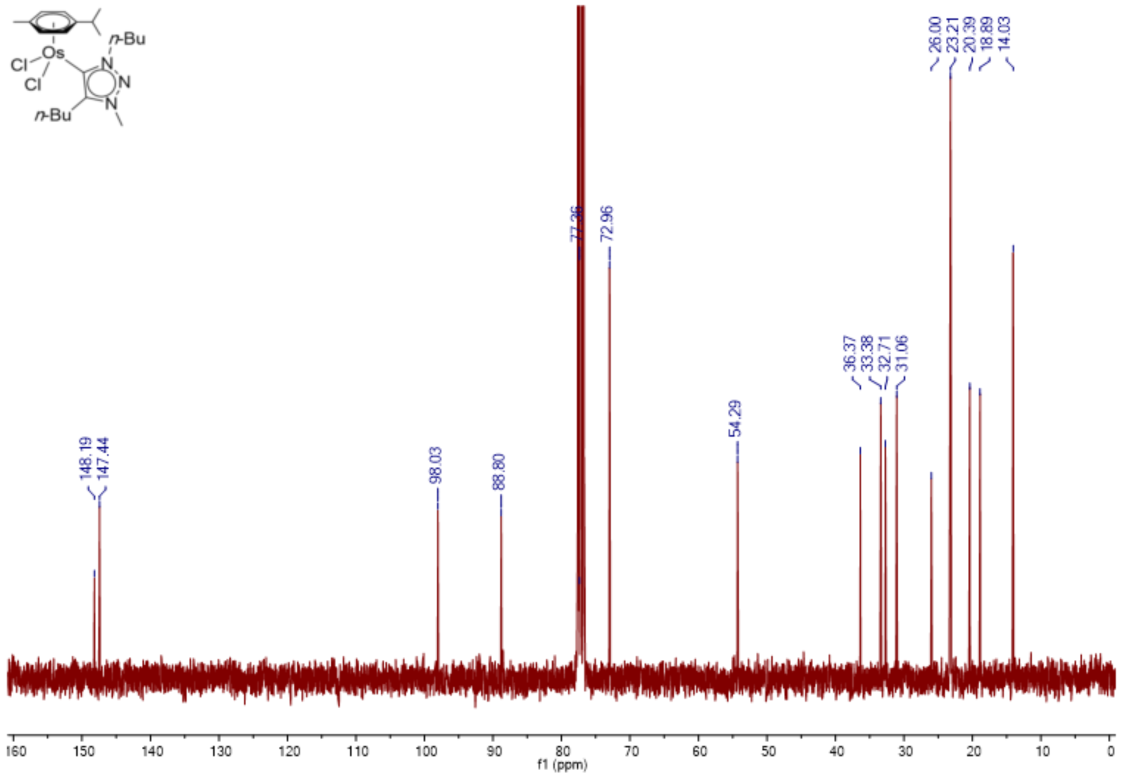

Figure S16. ^{13}C NMR spectrum of **Os-trz** in $CDCl_3$.

3. Thione formation results

Table S1. Thione formation, complex conversion over time and associated reactivity pattern at room temperature using complexes **Ag-imi** and **Ag-trz**.

Complex	% Thione/% Complex				Reactivity pattern	Figure
	30 min	2 h	6 h	24 h		
**Ag-imi**	89/11	96/4	100/0	100/0	Thione formation	S19
**Ag-trz**	55/46	94/9	98/0	98/0	Thione formation	S20

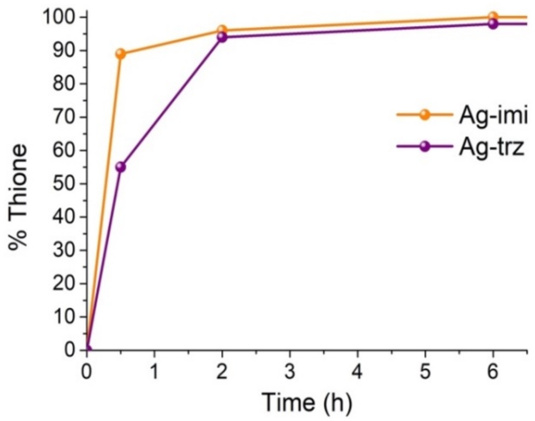

Figure S17. Thione formation over time with **Ag-imi** (orange) and **Ag-trz** (purple) measured at room temperature (20 µmol complex and 12 µmol S_8 in 0.5 mL 1,2-dichlorobenzene).

Table S2. Thione formation, complex conversion over time and associated reactivity pattern at room temperature using complexes bearing an **imi** ligand.

Complex	% Thione/% Complex				Reactivity pattern	Figure
	30 min	2 h	6 h	24 h		
**Ni-imi**	0/95	0/97	0/84	0/83	Decomposition	S24
**Ag-imi**	89/11	96/4	100/0	100/0	Thione formation	S19
**Ru-imi**	0/100	0/100	0/100	0/94	Ancillary ligand dissociation	S21
**Rh(I)-imi**	0/97	0/93	0/81	0/59	Decomposition	S23
**Os-imi**	0/100	0/100	0/93	0/91	Decomposition	S25
**Ir(I)-imi**	0/96	0/93	0/85	0/32	Ancillary ligand dissociation	S22

Table S3. Thione formation, complex conversion over time and associated reactivity pattern at 120 °C, using complexes bearing an **imi** ligand.

Complex	% Thione/% Complex (/% Salt)				Reactivity pattern	Figure
	30 min	2 h	6 h	24 h		
**imi-H.I**	7/93	8/90	13/87	30/68	-	S43
**Ni-imi**	38/0/34	38/0/73	38/0/64	42/0/69	NHC dissociation	S42
**Au-imi**	0/100	0/100	0/100	0/100	No modification	S33
**Ru-imi**	0/86	0/51	18/14	24/0	ligand dissociation + thione	S37
**Rh(I)-imi**	5/22	10/9	15/2	15/0	ligand dissociation + thione	S38
**Rh(III)-imi**	13/52/26	15/0/66	32/0/64	37/0/61	NHC dissociation	S41
**Pd-imi**	0/100	0/100	0/97	0/93	Decomposition	S34
**Os-imi**	0/87	3/84	5/71	8/45	Decomposition + thione	S39
**Ir(I)-imi**	0/29	0/9	0/0	0/0	Decomposition	S36
**Ir(III)-imi**	0/93/0	0/92/5	1/73/20	2/6/38	NHC dissociation	S40
**Pt-imi**	0/100	0/96	0/89	0/69	Decomposition	S35

Table S4. Thione formation, complex conversion over time and associated reactivity pattern at 120 °C, using complexes bearing an **IMes** ligand.

Complex	% Thione/% Complex (/% Salt)				Reactivity pattern	Figure
	30 min	2 h	6 h	24 h		
**Ag-IMes**	99/0	99/0	99/0	99/0	thione	S53
**Ni-IMes**	28/0/18	40/0/10	69/0/0	99/0/0	ligand & NHC dissociation	S54
**Rh(I)-IMes**	0/0	5/0	19/0	42/0	ligand dissociation + thione	S55
**Ir(I)-IMes**	0/50	0/0	0/0	0/0	degradation	S56

Table S5. Thione formation, complex conversion over time and associated reactivity pattern at 25 °C using **trz** complexes.

Complex	% Thione/% Complex				Reactivity pattern	Figure
	30 min	2 h	6 h	24 h		
**Ag-trz**	55/46	94/9	98/0	98/0	Thione formation	S20
**Ru-trz**	0/100	0/100	0/100	0/92	Ancillary ligand dissociation	S29
**Rh(I)-trz**	0/77	0/48	0/30	0/0	Decomposition	S31
**Rh(III)-trz**	0/100	0/100	0/100	0/100	No modification	S26
**Pd-trz**	0/100	0/100	0/100	0/100	No modification	S27
**Os-trz**	0/100	0/98	0/98	0/92	Decomposition	S32
**Ir(I)-trz**	0/83	0/49	0/0	0/0	Ancillary ligand dissociation	S30
**Ir(III)-trz**	0/100	0/100	-	0/100	No modification	S28

Table S6. Thione formation, conversion over time and associated reactivity pattern at 120 °C, using **trz** complexes.

Complex	% Thione/% Complex/% Salt				Reactivity pattern	Figure
	30 min	2 h	6 h	24 h		
**trz-H.I**	13/85	41/55	63/31	71/25	-	S52
**Au-trz**	0/100	0/100	0/100	0/100	No modification	S44
**Ru-trz**	0/78	0/33	24/2	50/1	ligand dissociation + thione	S47
**Rh(I)-trz**	0/0	0/0	18/0	22/0	ligand dissociation + thione	S48
**Rh(III)-trz**	0/62/19	23/0/72	87/0/12	100/0/0	NHC dissociation	S51
**Pd-trz**	0/98	0/99	0/95	0/89	Decomposition	S45
**Os-trz**	0/80	0/72	6/53	28/4	Decomposition + thione	S49
**Ir(I)-trz**	0/0	0/0	0/0	0/0	ligand dissociation	S46
**Ir(III)-trz**	0/100	0/98	7/92	57/38	Decomposition + thione	S50

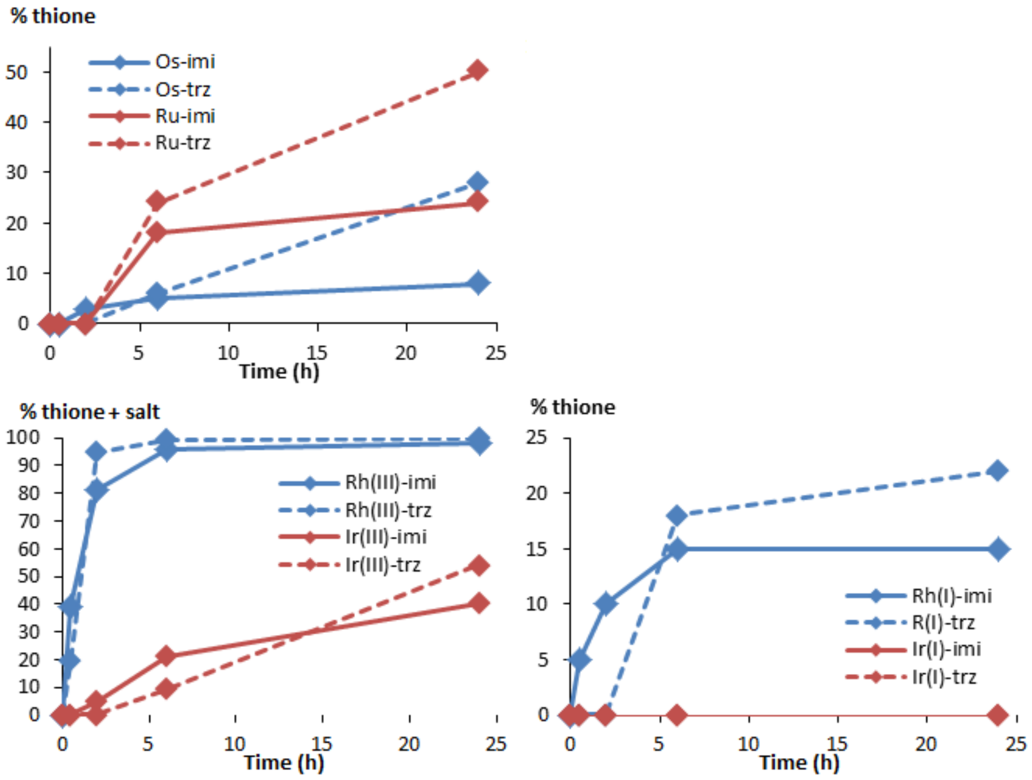

Figure S18. Conversion profile of selected complexes into thione and salt, over 24 h. Plain line for imi complexes and dashed lines for trz complexes. Top left, group 8/d^6 metals; bottom left, group 9/d^6 metals; bottom right, group 9/d^8 metals.

4. NMR spectra of the stability tests at room temperature

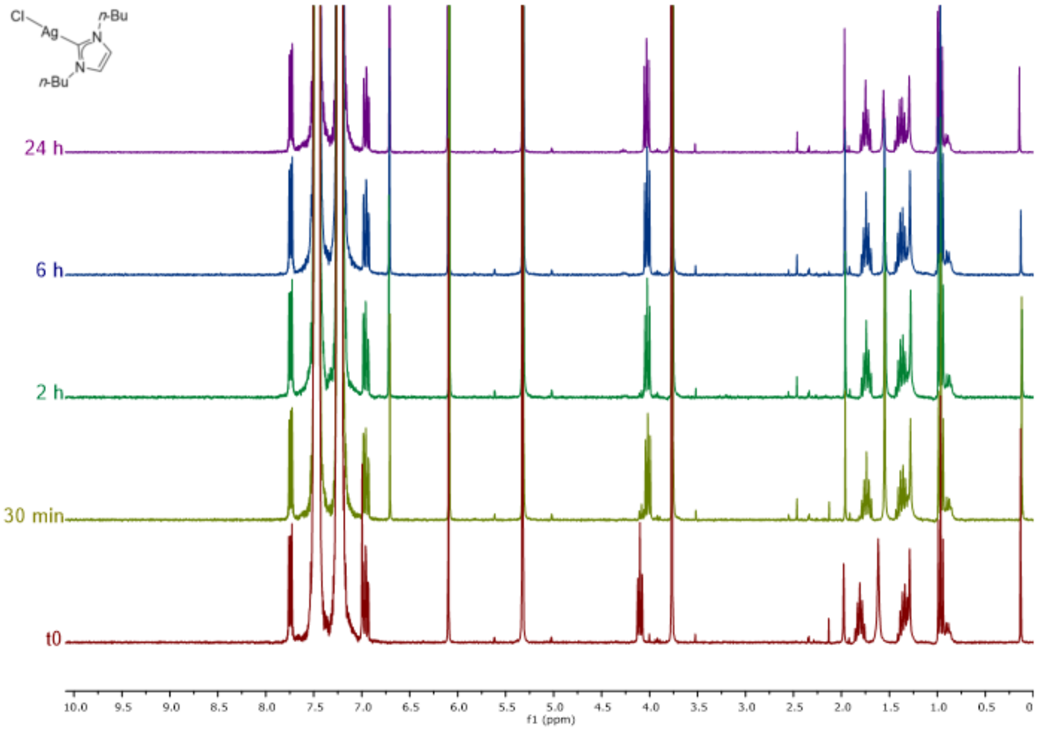

Figure S19. ^{1}H NMR spectra of samples (0, 30 min, 2, 6 and 24 h) from the reaction between **Ag-imi** and S_8 in dichlorobenzene at room temperature, measured in CD_2Cl_2.

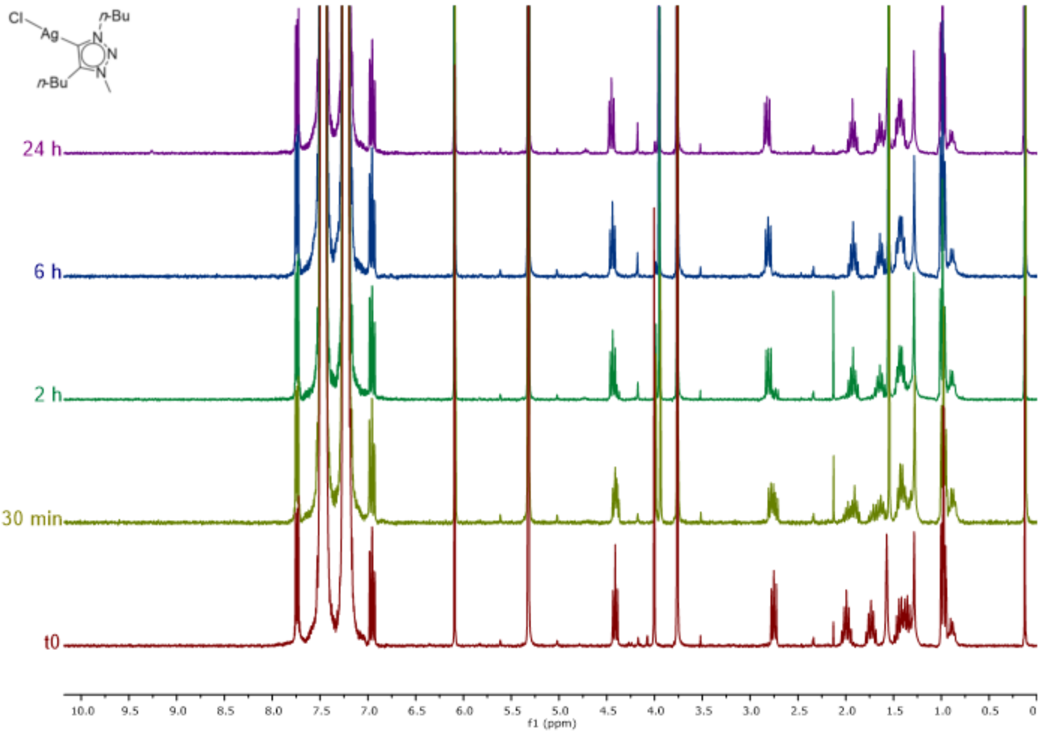

Figure S20. ^{1}H NMR spectra of samples (0, 30 min, 2, 6 and 24 h) from the reaction between **Ag-trz** and S_8 in dichlorobenzene at room temperature, measured in CD_2Cl_2.

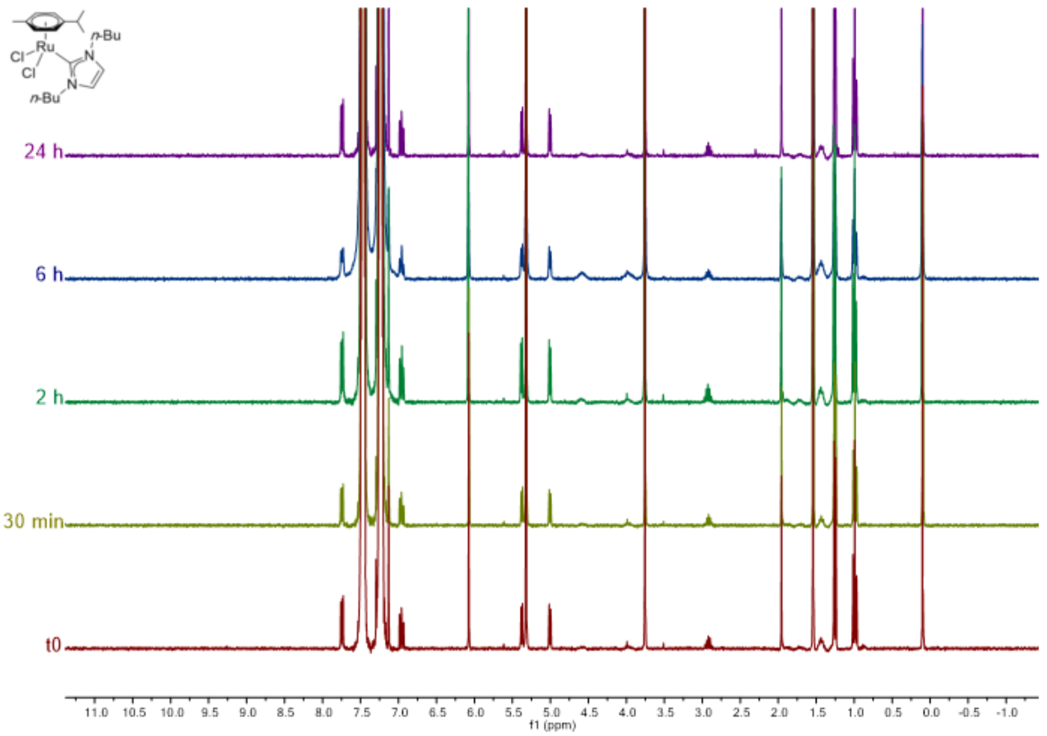

Figure S21. ^{1}H NMR spectra of samples (0, 30 min, 2, 6 and 24 h) from the reaction between **Ru-imi** and S_8 in dichlorobenzene at room temperature, measured in CD_2Cl_2.

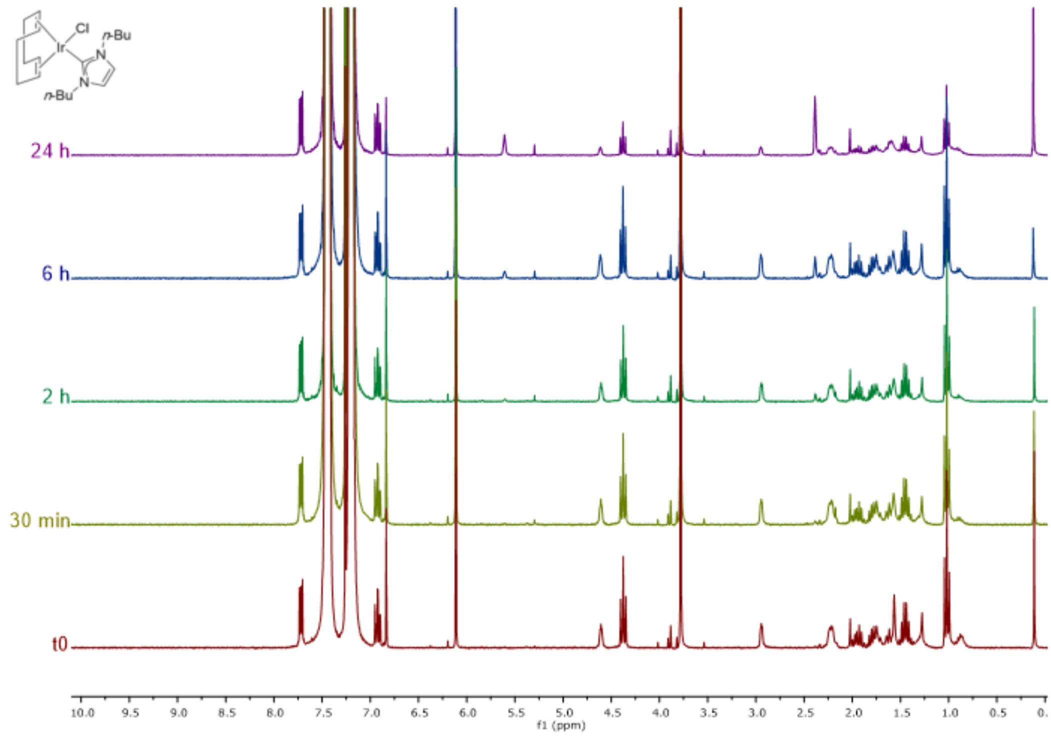

Figure S22. ^{1}H NMR spectra of samples (0, 30 min, 2, 6 and 24 h) from the reaction between **Ir(I)-imi** and S_8 in dichlorobenzene at room temperature, measured in $CDCl_3$.

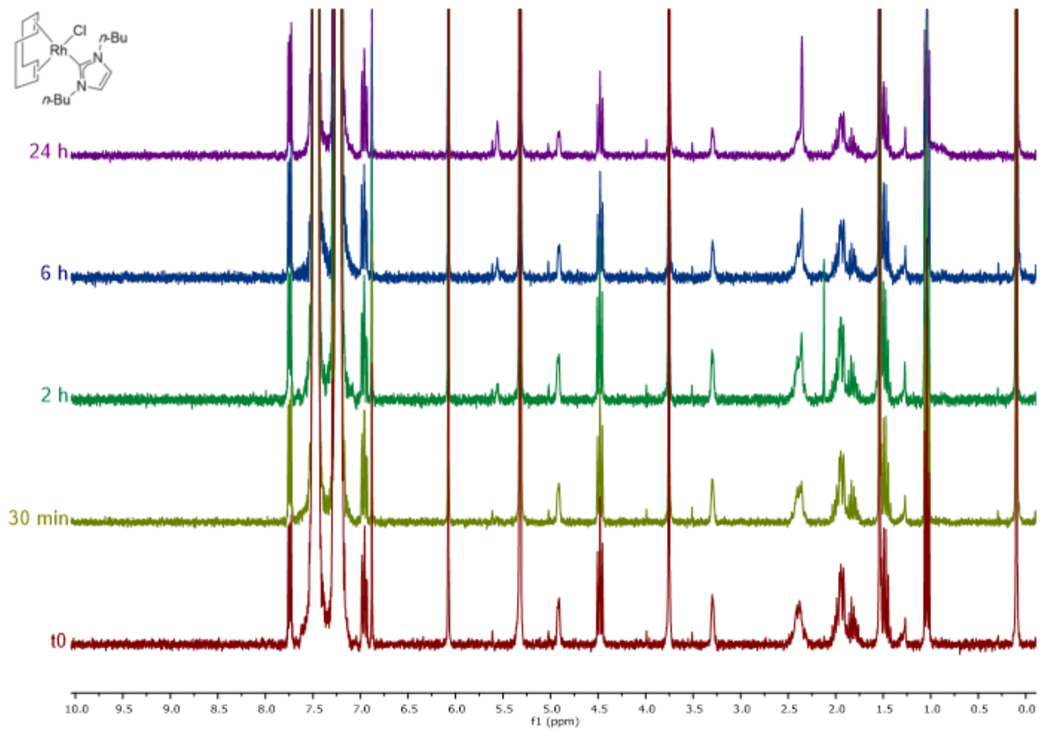

Figure S23. ^{1}H NMR spectra of samples (0, 30 min, 2, 6 and 24 h) from the reaction between **Rh(I)-imi** and S_8 in dichlorobenzene at room temperature, measured in CD_2Cl_2.

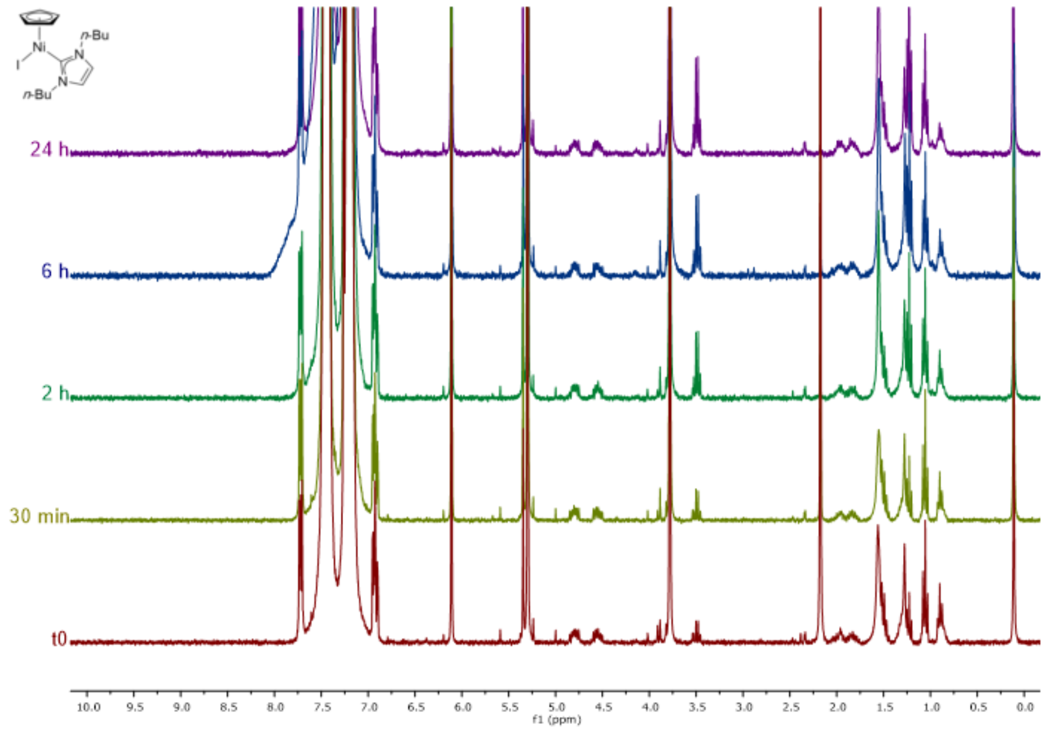

Figure S24. ^{1}H NMR spectra of samples (0, 30 min, 2, 6 and 24 h) from the reaction between **Ni-imi** and S_8 in dichlorobenzene at room temperature, measured in CD_2Cl_2.

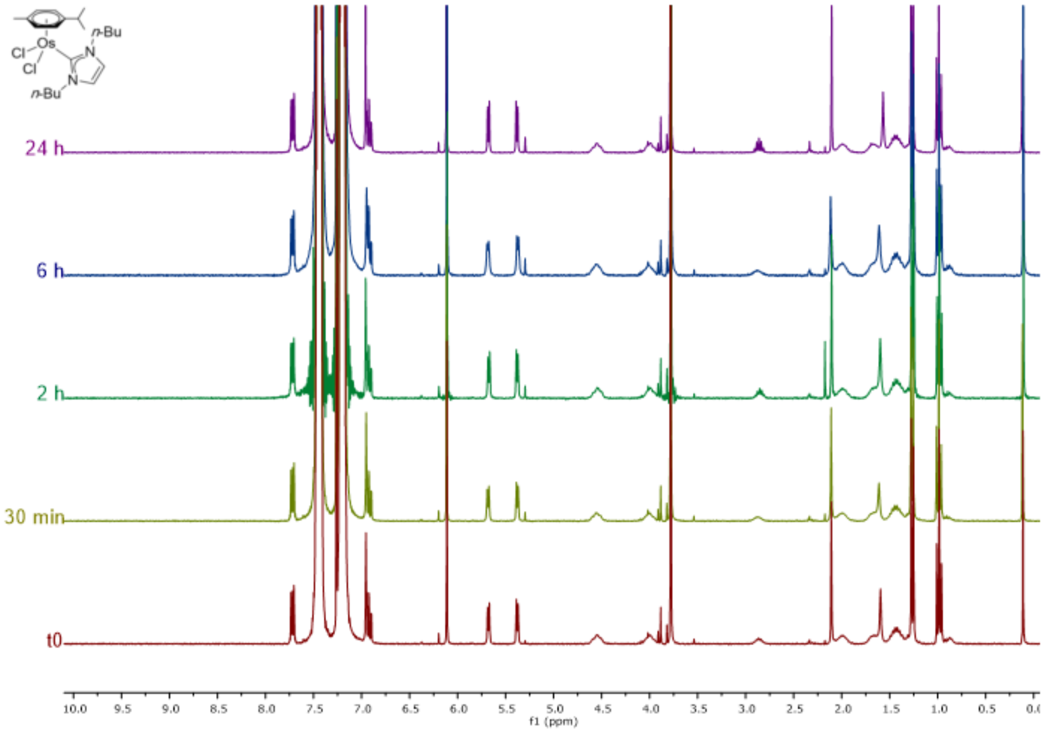

Figure S25. ^{1}H NMR spectra of samples (0, 30 min, 2, 6 and 24 h) from the reaction between **Os-imi** and S_8 in dichlorobenzene at room temperature, measured in $CDCl_3$.

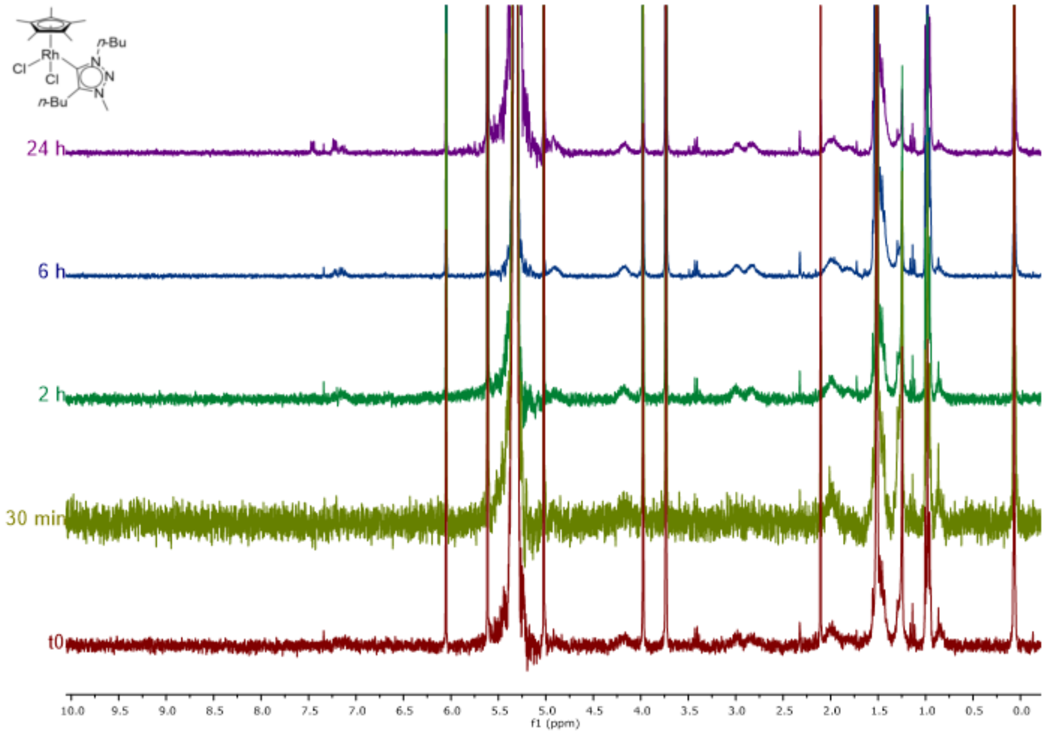

Figure S26. ^{1}H NMR spectra of samples (0, 30 min, 2, 6 and 24 h) from the reaction between **Rh(III)-trz** and S_8 in dichloromethane at room temperature, measured in CD_2Cl_2.

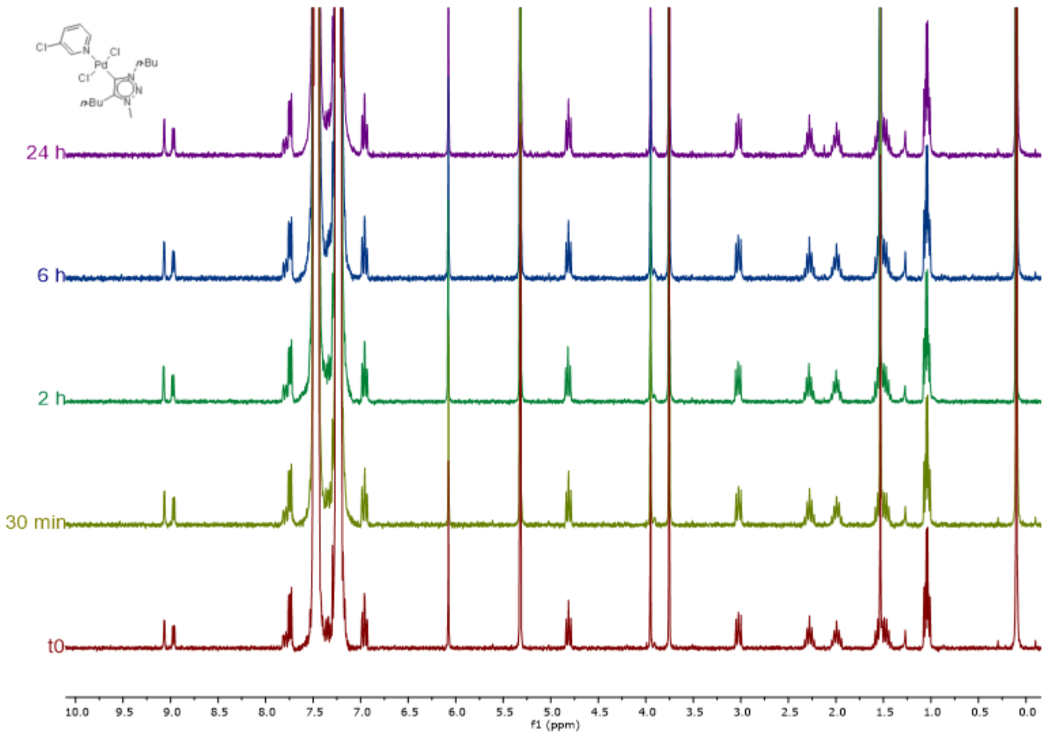

Figure S27. ^{1}H NMR spectra of samples (0, 30 min, 2, 6 and 24 h) from the reaction between **Pd-trz** and S_8 in dichlorobenzene at room temperature, measured in CD_2Cl_2.

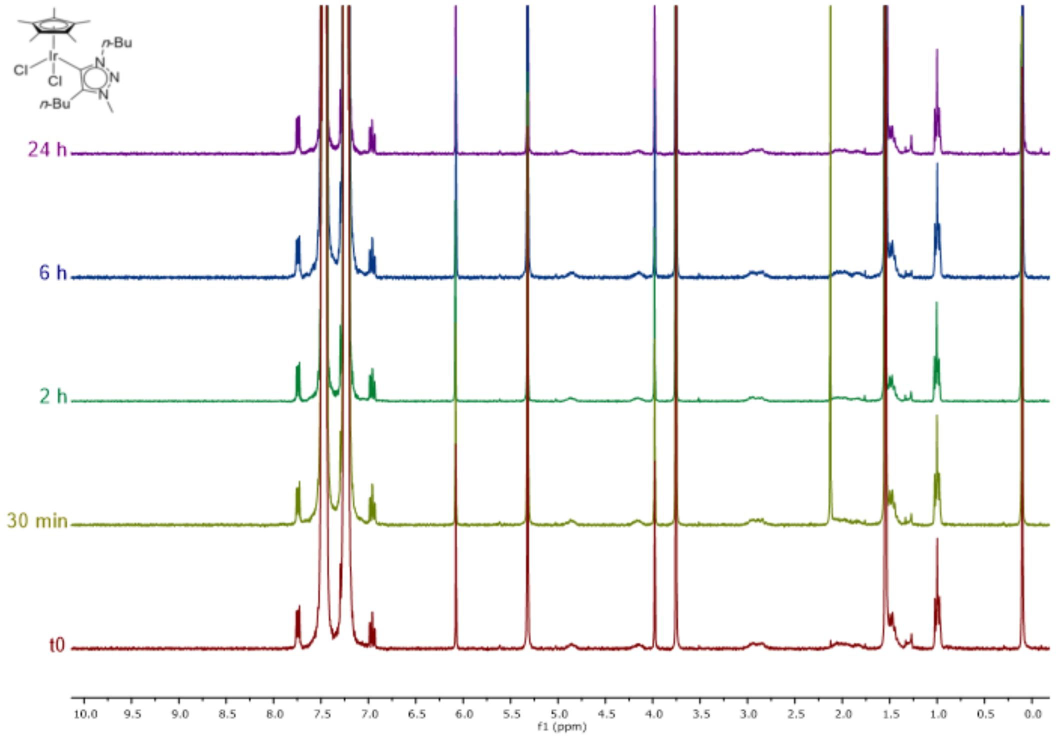

Figure S28. ^{1}H NMR spectra of samples (0, 30 min, 2, 6 and 24 h) from the reaction between **Ir(III)-trz** and S_8 in dichlorobenzene at room temperature, measured in CD_2Cl_2.

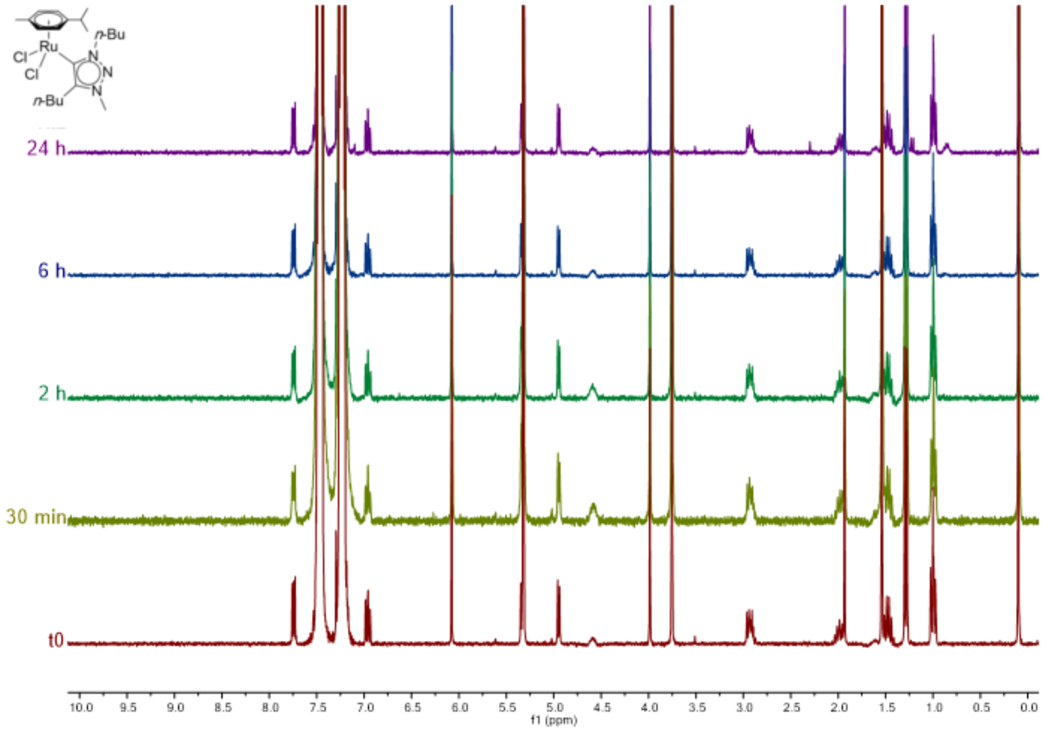

Figure S29. ^{1}H NMR spectra of samples (0, 30 min, 2, 6 and 24 h) from the reaction between **Ru-trz** and S_8 in dichlorobenzene at room temperature, measured in CD_2Cl_2.

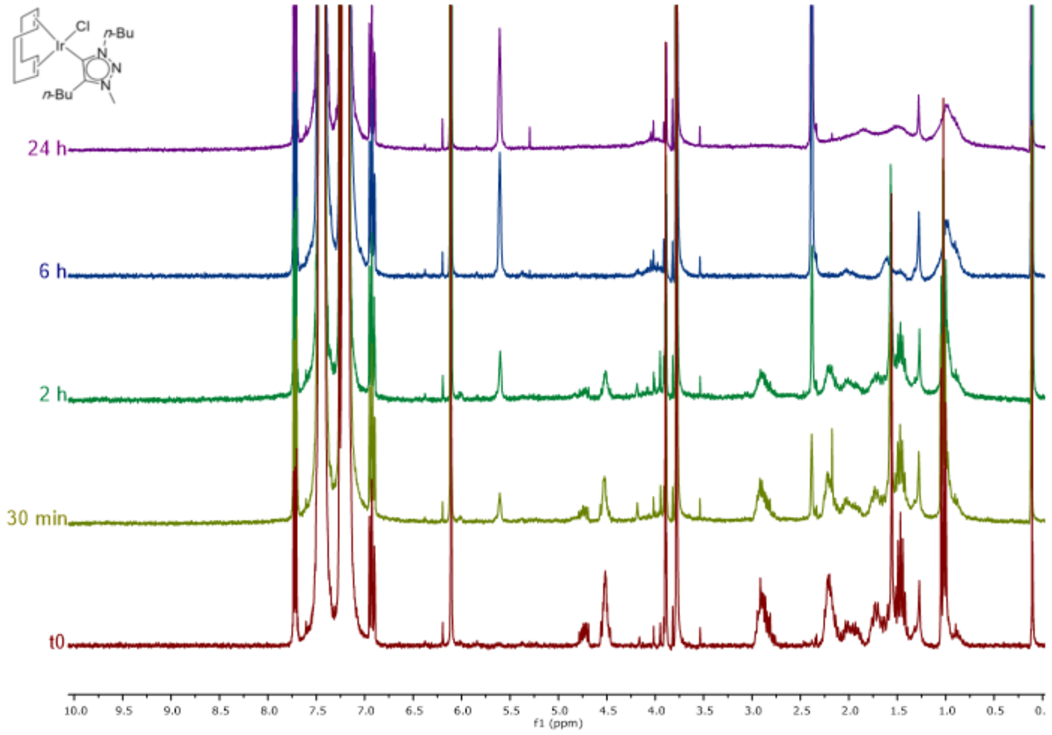

Figure S30. ^{1}H NMR spectra of samples (0, 30 min, 2, 6 and 24 h) from the reaction between **Ir(I)-trz** and S_8 in dichlorobenzene at room temperature, measured in $CDCl_3$.

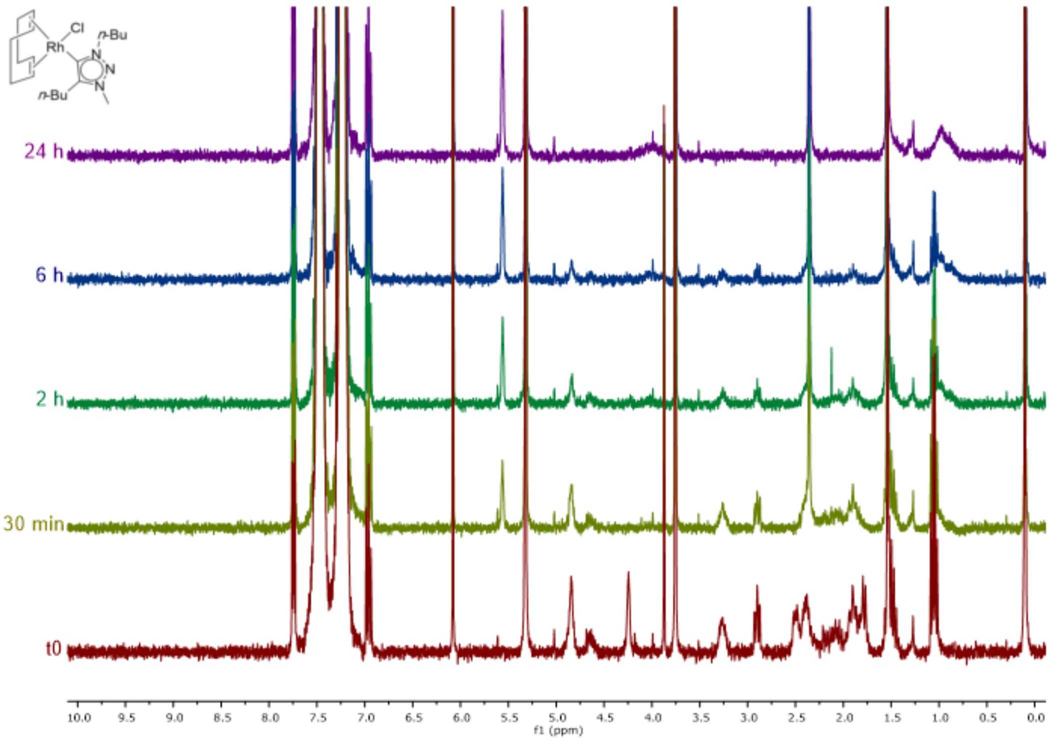

Figure S31. ^{1}H NMR spectra of samples (0, 30 min, 2, 6 and 24 h) from the reaction between **Rh(I)-trz** and S_8 in dichlorobenzene at room temperature, measured in CD_2Cl_2.

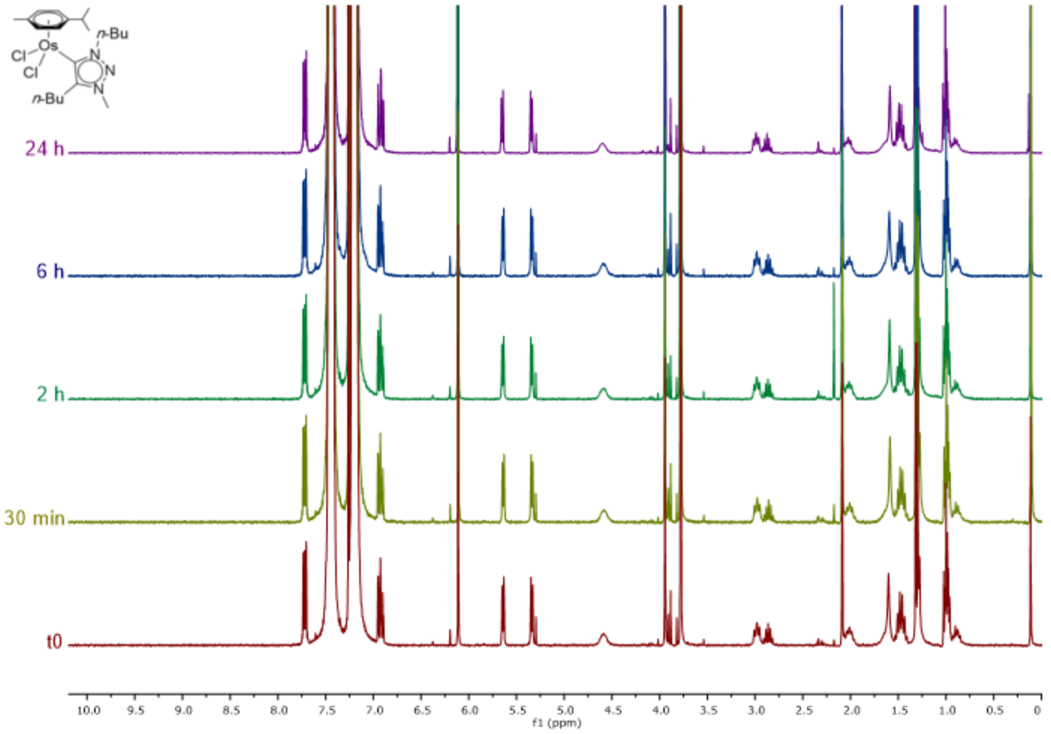

Figure S32. ^{1}H NMR spectra of samples (0, 30 min, 2, 6 and 24 h) from the reaction between **Os-trz** and S_8 in dichlorobenzene at room temperature, measured in $CDCl_3$.

5. NMR spectra of the stability tests at 120 °C

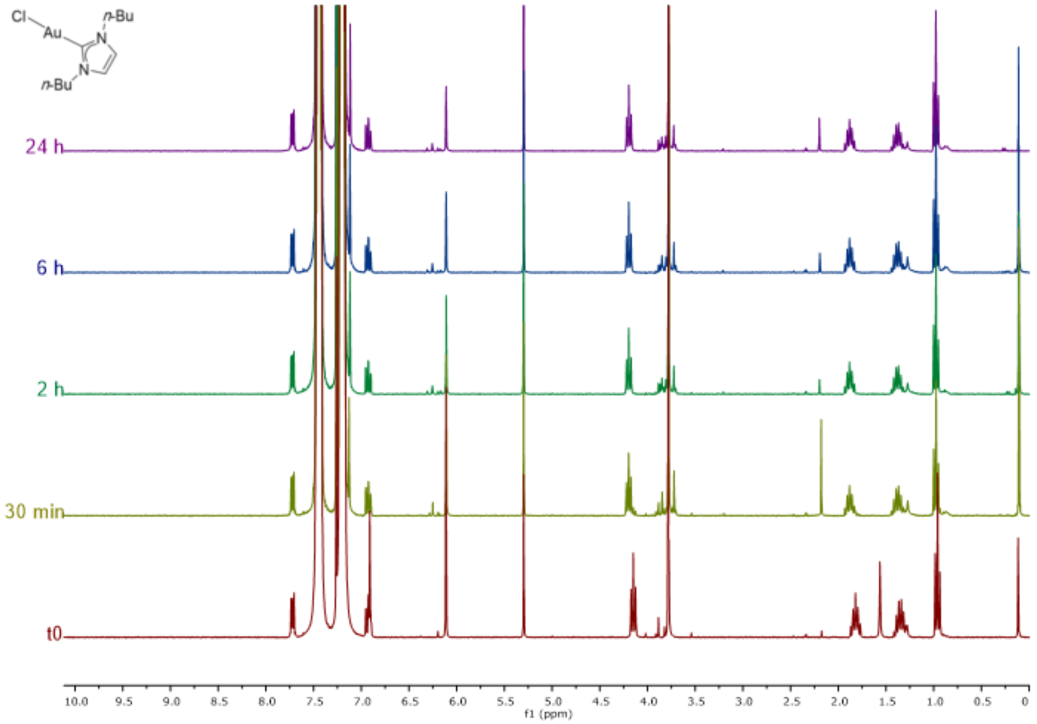

Figure S33. ^{1}H NMR spectra of samples (0, 30 min, 2, 6 and 24 h) from the reaction between **Au-imi** and S_8 in dichlorobenzene at room temperature, measured in $CDCl_3$.

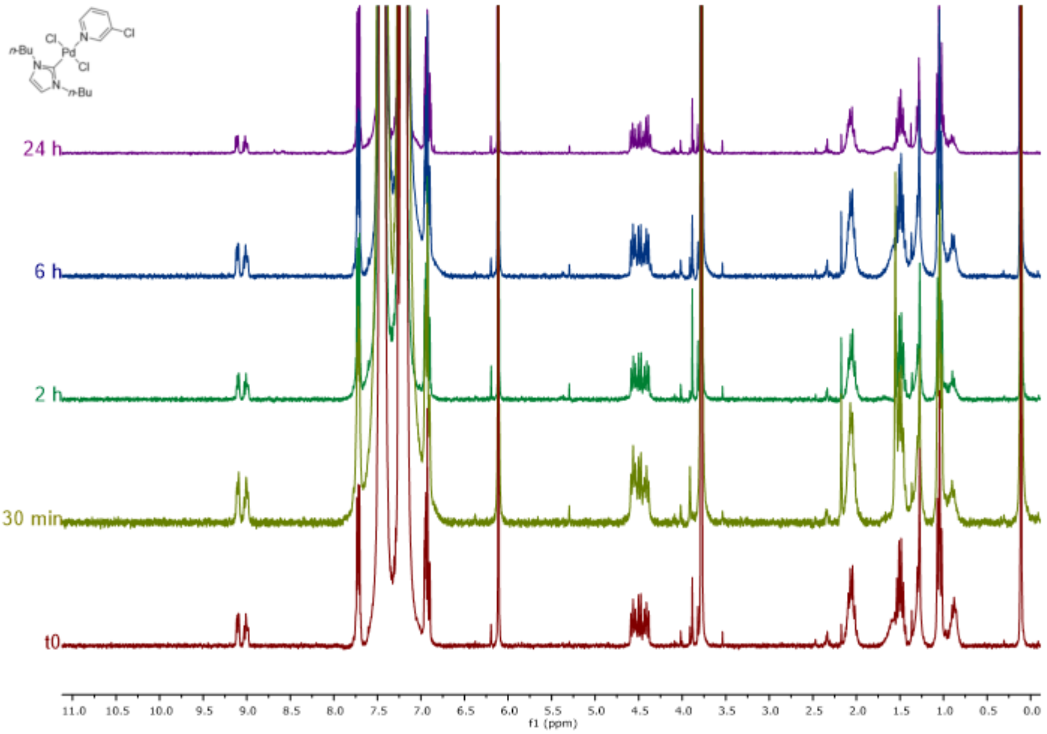

Figure S34. ^{1}H NMR spectra of samples (0, 30 min, 2, 6 and 24 h) from the reaction between **Pd-imi** and S_8 in dichlorobenzene at 120 °C, measured in $CDCl_3$.

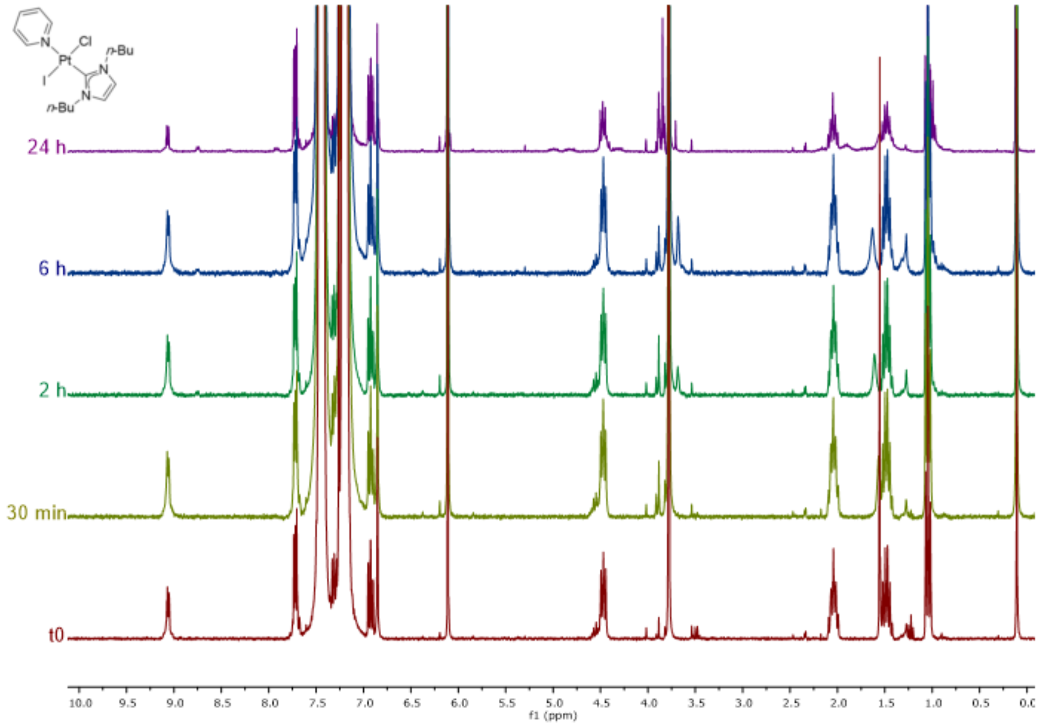

Figure S35. ^{1}H NMR spectra of samples (0, 30 min, 2, 6 and 24 h) from the reaction between **Pt-imi** and S_8 in dichlorobenzene at 120 °C, measured in $CDCl_3$.

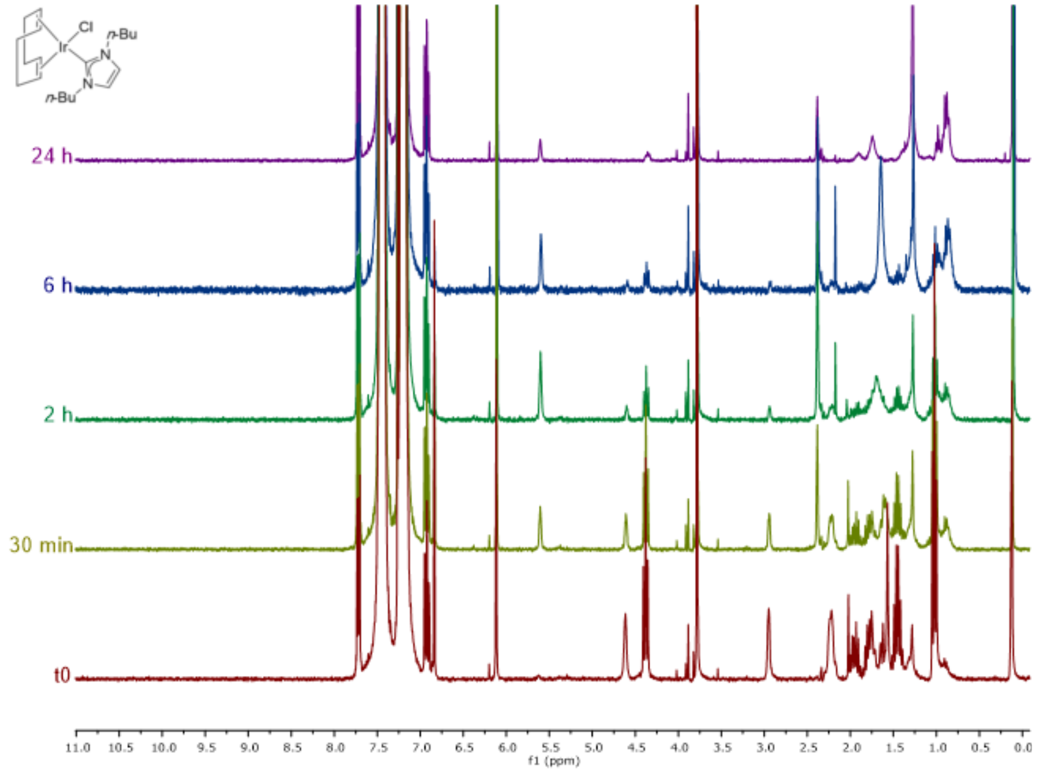

Figure S36. ^{1}H NMR spectra of samples (0, 30 min, 2, 6 and 24 h) from the reaction between **Ir(I)-imi** and S_8 in dichlorobenzene at 120 °C, measured in $CDCl_3$.

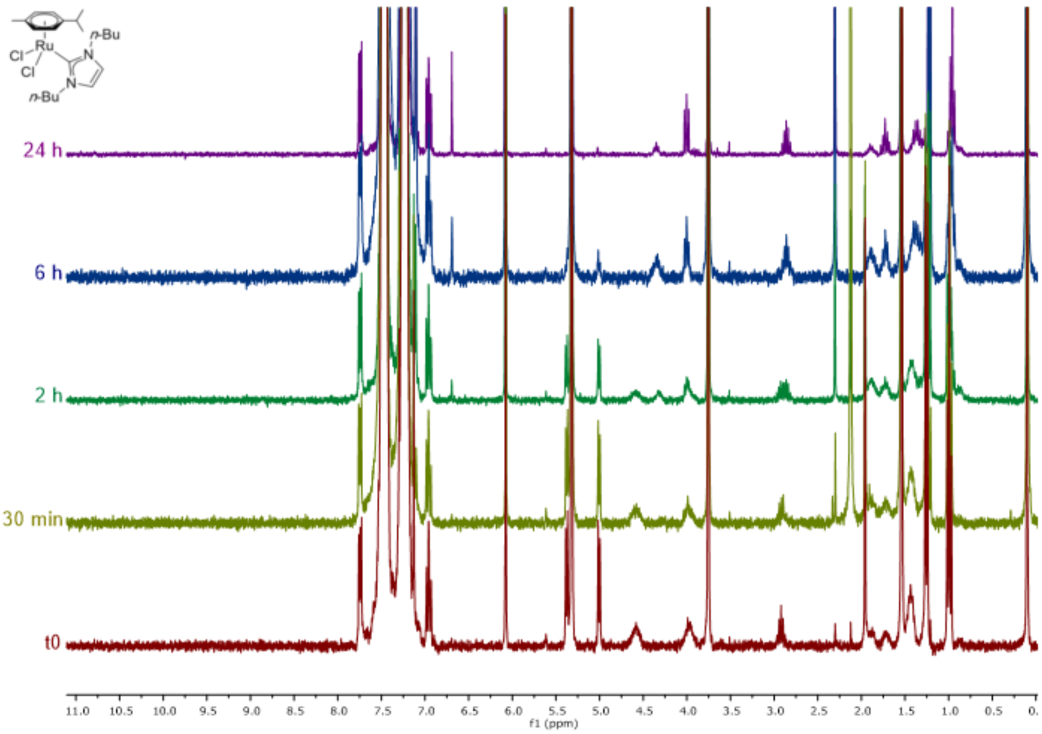

Figure S37. ^{1}H NMR spectra of samples (0, 30 min, 2, 6 and 24 h) from the reaction between **Ru-imi** and S_8 in dichlorobenzene at 120 °C, measured in CD_2Cl_2.

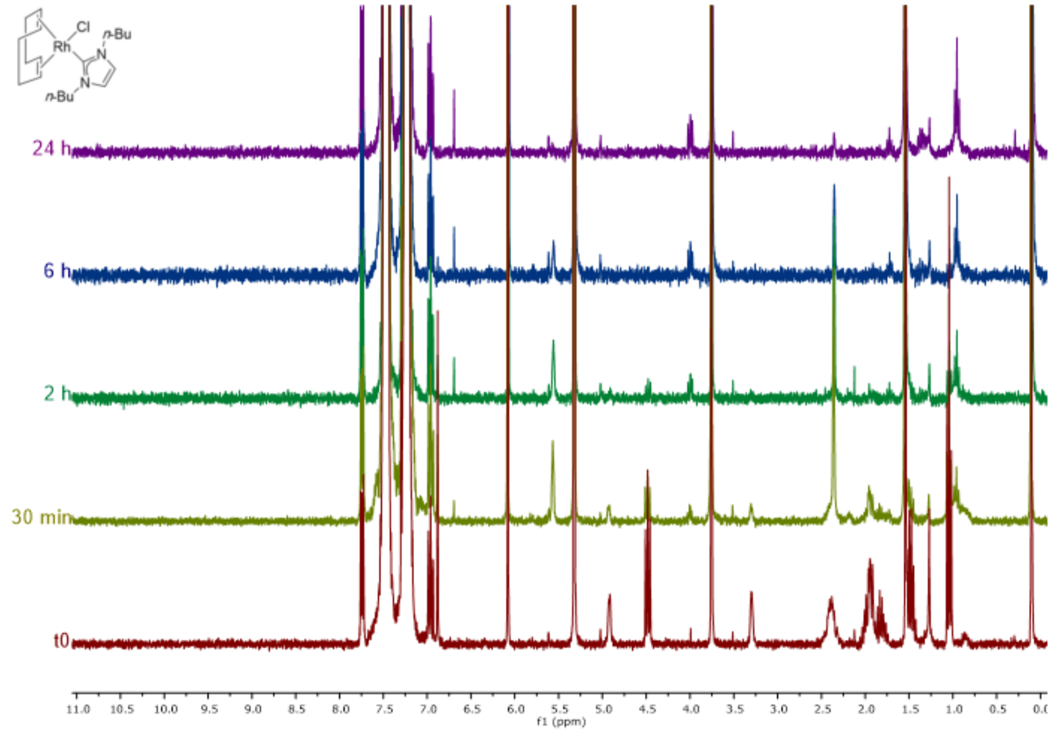

Figure S38. ^{1}H NMR spectra of samples (0, 30 min, 2, 6 and 24 h) from the reaction between **Rh(I)-imi** and S_8 in dichlorobenzene at 120 °C, measured in CD_2Cl_2.

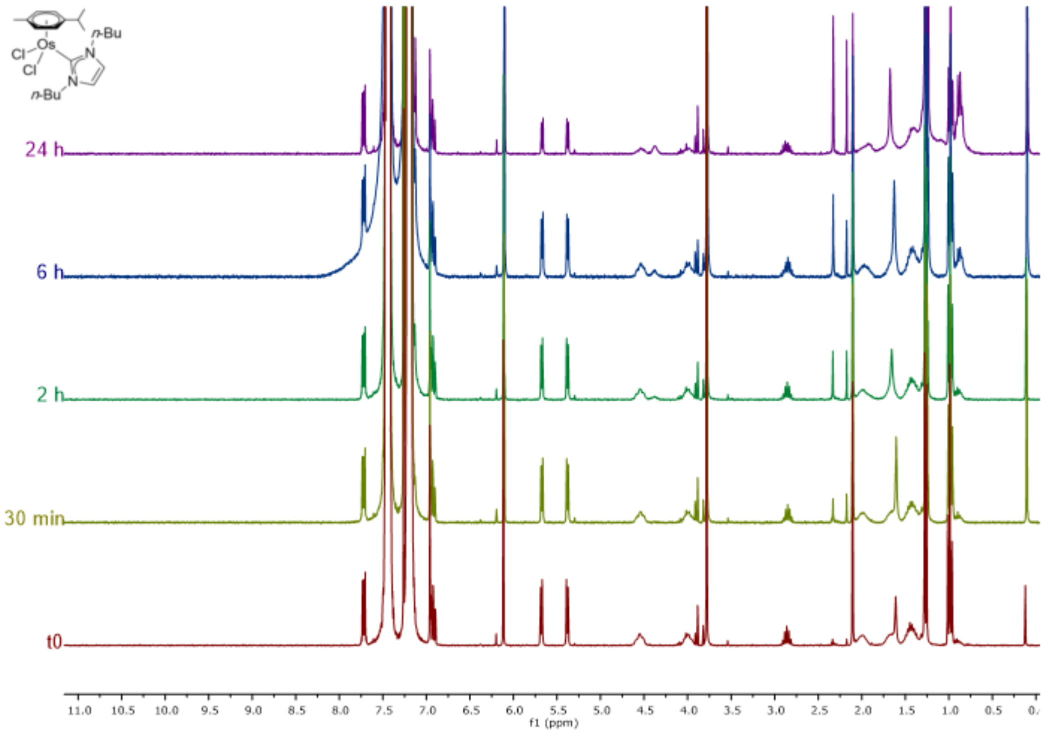

Figure S39. ^{1}H NMR spectra of samples (0, 30 min, 2, 6 and 24 h) from the reaction between **Os-imi** and S_8 in dichlorobenzene at 120 °C, measured in $CDCl_3$.

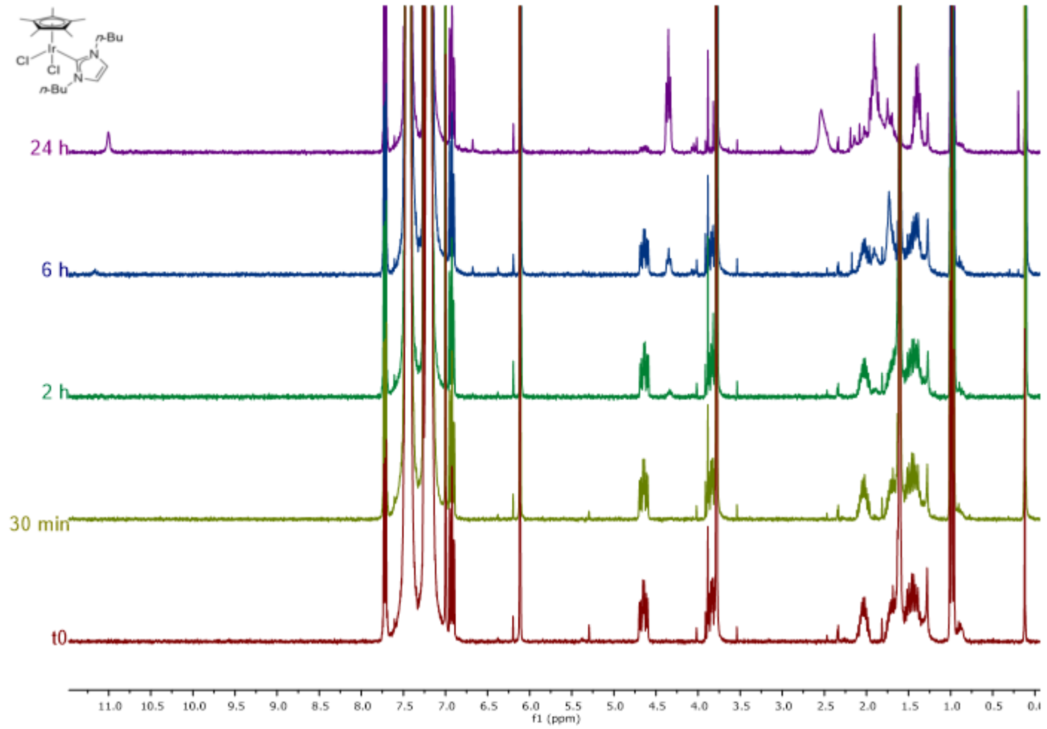

Figure S40. ^{1}H NMR spectra of samples (0, 30 min, 2, 6 and 24 h) from the reaction between **Ir(III)-imi** and S_8 in dichlorobenzene at 120 °C, measured in $CDCl_3$.

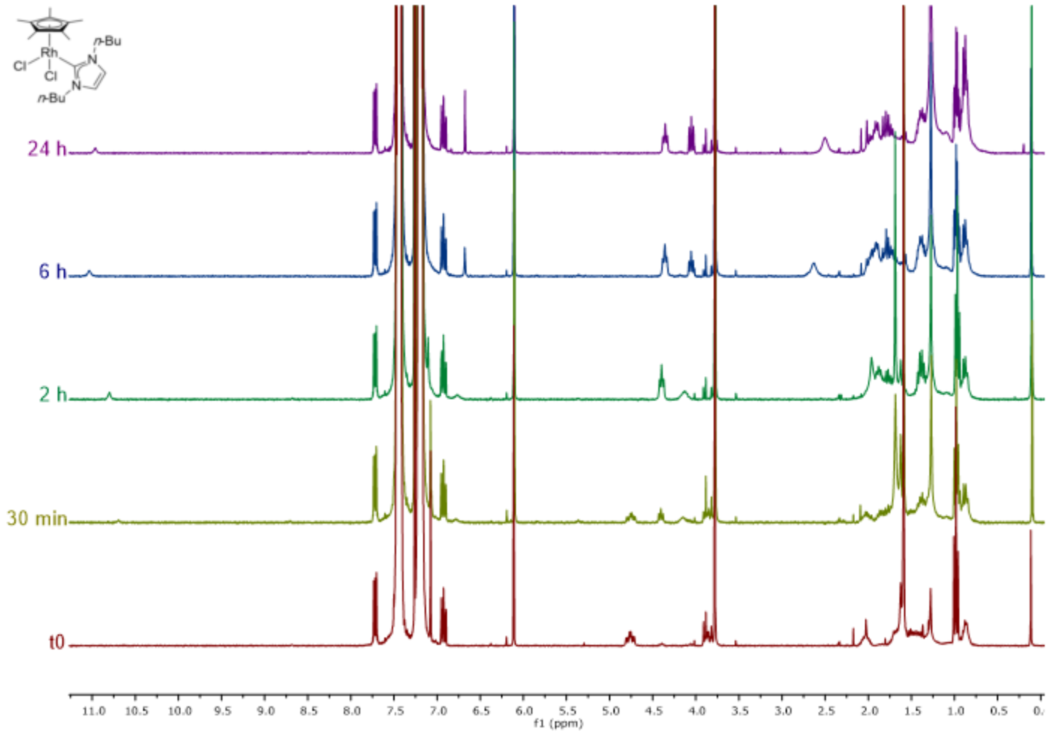

Figure S41. ^1H NMR spectra of samples (0, 30 min, 2, 6 and 24 h) from the reaction between **Rh(III)-imi** and S_8 in dichlorobenzene at 120 °C, measured in $CDCl_3$.

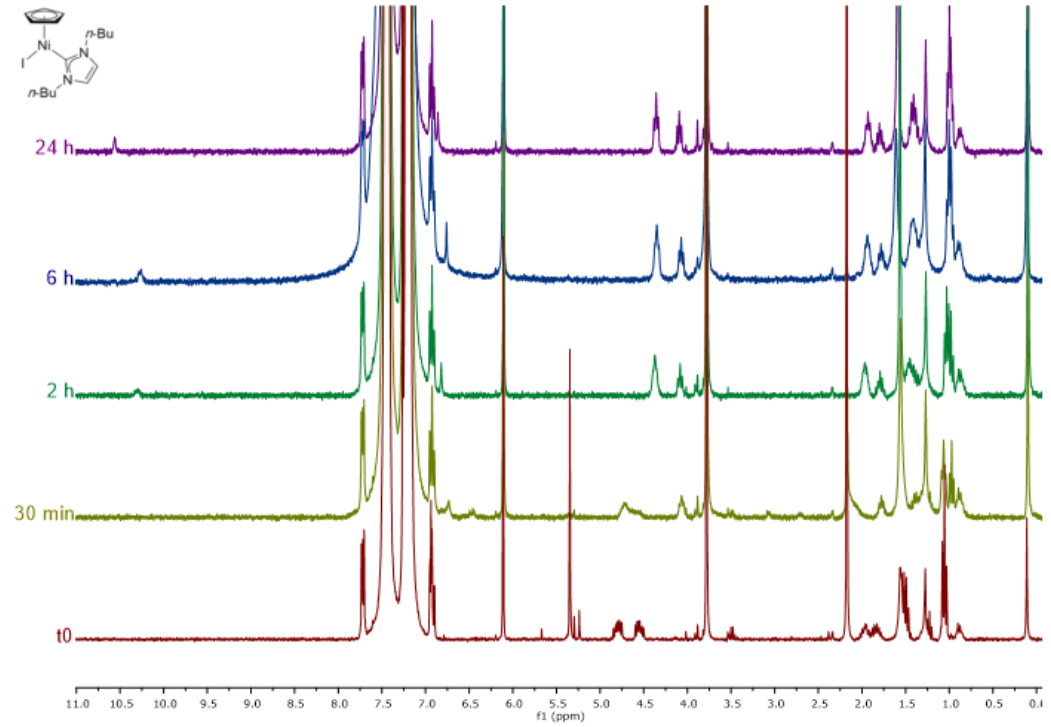

Figure S42. ^1H NMR spectra of samples (0, 30 min, 2, 6 and 24 h) from the reaction between **Ni-imi** and S_8 in dichlorobenzene at 120 °C, measured in $CDCl_3$.

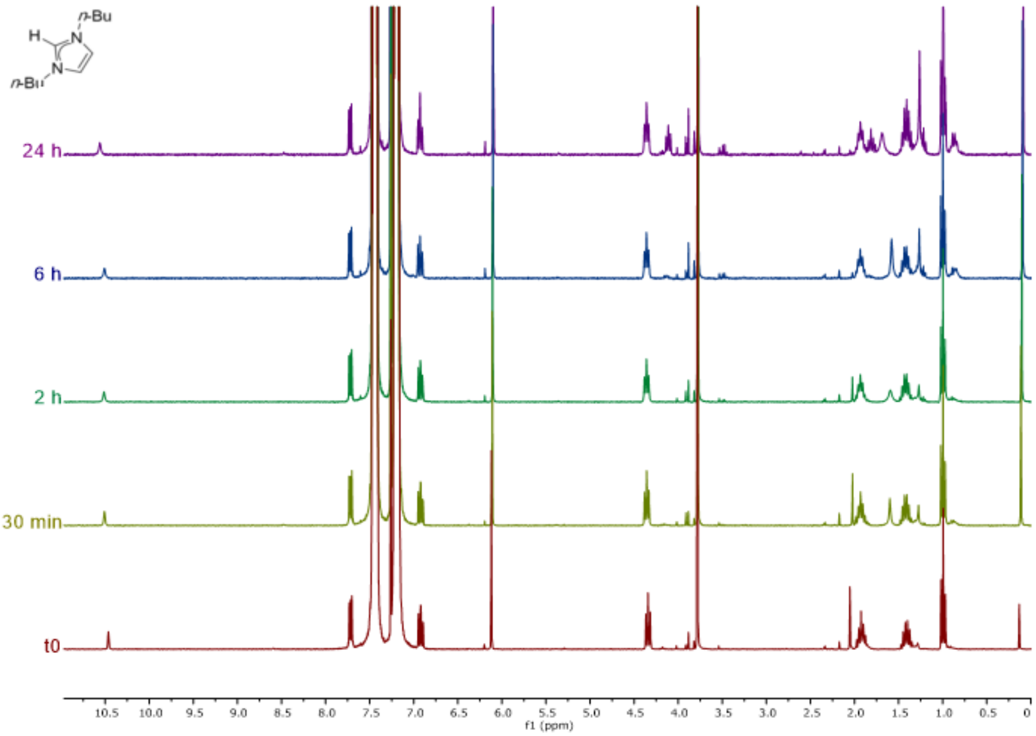

Figure S43. ^{1}H NMR spectra of samples (0, 30 min, 2, 6 and 24 h) from the reaction between **imi-H.I** and S_8 in dichlorobenzene at 120 °C, measured in $CDCl_3$.

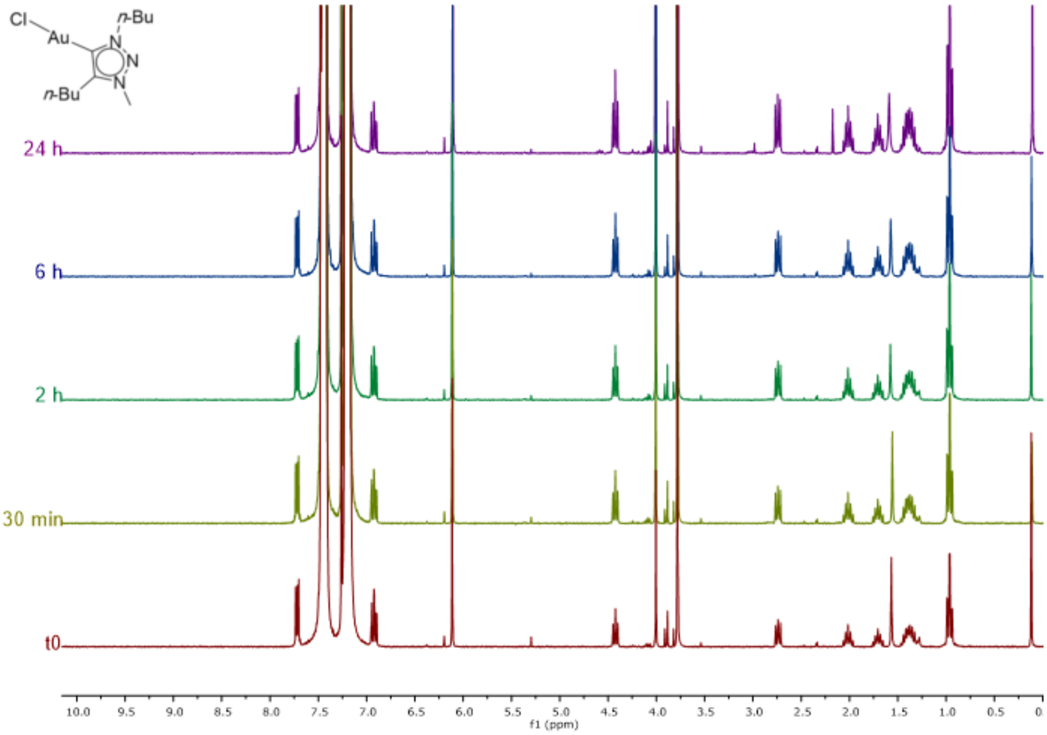

Figure S44. ^{1}H NMR spectra of samples (0, 30 min, 2, 6 and 24 h) from the reaction between **Au-trz** and S_8 in dichlorobenzene at 120 °C, measured in $CDCl_3$.

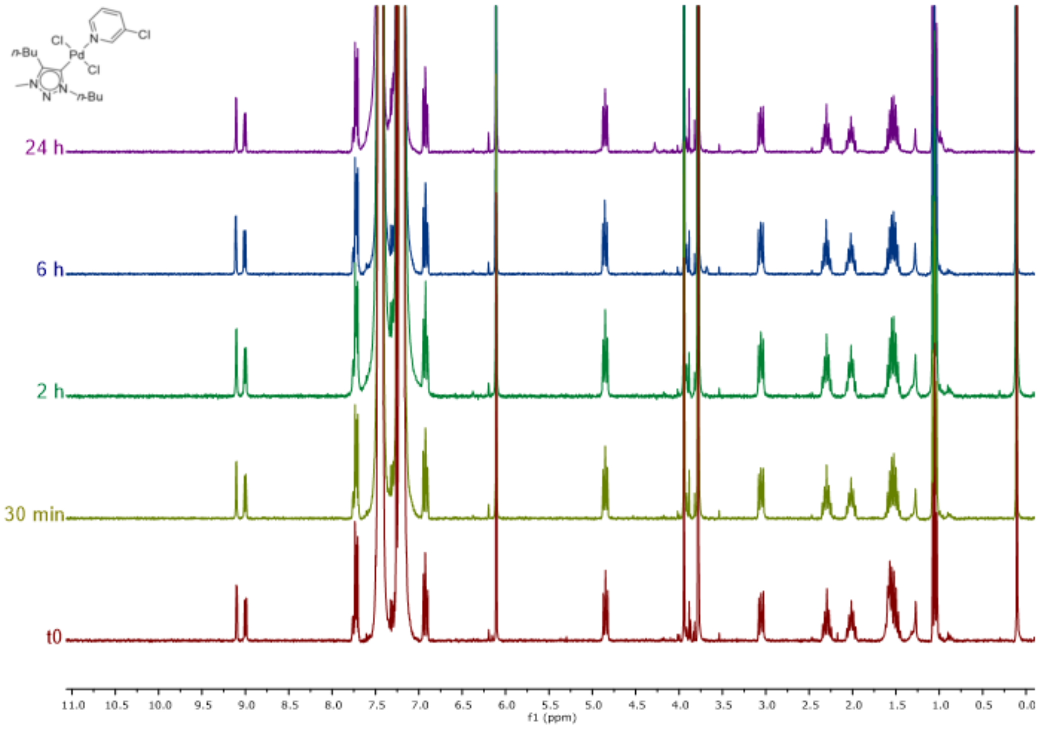

Figure S45. ^{1}H NMR spectra of samples (0, 30 min, 2, 6 and 24 h) from the reaction between **Pd-trz** and S_8 in dichlorobenzene at 120 °C, measured in $CDCl_3$.

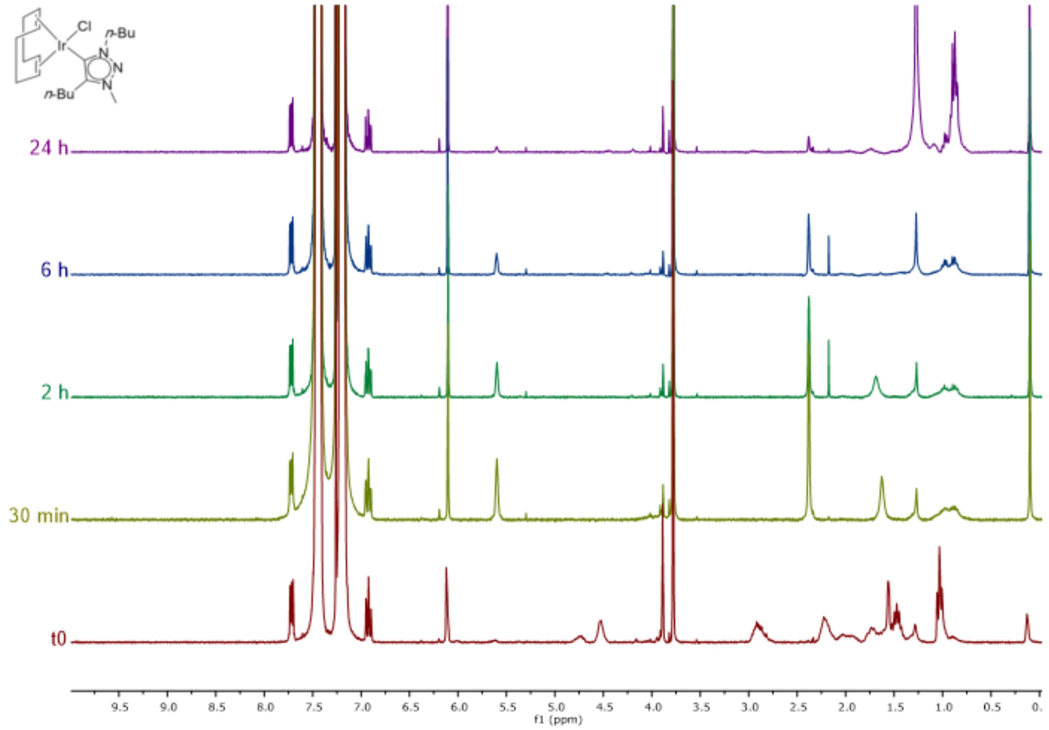

Figure S46. ^{1}H NMR spectra of samples (0, 30 min, 2, 6 and 24 h) from the reaction between **Ir(I)-trz** and S_8 in dichlorobenzene at 120 °C, measured in $CDCl_3$.

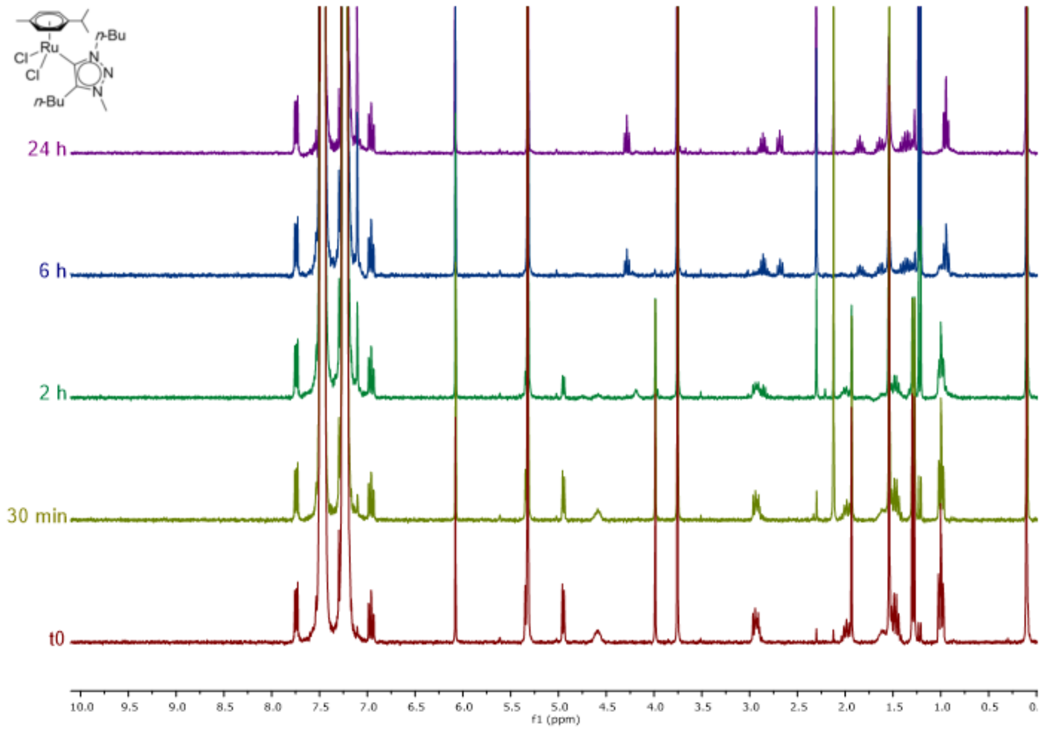

Figure S47. ^{1}H NMR spectra of samples (0, 30 min, 2, 6 and 24 h) from the reaction between **Ru-trz** and S_8 in dichlorobenzene at 120 °C, measured in CD_2Cl_2.

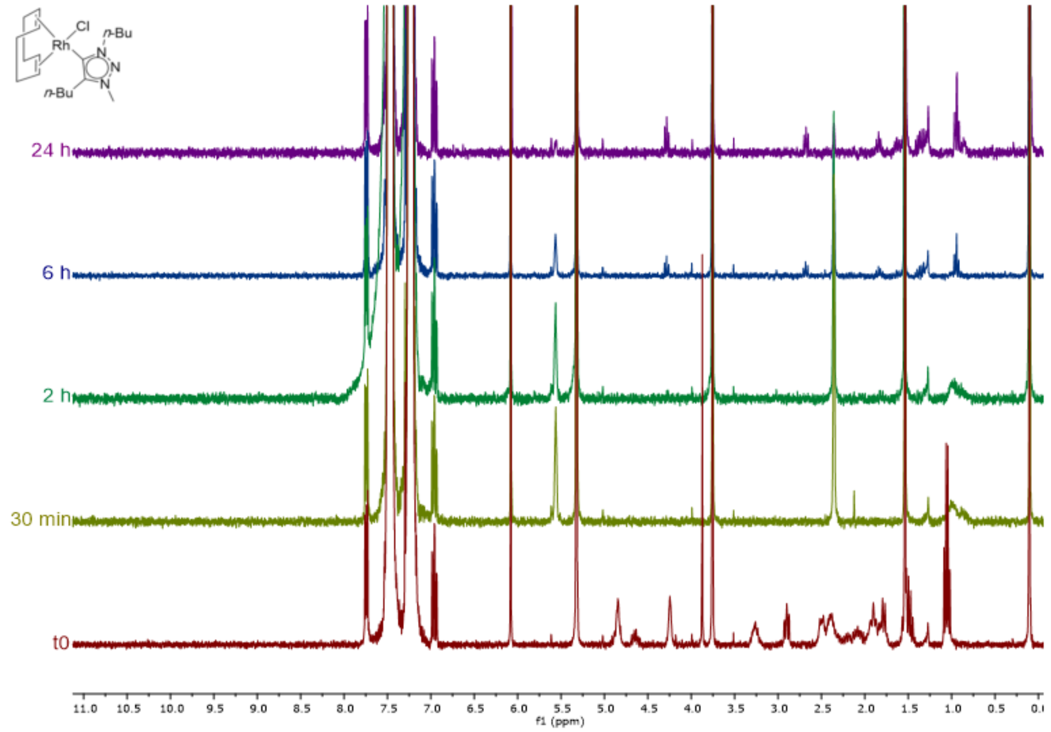

Figure S48. ^{1}H NMR spectra of samples (0, 30 min, 2, 6 and 24 h) from the reaction between **Rh(I)-trz** and S_8 in dichlorobenzene at 120 °C, measured in CD_2Cl_2.

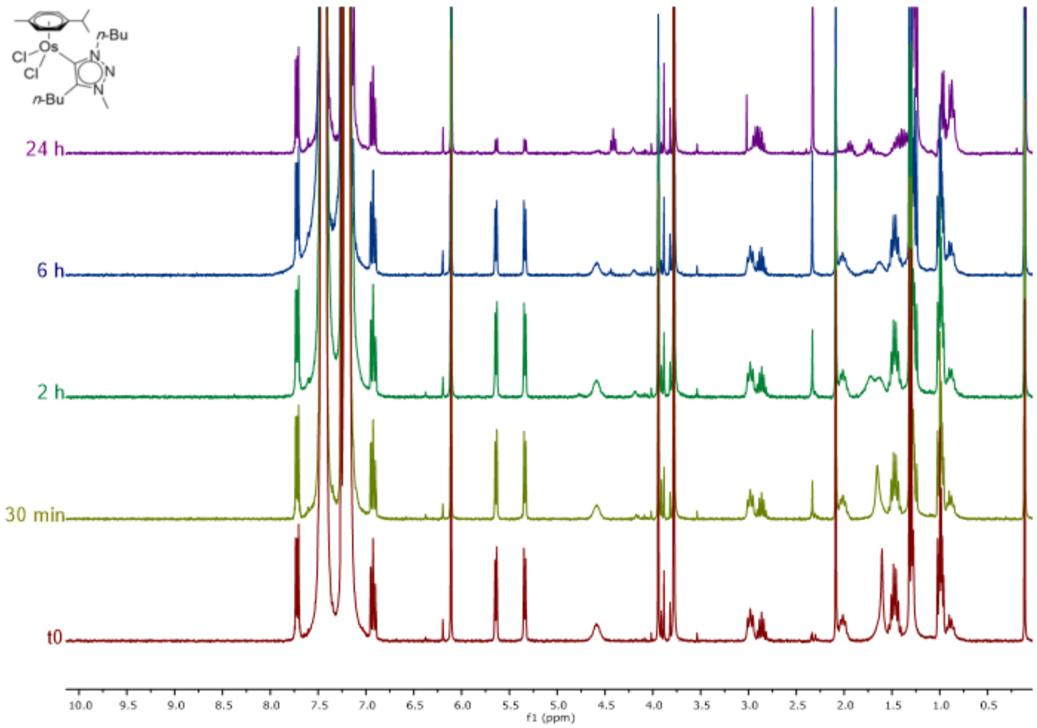

Figure S49. ^{1}H NMR spectra of samples (0, 30 min, 2, 6 and 24 h) from the reaction between **Os-trz** and S_8 in dichlorobenzene at 120 °C, measured in $CDCl_3$.

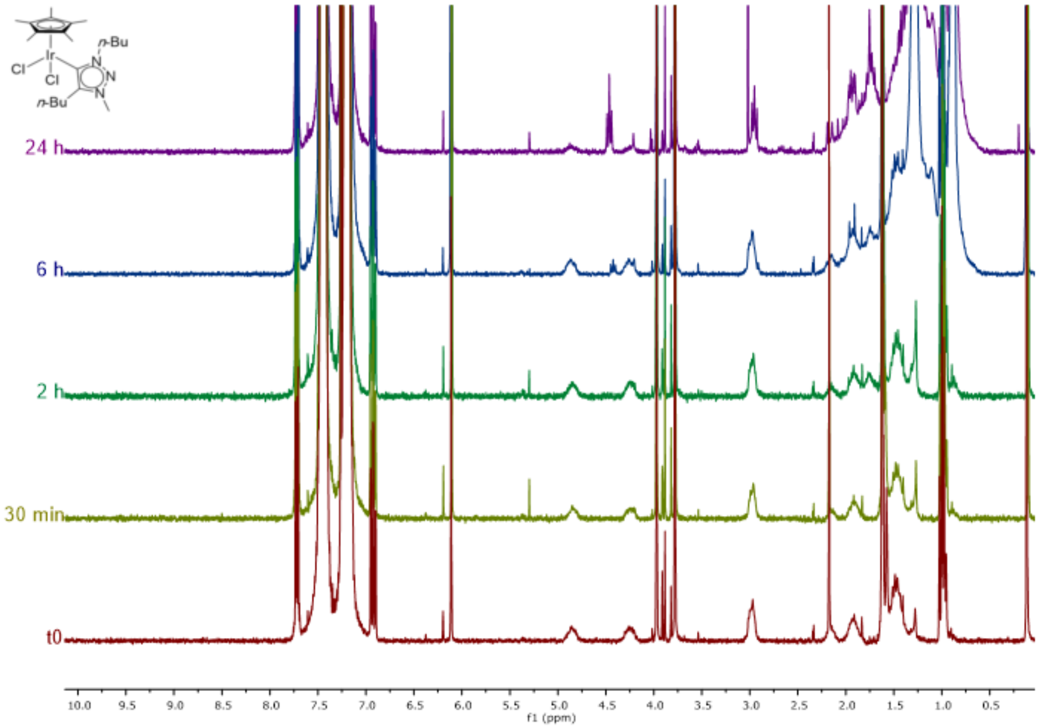

Figure S50. ^{1}H NMR spectra of samples (0, 30 min, 2, 6 and 24 h) from the reaction between **Ir(III)-trz** and S_8 in dichlorobenzene at 120 °C, measured in $CDCl_3$.

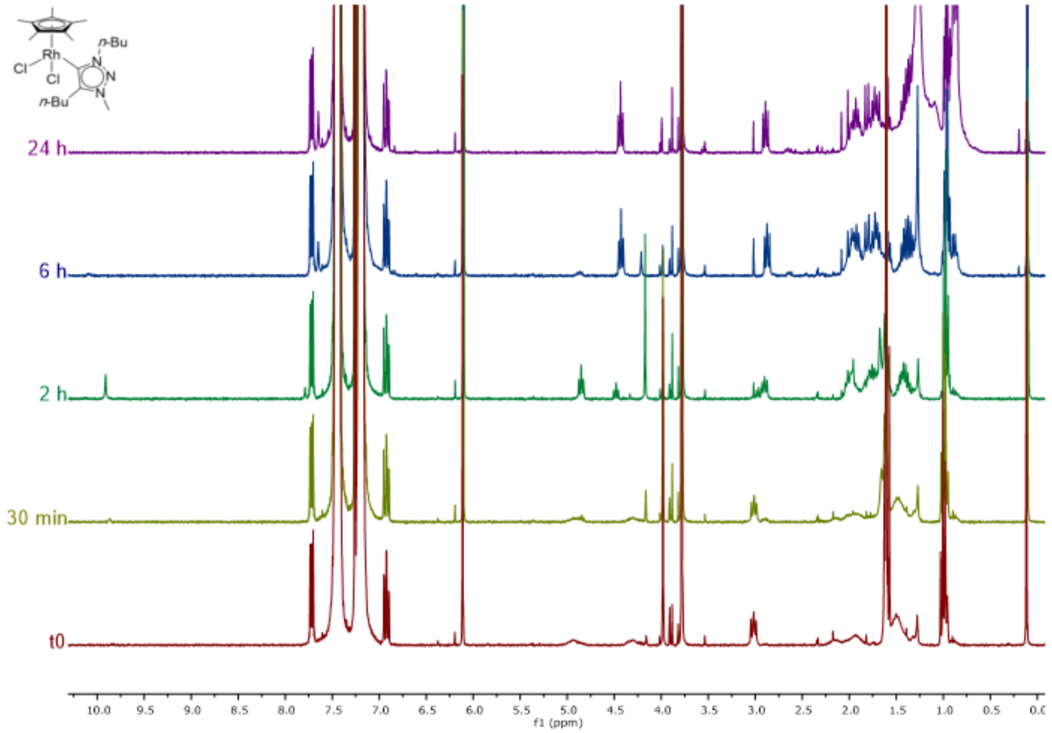

Figure S51. ¹H NMR spectra of samples (0, 30 min, 2, 6 and 24 h) from the reaction between **Rh(III)-trz** and S_8 in dichlorobenzene at 120 °C, measured in $CDCl_3$.

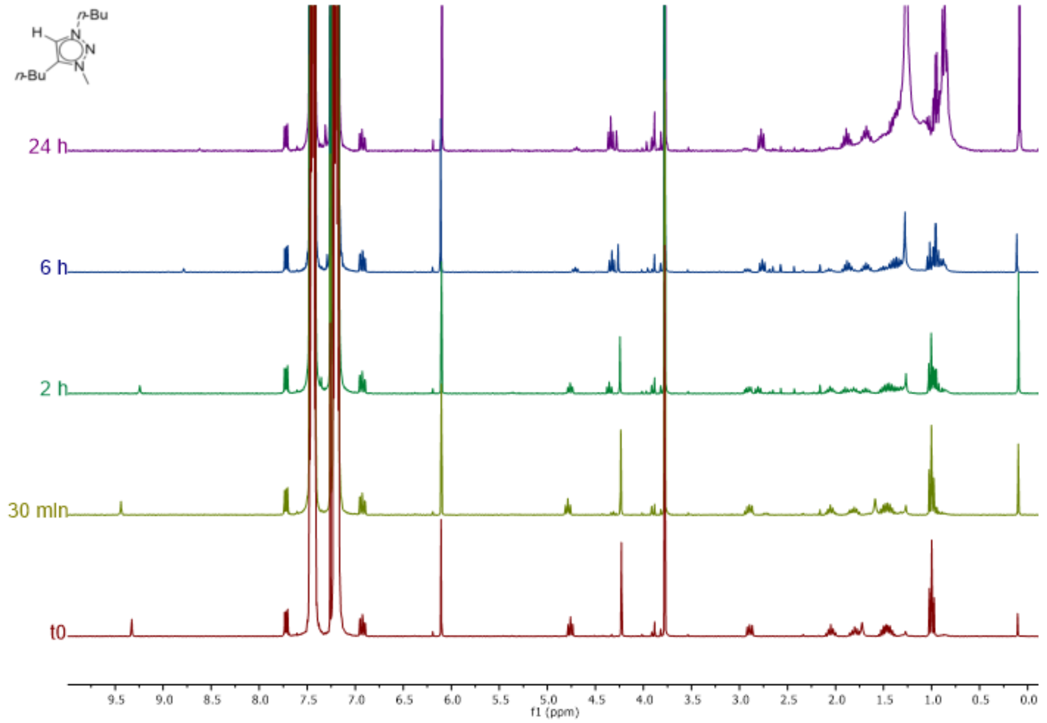

Figure S52. ¹H NMR spectra of samples (0, 30 min, 2, 6 and 24 h) from the reaction between **trz-H.I** and S_8 in dichlorobenzene at 120 °C, measured in $CDCl_3$.

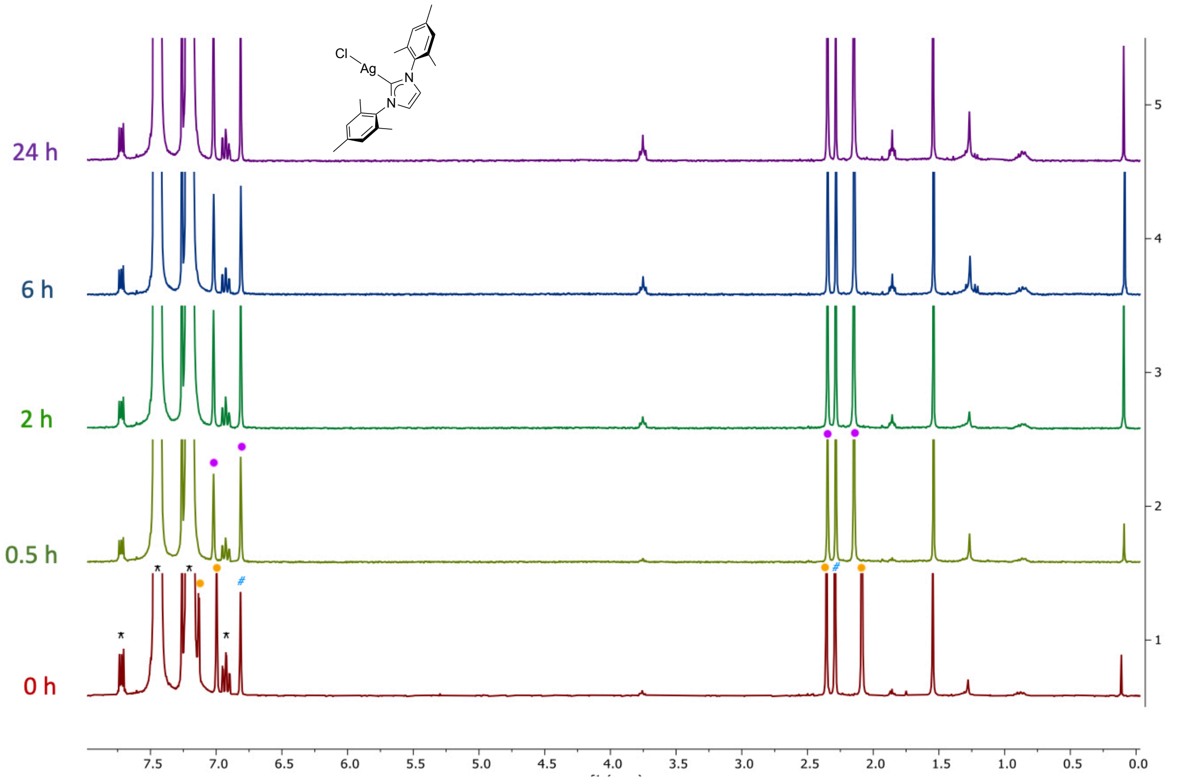

Figure S53. ^{1}H NMR spectra of samples (0, 30 min, 2, 6 and 24 h) from the reaction between **Ag-IMes** and S_8 in dichlorobenzene at 120 °C, measured in $CDCl_3$ (● Ag-IMes, ● IMes=S, # mesitylene (standard), * dichlorobenzene). Characteristic shifts are the C_{imi}–H from δ_H 7.13 to 6.81, and the *ortho* C_{Mes}–CH_3 from δ_H 2.08 to 2.15.

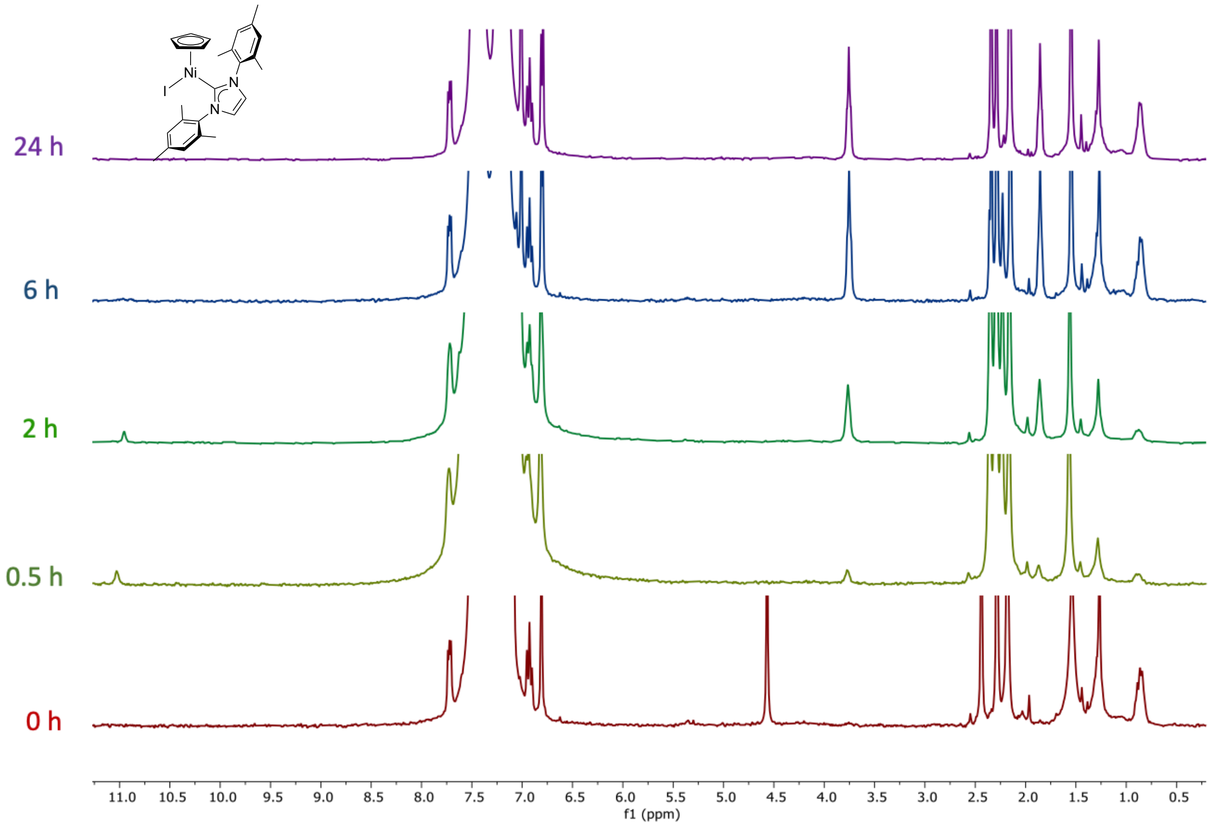

Figure S54. ^{1}H NMR spectra of samples (0, 30 min, 2, 6 and 24 h) from the reaction between **Ni-IMes** and S_8 in dichlorobenzene at 120 °C, measured in $CDCl_3$.

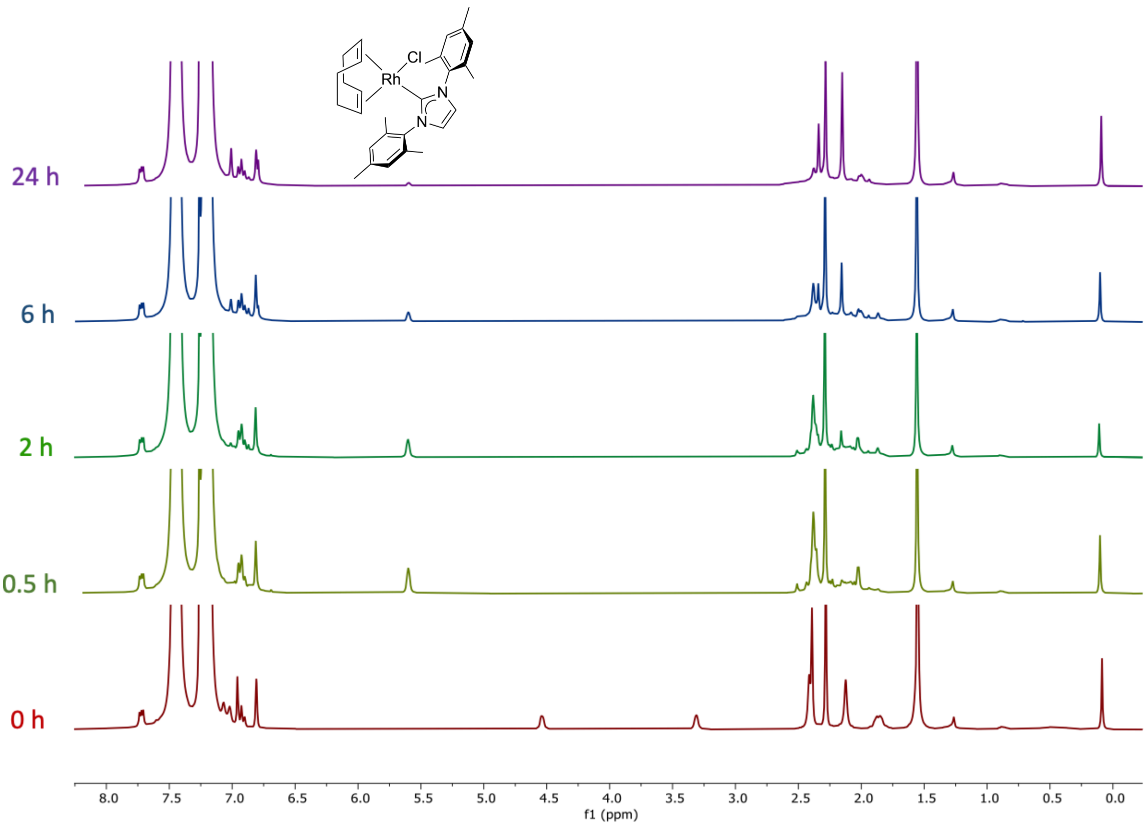

Figure S55. ^{1}H NMR spectra of samples (0, 30 min, 2, 6 and 24 h) from the reaction between **Rh(I)-IMes** and S_8 in dichlorobenzene at 120 °C, measured in $CDCl_3$.

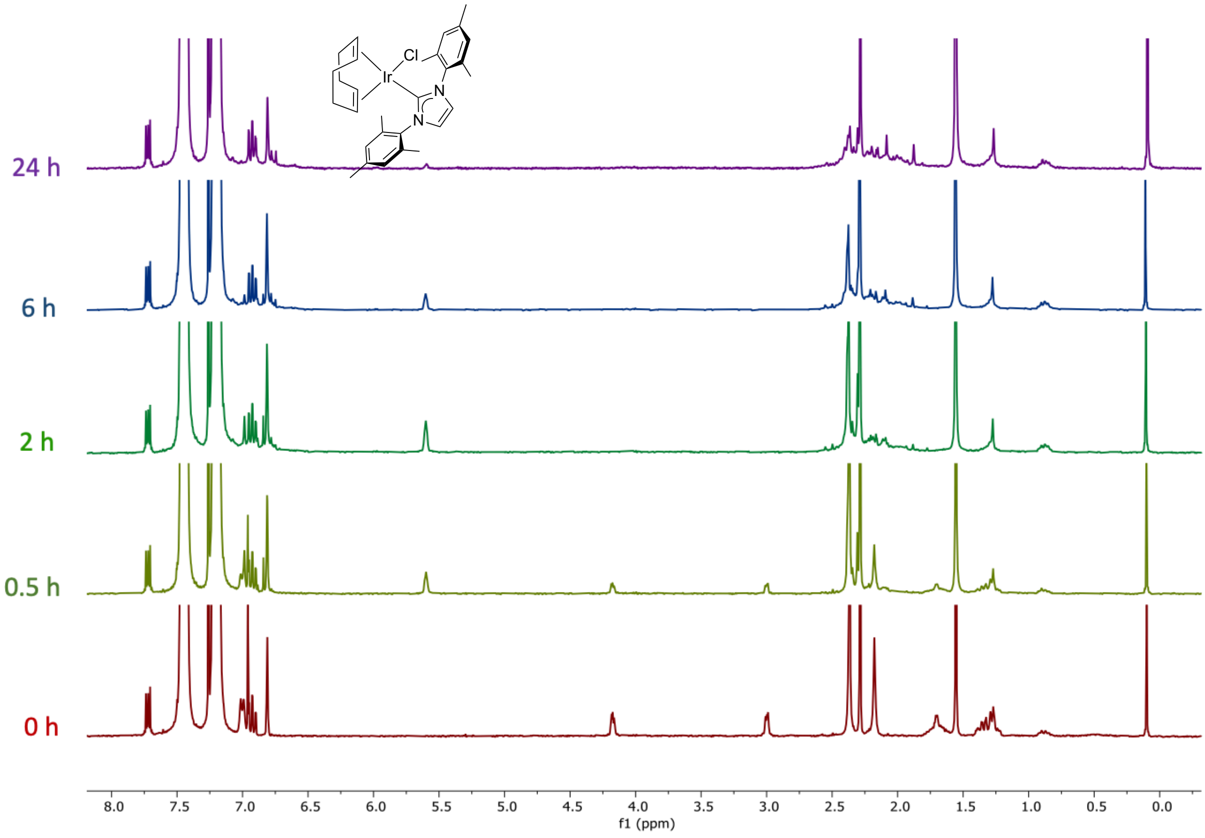

Figure S56. ^{1}H NMR spectra of samples (0, 30 min, 2, 6 and 24 h) from the reaction between **Ir(I)-IMes** and S_8 in dichlorobenzene at 120 °C, measured in $CDCl_3$.

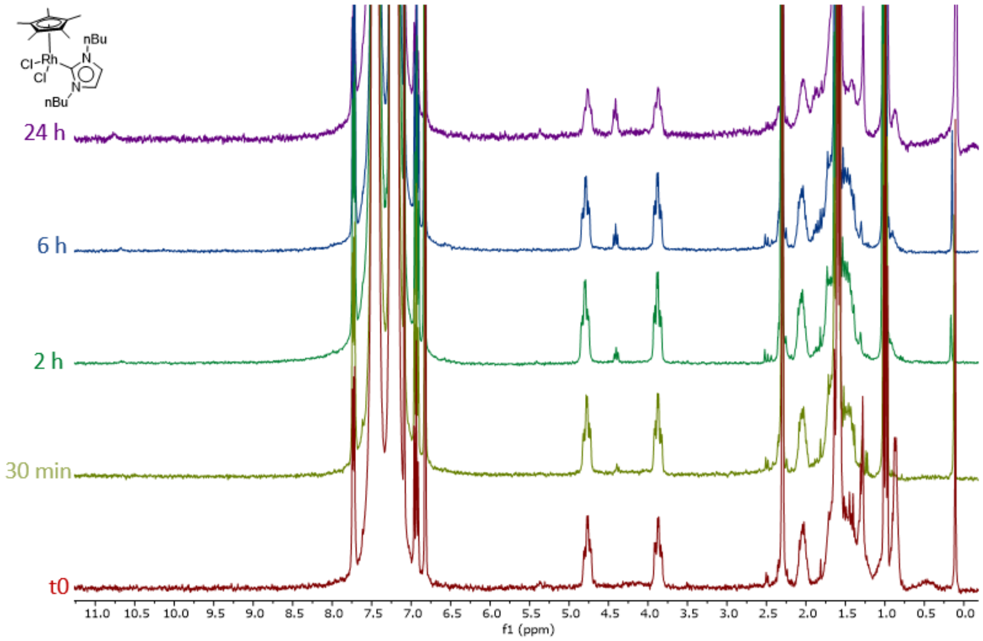

Figure S57. ^{1}H NMR spectra of samples (0, 30 min, 2, 6 and 24 h) from the reaction between **Rh(III)-imi** in dichlorobenzene at 120 °C in the absence of S_8. The decomposition is <3%, 4%, 7%, and 18% at 0.5, 1, 6, and 24 h, respectively.

6. Catalytic activity and stability of Ir(III)–trz

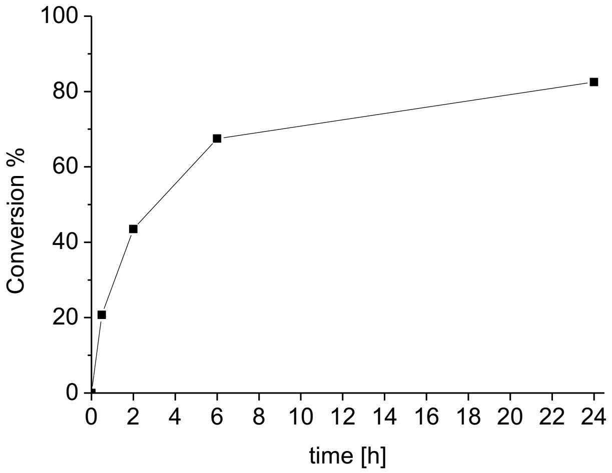

Figure S58. Catalytic activity of **Ir(III)-trz** in the transfer hydrogenation of benzophenone to diphenylmethanol (reaction conditions: 0.5 mmol benzophenone, 0.05 mmol KOH, 5mmol **Ir(III)-trz**, 5 mL *i*PrOH, reflux. Conversion determined from aliquots diluted in $CDCl_3$ and analyzed by ^{1}H NMR spectroscopy).

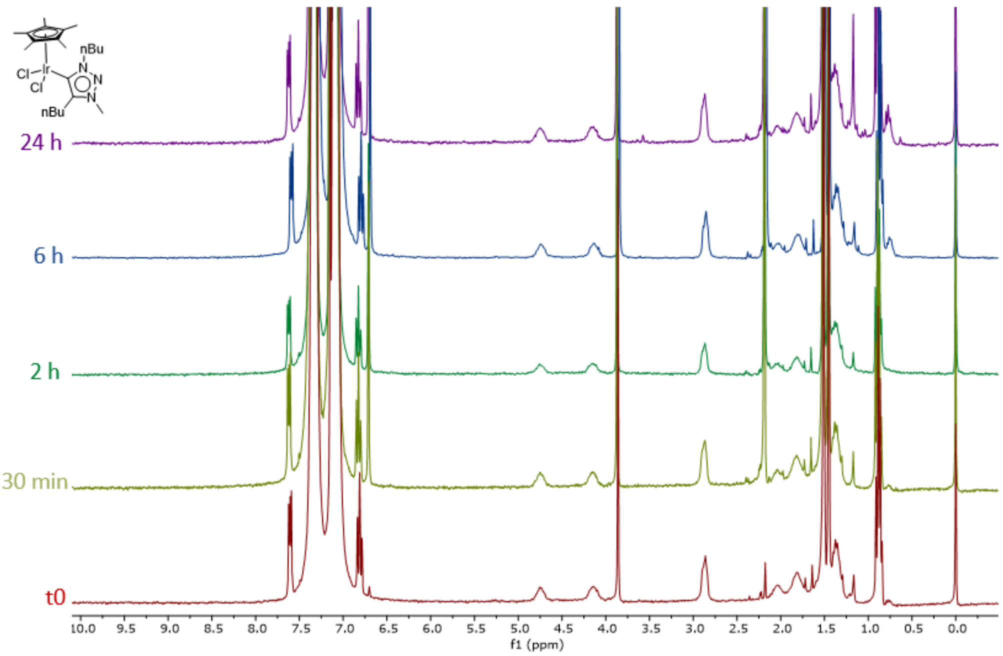

Figure S59. ^{1}H NMR spectra of samples (0, 30 min, 2, 6 and 24 h) from the reaction between **Ir(III)-trz** and S_8 in dichlorobenzene at 80 °C, *i.e.* the temperature used for transfer hydrogenation (see Fig. S61), measured in $CDCl_3$. The monitoring shows no detectable degradation under these conditions.

7. References

S1 V. Ritleng, E. Brenner and M. J. Chetcuti, *J. Chem. Educ.*, 2008, **85**, 1646.

S2 M. A. Bennett and A. K. Smith, *J. Chem. Soc., Dalton Trans.*, 1974, 233–241.

S3 H. Werner and K. Zenkert, *J. Organomet. Chem.*, 1988, **345**, 151–166.

S4 R. H. Crabtree, J. M. Quirk, H. Felkin and T. Fillebeen-khan, *Synth. React. Inorg. Met. Chem.*, 1982, **12**, 407–413.

S5 G. Giordano and R. H. Crabtree, *Inorg. Synth.*, 1979, **19**, 218.

S6 C. White, A. Yates, P. M. Maitlis and D. M. Heinekey, *Inorg. Synth.*, 1992, **29**, 228–234.

S7 S. S. Palimkar, S. A. Siddiqui, T. Daniel, R. J. Lahoti and K. V Srinivasan, *J. Org. Chem.*, 2003, **68**, 9371–9378.

S8 A. Poulain, D. Canseco-Gonzalez, R. Hynes-Roche, H. Müller-Bunz, O. Schuster, H. Stoeckli-Evans, A. Neels and M. Albrecht, *Organometallics*, 2011, **30**, 1021–1029.

S9 K. Mebrouk, F. Camerel, O. Jeannin, B. Heinrich, B. Donnio and M. Fourmigué, *Inorg. Chem.*, 2016, **55**, 1296–1303.

S10 A. R. Chianese, X. Li, M. C. Janzen, J. W. Faller and R. H. Crabtree, *Organometallics*, 2003, **22**, 1663–1667.

S11 R. Corberán, M. Sanaú and E. Peris, *J. Am. Chem. Soc.*, 2006, **128**, 3974–3979.

S12 S. Y. Choi and Y. K. Chung, *Adv. Synth. Catal.*, 2011, **353**, 2609–2613.

S13 L. Mercs, A. Neels and M. Albrecht, *Dalton Trans.*, 2008, 5570–5576.

S14 A. Prades, E. Peris and M. Albrecht, *Organometallics*, 2011, **30**, 1162–1167.

S15 U. Hintermair, U. Englert and W. Leitner, *Organometallics*, 2011, **30**, 3726–3731.

S16 D. Canseco-Gonzalez and M. Albrecht, *Dalton Trans.*, 2013, **42**, 7424–7432.

S17 M. V Baker, P. J. Barnard, S. J. Berners-Price, S. K. Brayshaw, J. L. Hickey, B. W. Skelton and A. H. White, *J. Organomet. Chem.*, 2005, **690**, 5625–5635.

S18 R. Pretorius, M. R. Fructos, H. Müller-Bunz, R. A. Gossage, P. J. Pérez and M. Albrecht, *Dalton Trans.*, 2016, **45**, 14591–14602.

S19 H. Valdés, M. Poyatos, G. Ujaque and E. Peris, *Chem. Eur. J.*, 2015, **21**, 1578–1588.

S20 D. Canseco-Gonzalez, A. Gniewek, M. Szulmanowicz, H. Müller-Bunz, A. M. Trzeciak and M. Albrecht, *Chem. Eur. J.*, 2012, **18**, 6055–6062.

S21 T. Ramnial, C. D. Abernethy, M. D. Spicer, I. D. McKenzie, I. D. Gay and J. A. C. Clyburne *Inorg. Chem.*, 2003, **42**, 1391–1393.

S22 C. D Abernethy, A. H. Cowley and R. A. Jones, J. Organomet. Chem., 2000, **596**, 3–5.

S23 X. Yu, B. O. Patrick and B. R. James, *Organometallics*, 2006, **25**, 2359–2363.

S24 R. A. Kelly III, H. Clavier, S. Giudice, N. M. Scott, E. D. Stevens, J. Bordner, I. Samardjiev, C. D. Hoff, L. Cavallo and S. P. Nolan, *Organometallics*, 2008, **27**, 202–210. D. S. Timofeeva, D. M. Lindsay, D. J. Nelson and W. J. Kerr, *Catal. Sci. Technol.*, 2020, **10**, 7249–7255.
